# Admixture Mapping in Lupus Identifies Multiple Functional Variants within IFIH1 Associated with Apoptosis, Inflammation, and Autoantibody Production

**DOI:** 10.1371/journal.pgen.1003222

**Published:** 2013-02-18

**Authors:** Julio E. Molineros, Amit K. Maiti, Celi Sun, Loren L. Looger, Shizhong Han, Xana Kim-Howard, Stuart Glenn, Adam Adler, Jennifer A. Kelly, Timothy B. Niewold, Gary S. Gilkeson, Elizabeth E. Brown, Graciela S. Alarcón, Jeffrey C. Edberg, Michelle Petri, Rosalind Ramsey-Goldman, John D. Reveille, Luis M. Vilá, Barry I. Freedman, Betty P. Tsao, Lindsey A. Criswell, Chaim O. Jacob, Jason H. Moore, Timothy J. Vyse, Carl L. Langefeld, Joel M. Guthridge, Patrick M. Gaffney, Kathy L. Moser, R. Hal Scofield, Marta E. Alarcón-Riquelme, on behalf of the BIOLUPUS Network, Scott M. Williams, Joan T. Merrill, Judith A. James, Kenneth M. Kaufman, Robert P. Kimberly, John B. Harley, Swapan K. Nath

**Affiliations:** 1 Arthritis and Clinical Immunology Research Program, Oklahoma Medical Research Foundation, Oklahoma City, Oklahoma, United States of America; 2 Howard Hughes Medical Institute, Janelia Farm Research Campus, Ashburn, Virginia, United States of America; 3 Department of Psychiatry, Yale School of Medicine, New Haven, Connecticut, United States of America; 4 Mayo Clinic, Division of Rheumatology and Department of Immunology, Rochester, Minnesota, United States of America; 5 Division of Rheumatology, Medical University of South Carolina, Charleston, South Carolina, United States of America; 6 Department of Medicine, University of Alabama at Birmingham, Birmingham, Alabama, United States of America; 7 Department of Medicine, Johns Hopkins University School of Medicine, Baltimore, Maryland, United States of America; 8 Division of Rheumatology, Northwestern University Feinberg School of Medicine, Chicago, Illinois, United States of America; 9 Department of Rheumatology and Clinical Immunogenetics, University of Texas Health Science Center at Houston, Houston, Texas, United States of America; 10 Department of Medicine, Division of Rheumatology, University of Puerto Rico Medical Sciences Campus, San Juan, Puerto Rico; 11 Department of Internal Medicine, Wake Forest School of Medicine, Winston-Salem, North Carolina, United States of America; 12 Division of Rheumatology, Department of Medicine, University of California Los Angeles, Los Angeles, California, United States of America; 13 Rosalind Russell Medical Research Center for Arthritis, University of California San Francisco, San Francisco, California, United States of America; 14 Department of Medicine, University of Southern California, Los Angeles, California, United States of America; 15 Department of Genetics, Dartmouth Medical School, Lebanon, New Hampshire, United States of America; 16 Division of Genetics and Molecular Medicine, King's College London, London, United Kingdom; 17 Division of Immunology, Infection and Inflammatory Diseases, Kings College London, London, United Kingdom; 18 Department of Biostatistical Sciences, Wake Forest University Health Sciences, Wake Forest, North Carolina, United States of America; 19 College of Medicine, University of Oklahoma Health Sciences Center, Oklahoma City, Oklahoma, United States of America; 20 Centro de Genómica e Investigación Oncológica (GENyO)–Pfizer/Universidad de Granada/Junta de Andalucía, Granada, Spain; 21 Department of Genetics, Geisel School of Medicine, Dartmouth College, Hanover, New Hampshire, United States of America; 22 Clinical Pharmacology Research Program, Oklahoma Medical Research Foundation, Oklahoma City, Oklahoma, United States of America; 23 Cincinnati Children's Hospital Medical Center and the U.S. Department of Veterans Affairs Medical Center, Cincinnati, Ohio, United States of America; University of Oxford, United Kingdom

## Abstract

Systemic lupus erythematosus (SLE) is an inflammatory autoimmune disease with a strong genetic component. African-Americans (AA) are at increased risk of SLE, but the genetic basis of this risk is largely unknown. To identify causal variants in SLE loci in AA, we performed admixture mapping followed by fine mapping in AA and European-Americans (EA). Through genome-wide admixture mapping in AA, we identified a strong SLE susceptibility locus at 2q22–24 (LOD = 6.28), and the admixture signal is associated with the European ancestry (ancestry risk ratio ∼1.5). Large-scale genotypic analysis on 19,726 individuals of African and European ancestry revealed three independently associated variants in the *IFIH1 *gene: an intronic variant, rs13023380 [P*_meta_* = 5.20×10^−14^; odds ratio, 95% confidence interval = 0.82 (0.78–0.87)], and two missense variants, rs1990760 (Ala946Thr) [P*_meta_* = 3.08×10^−7^; 0.88 (0.84–0.93)] and rs10930046 (Arg460His) [P*_dom_* = 1.16×10^−8^; 0.70 (0.62–0.79)]. Both missense variants produced dramatic phenotypic changes in apoptosis and inflammation-related gene expression. We experimentally validated function of the intronic SNP by DNA electrophoresis, protein identification, and *in vitro* protein binding assays. DNA carrying the intronic risk allele rs13023380 showed reduced binding efficiency to a cellular protein complex including nucleolin and lupus autoantigen Ku70/80, and showed reduced transcriptional activity *in vivo*. Thus, in SLE patients, genetic susceptibility could create a biochemical imbalance that dysregulates nucleolin, Ku70/80, or other nucleic acid regulatory proteins. This could promote antibody hypermutation and auto-antibody generation, further destabilizing the cellular network. Together with molecular modeling, our results establish a distinct role for *IFIH1* in apoptosis, inflammation, and autoantibody production, and explain the molecular basis of these three risk alleles for SLE pathogenesis.

## Author Summary

African-Americans (AA) are at increased risk of systemic lupus erythematosus (SLE), but the genetic basis of this risk increase is largely unknown. We used admixture mapping to localize disease-causing genetic variants that differ in frequency across populations. This approach is advantageous for localizing susceptibility genes in recently admixed populations like AA. Our genome-wide admixture scan identified seven admixture signals, and we followed the best signal at 2q22–24 with fine-mapping, imputation-based association analysis and experimental validation. We identified two independent coding variants and a non-coding variant within the *IFIH1* gene associated with SLE. Together with molecular modeling, our results establish a distinct role for *IFIH1* in apoptosis, inflammation, and autoantibody production, and explain the molecular basis of these three risk alleles for SLE pathogenesis.

## Introduction

Systemic lupus erythematosus (SLE, [MIM 152700]) is a clinically heterogeneous autoimmune disease with a strong genetic component, characterized by inflammation, dysregulation of type-1 interferon responses and autoantibodies directed towards nuclear components. SLE overwhelmingly targets women, and its incidence and clinical course differ dramatically between ethnic populations. In particular, SLE occurs with at least 3–5 times higher prevalence and more severe complications in African-Americans (AA) compared to Americans with European ancestry (EA) [1]. However, the genetic basis of this increased risk is largely unknown. The recently “admixed” AA population is likely to provide critical information necessary to identify chromosomal regions that harbor variants associated with SLE and provide insights about allele frequency differences among distinct ancestral populations (*i.e.*, European and African). Admixture mapping (AM) has proven to be a powerful method to leverage ancestry information to identify chromosomal segments linked to disease [2]–[9]. For instance, AM has helped identify the risk gene *MYH9* in idiopathic focal segmental glomerulosclerosis in AA [10], and risk alleles in several genes associated with breast [11] and prostate cancer [12]. In addition to the greater lupus incidence, studying AA populations offers a second advantage. Africans have the smallest haplotype blocks of all human populations: African average population recombination distance is 6 kb, while it is 22 kb in Europeans and Asians [13], [14]. This 3-fold smaller haplotype size gives rise to correspondingly tighter genomic associations in admixed populations such as AA, making causal mutations easier to decipher.

Although several genes for SLE susceptibility have been found through candidate gene analysis and genome wide association scans (GWAS), none or very few causal mutations have been identified in each gene. In this study we employed AM in AA to identify admixture signals, and performed a follow-up association study on AA and EA to further identify and localize variants associated with SLE. We experimentally validated predicted variants with biochemistry, cell culture experiments and sequencing of patient-isolated samples. We showed distinct functions of two coding SNPs including changes in gene expression. Through electrophoretic mobility shift assays (EMSAs), protein identification and *in vitro* protein binding assays, we determined that the intronic SNP disrupts function of a transcriptional enhancer of the *IFIH1* locus. Taken together, these results explain the effects of three independent causal mutations on SLE, and begin to elucidate the disparity in disease prevalence between different human populations.

## Results

### Admixture mapping

**Figure 1:**
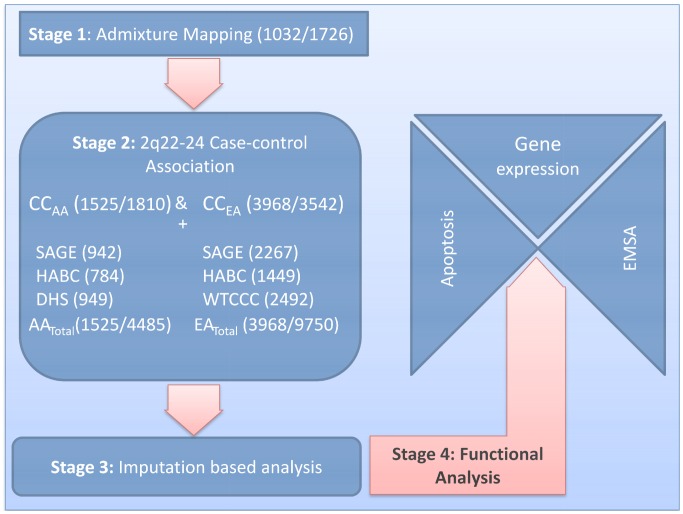
Study design. Our study design had four stages (sample sizes for cases/controls are in parentheses). In Stage 1, we performed admixture mapping of African Americans (AA) in a case-only analysis with 1032 cases and in a case-control analysis with 1726 controls. In Stage 2, the major admixture mapping signal at 2q22–24 was followed by a candidate gene analysis using case-control association in CC_AA_ (1525/1810) and European Americans (CC_EA_) (3968/3542), with 737 cases used for stages 1 and 2. In order to focus on our best candidate locus (*IFIH1*), we used out-of-study controls to increase control sample sizes to 4485 for AA and 9750 for EA. In Stage 3, we performed imputation based analysis on AA (1525/4485) and EA (3968/9750) to confirm our candidate gene analysis. In Stage 4, we performed functional analyses for the three confirmed SNPs. For the coding SNPs rs10930046 and rs1990760, we used an apoptosis assay to assess possible changes in protein function, and a gene expression assay to evaluate the effects of these SNPs on expression of genes related to apoptosis, inflammation and viral response. For the intronic variant rs13023380, we used EMSA to investigate whether the variant affected binding of the local DNA sequence to nuclear proteins.

**Figure 2:**
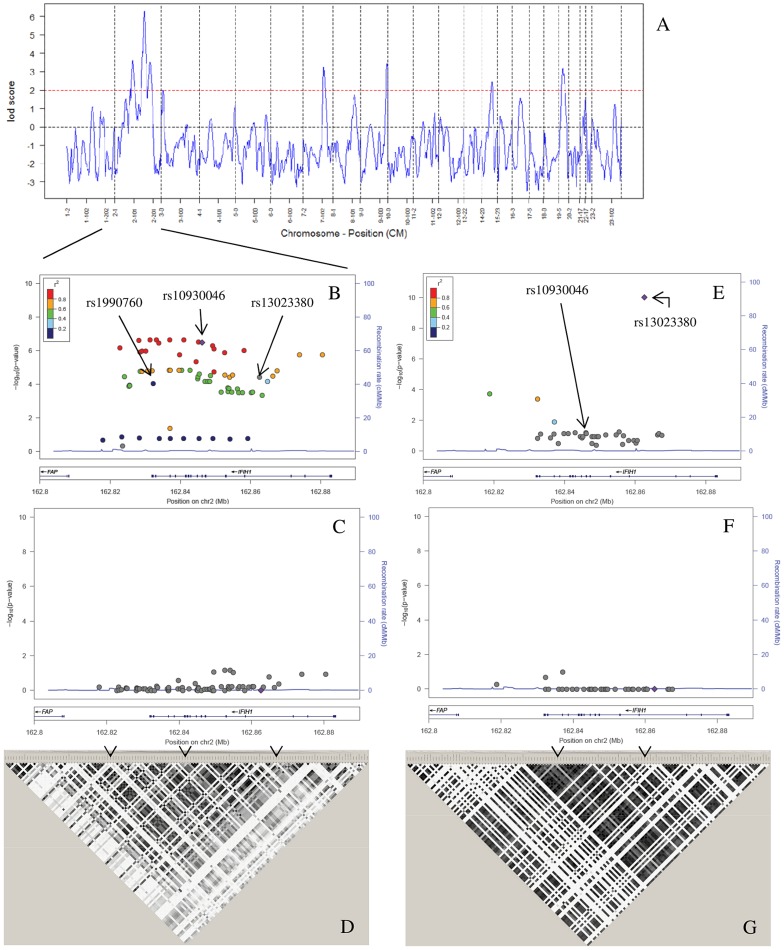
Admixture mapping and conditional analysis. (A) A whole-genome admixture scan on AA SLE cases identified 7 admixture signals that achieved the predefined LOD score >2 (red dashed line). (B) We performed an imputation-based association analysis of *IFIH1*, which was identified as the most promising candidate gene by a case-control study on 20 candidate genes in the largest peak (2q22–24), followed by 4-SNP haplotype conditional analysis (C). Filled dots indicate the −log_10_ P values for the association to SLE, and color coding represents inter-marker correlation (r^2^) between the strongest associated SNP, rs10930046 (“purple diamond”), and the individual SNPs, as shown in the color bar. (C) After conditioning the 4-marker haplotypes for the three markers rs1990760–rs10930046–rs13023380, all individual SNP associations are explained as shown. (D) We analyzed the LD between SNPs on the ImmunoChip, and these LD values were used as a reference panel for imputation in AA. Darker color denotes higher correlation between markers (r^2^). The LD pattern showed high correlation between markers, making it possible to increase SNP density by imputation. The three independently associated SNPs identified in (B) are denoted by arrows. (E) We performed an imputation based case-control association analysis in EA. Filled dots indicate the −log_10_ P values for each SNP, and color coding represents the inter-marker correlation (r^2^) between each individual SNP and the strongest associated SNP, rs13023380 (“purple diamond”), as shown in the color bar. (F) We then performed a two SNP haplotype analysis followed by a three marker haplotype analysis conditioned on the two independent variants rs10930046 and rs13023380. (G) LD analysis of SNPs on the ImmunoChip reference panel showed low inter-marker correlation, which largely precluded imputation based association. Darker color indicates greater r^2^. Arrows indicate the position of the independent SNPs.

Since case-only analysis has greater statistical power than case-control, we first performed a case-only admixture scan [3], [6], [8] on 1032 AA SLE cases (Figure 1, Table S1). Individual admixture estimates and genome scans for admixture mapping were analyzed using STRUCTURE [15] and ANCESTRYMAP [7] and later verified with ADMIXMAP [6]. As expected, a two-ancestral population model (African and European) best explained the population structure of these samples. By applying the ANCESTRYMAP software, we identified seven potential admixture signals that exceeded our predefined LOD threshold of 2 (Figure 2A, Table S2). Specifically, we identified a genomewide significant association [7] of SLE risk with European ancestry at 2q22–q24 (highest LOD = 6.28 was achieved between rs6733811 and rs4129786) using a weighted prior risk model; the strongest association at the same locus was observed at a fixed prior risk of 1.5, which represents a 1.5-fold increased risk of SLE due to one European ancestral allele at this locus. To evaluate how the prior risk model could influence the ANCESTRYMAP results, we also applied a uniform prior risk model and found consistent genomewide evidence for association at 2q22–q24 (LOD = 5.86). We also reassessed the strength of the admixture signal at 2q22–24 using alternate markers (LOD_odd_ = 3.65, LOD_even_ = 4.32), and computer simulation (P = 0.02). We also validated case-only admixture signals with a case-control admixture scan with 800 ancestry informative markers (AIMs), using 1726 controls from the Dallas Heart Study (DHS) (Table S2). We next repeated the admixture scan using ADMIXMAP, which uses a classical (non-Bayesian) hypothesis test (*i.e.*, score tests for allelic associations with the trait, conditional on individual admixture and other covariates). All the admixture signals identified by the case-only design using ANCESTRYMAP were strongly validated by ADMIXMAP (Table S2). The strongest peak was identified at 2q22–24 through ADMIXMAP (P = 2.99×10^−8^, Table S2). Both AM programs found that the strongest effect was on the AIM rs6733811 (P = 2.99×10^−8^, LOD = 5.65). Two weaker signals were also found on chromosome 2 (LOD = 3.61, P = 4.82×10^−3^; and LOD = 3.52, P = 1.64×10^−4^), as well as on chromosomes 7 (LOD = 3.26, P = 6.49×10^−6^), 9 (LOD = 3.43, P = 1.41×10^−5^), 14 (LOD = 2.44, P = 3.84×10^−5^) and 19 (LOD = 3.20, P = 1.15×10^−5^) (Table S2).

### Fine mapping and association analysis

To identify SLE-susceptibility gene(s) within 2q22–24, we performed a follow-up case-control (CC) association study in two ethnically diverse groups: CC_AA_ (1525 cases, 1810 controls) and CC_EA_ (3968 cases, 3542 controls) (Figure 1, Table S1). Individual ancestry was estimated using 216 highly informative AIMs. Case-control association tests were performed using 284 SNPs from 20 plausible candidate genes spanning ∼21 megabases of 2q22–q24 (95% CI (142.4–163.6), Table S3). Forty-two SNPs from 10 genes (*IFIH1*, *CACNB4*, *ACVR1C*, *KCNH7*, *NEB*, *STAM2*, *ZEB2*, *NMI*, *ARHGAP15*, and *ACVR2A*) showed significant association for the allelic test (P*_uncorrected_*<0.05) in CC_AA_, whereas 23 SNPs (in *IFIH1*, *CACNB4*, *NEB*, *ARHGAP15*, and *TNFA1P6*) showed significant association in CC_EA_ (Table S4). The strongest associations occurred at *IFIH1* (interferon-induced helicase 1; P_AA_ = 3.52×10^−5^, P_EA_ = 8.82×10^−5^) and *CACNB4* (voltage-gated calcium channel, beta subunit; P_AA_ = 9.07×10^−5^, P_EA_ = 2.61×10^−2^), which are separated by 10.2 Mb. Among the 22 SNPs tested within *IFIH1*, 13 were significantly (P<0.05) associated in AA and 4 in EA. Among 23 *CACNB4* SNPs, 12 were significant in AA and 2 in EA. Considering the number of associated SNPs, level of replication and involvement in autoimmune phenotypes [16]–[20], we considered *IFIH1* as the strongest candidate to explain the admixture-mapping signal.

Of the 13 SNPs significantly associated in AA, a preliminary imputation-based association analysis and comparing linkage disequilibrium (LD) determined that 11 were sufficient to tag the 13 associated SNPs (Table S6). To increase the statistical power to detect variants associated with SLE, we genotyped these 11 *IFIH1* SNPs in 949 healthy AA controls from the DHS, along with additional out-of-study controls (Figure 1, Table S1). Using single SNP analysis (allelic and genotypic models), followed by conditional analysis and LD analysis across two populations, we detected three SNPs with potentially independent SLE association (Table 1, Figure 2B and 2E). Based on an allelic model, intronic variant rs13023380 [P_AA_ = 4.33×10^−5^, P_EA_ = 9.52×10^−11^; P*_meta_* = 5.20×10^−14^; OR = 0.82 (0.78–0.87)], and a missense (Ala946Thr) variant rs1990760 [P_AA_ = 2.02×10^−4^, P_EA_ = 1.22×10^−4^; P*_meta_* = 3.08×10^−7^; OR = 0.88 (0.84–0.93)], were associated with SLE in both AA and EA. Another non-synonymous missense variant (Arg460His), rs10930046, was initially associated only with SLE in AA (P_AA_ = 1.81×10^−7^; OR = 0.80 (0.73–0.87)), where the best fit genetic model was identified as dominant (P*_dom_* = 1.16×10^−8^, OR = 0.70 (0.62–0.79)) (Table 1, Table S5). This SNP is rare in EA, having a minor allele frequency (MAF) of only 1.3% in controls and 1.6% in cases (P_EA_ = 0.086) (Table 1). However, after conditioning on the other two associated SNPs (rs13023380 and rs1990760), the rs10930046 became marginally significant in EA (P_EA_ = 0.017; OR = 1.2). These SNPs remained significant in both AA and EA after adjusting for ancestry (Table 1). Strikingly, the ancestral alleles at these three SNPs (all ‘G’) are the minor alleles in at least one population: all three ancestral alleles are the minor alleles in EA; the ancestral rs10930046 allele is minor in AA as well.

**Table 1:** Case-control association for genotyped variants in AA and EA.

SNP	Position	Alleles	AA				EA			
			Affected	Control	P-value	OR	Affected	Control	P-value	OR
			(N = 1525)	(N = 4485)		[95%CI]	(N = 3968)	(N = 9750)		[95%CI]
**rs13023380**	162,862,609	G/A	0.885	0.911	4.33×10^−5^ +	0.75	0.433	0.478	9.52×10^−11^ +	0.84
					3.92×10^−4^ *	(0.66–0.86)			6.66×10^−11^ *	(0.79–0.88)
					1.75×10^−2^ **				8.71×10^−11^ **	
**rs10930046**	162,846,229	G/A	0.374	0.428	1.81×10^−7^ +	0.80	0.016	0.013	8.61×10^−2^ +	1.21
					3.22×10^−6^ *	(0.73–0.87)			8.4×10^−2^ *	(0.97–1.50)
					8.51×10^−6^ **				0.12**	
**rs1990760**	162,832,297	G/A	0.799	0.829	2.02×10^−4^ +	0.82	0.375	0.400	1.22×10^−4^ +	0.90
					2.61×10^−3^ *	(0.74–0.91)			1.44×10^−4^ *	(0.85–0.95)
					1.43×10^−2^ **				1.74×10^−4^ **	

The numbers of affected and control samples are provided in parentheses. Allele frequencies, odds ratios (OR), and 95% confidence intervals [95% CI] are given for the ‘G’ alleles. Allelic association legends are:

+ uncorrected;

* corrected by local ancestry; and

** corrected by global ancestry.

We analytically estimated the joint population attributable risk (PAR) [21] using these three SNPs (rs13023380, rs10930046 and rs1990760) for AA (18.1%) and EA (14.7%). Most of the increased PAR (%) in AA was attributable to rs1090046 (12.5% PAR), whereas for EA very little was attributable to this SNP (0.3% PAR), likely due to the extremely low MAF. For AA, we also sought to determine how much of the European ancestry risk ratio (λ = 1.5, estimated by ANCESTRYMAP) was attributable to the three SNPs at 2q22–24. Using the estimated ORs in AAs and the SNP allele frequencies of the two ancestral populations (YRI, the Yoruba people of West Africa, was used as an African ancestral population; CEPH, Utah residents with Northern and Western European heritage, was used as an European ancestral population (Table S8), we estimated the locus-specific ancestry risk ratio (λ; see Methods) for each SNP (λ_rs1990760_ = 1.12, λ_rs10930046_ = 1.15, λ_rs13023380_ = 1.12). Assuming that each SNP contributes to the ancestry risk ratio independently, about 80% of the increased risk due to one copy of the European ancestry alleles estimated from ANCESTRYMAP (∼1.5) can be explained by the three SNPs at 2q22–24, reinforcing our conclusion from admixture mapping that local European ancestry increases the disease risk at 2q22–24.

We also repeated our admixture mapping by stratifying the cases by three genotype (‘AA’, ‘AG’ and ‘GG’) at the most differentiated (Fst_CEPH-YRI_ = 0.38) SNP, rs10930046 (N_AA_ = 279, N_AG_ = 323, N_GG_ = 114). Even with the small samples, we found a dramatically increased risk of European ancestry at rs10930046 (LOD = 10.78) in the homozygous ‘AA’ compared to the other genotypes, where ancestry association is insignificant (LOD for ‘GG’ = −6.46 and ‘AG’ = −3.38).

### Imputation-based association analysis

To identify additional SLE-associated variants, we performed an imputation-based association analysis in and around *IFIH1* using MACH [22] with reference data from AA (207 controls) and EA (594 controls) using genotyping data from the ImmunoChip (Figure 2B, 2E and Tables S6, S7). Using stringent predefined criteria for imputation, there were 61 additional SNPs for AA, but only 1 for EA later used for conditional analysis. Inefficiency of EA imputation was mainly due to presence of many low frequency (<1%) alleles and strikingly different LD structure (Figure 2D and 2G, Table S7).

### Conditional analysis

A pair-wise logistic regression analysis conditioned on each SNP revealed that the three previously identified SNPs were each independently associated with SLE. While in AA, rs13023380, rs10930046 and rs1990760 accounted for the entire association spanning the whole gene (Figure 2B, 2C), in EA, rs13023380 and rs10930046 were independently associated with SLE and accounted for the association (Figure 2E, 2F). Finally, comparing LD (r^2^) between these three SNPs across nine datasets from seven ethnic populations, we concluded that these three SNPs are also physically independent (Figure S2). Interestingly, using D′ we found that these SNPs are on the same haplotype in EA and AA, but most likely they are not in the ancestral populations (Figure S2).

### Ancestry and conservation of the associated alleles

In order to discover the ancestral origin of the risk (‘A’ in each of the 3 SNPs) and protective (‘G’ in each case) alleles for these three SNPs, we estimated local ancestry around the SNPs, then compared (by allele frequency and fixation index) AA individuals whose both haplotypes were European (AA_EUR_, N = 129) or African (AA_AFR_, N = 2124), and to individuals from HapMap populations CEPH and YRI (Table S8). Risk allele frequencies derived from the haplotypes were similar between AA_EUR_ and CEPH, and between AA_AFR_ and YRI. Alignment of the human genome with other genomes strongly suggests that the protective alleles (‘G’) are ancestral, and that the risk (‘A’) alleles are derived. For the two coding SNPs, the ‘G’ allele of rs1990760 (and the resulting alanine amino acid) is ∼100% conserved across 34 mammalian genomes (Table S9); the ‘G’ allele of rs10930046 (and the resulting arginine amino acid) is ∼100% conserved across 50 vertebrate genomes (Table S10). Introns are typically less conserved than protein-coding sequence, and accordingly the intronic sequence surrounding the rs13023380 SNP is only strongly conserved in primates; the base corresponding to rs13023380 is ‘G’ in each case (Figure S6). In AA, only the rs10930046 risk allele is major; interestingly, all three ‘A’ risk, derived alleles are the major alleles in EA and the rs10930046 risk allele is almost fixed (Table 1). This suggests a strong selective pressure against the SLE-protective alleles in humans [23], which is not manifest in other animal species.

### Experimental validation

**Figure 5:**
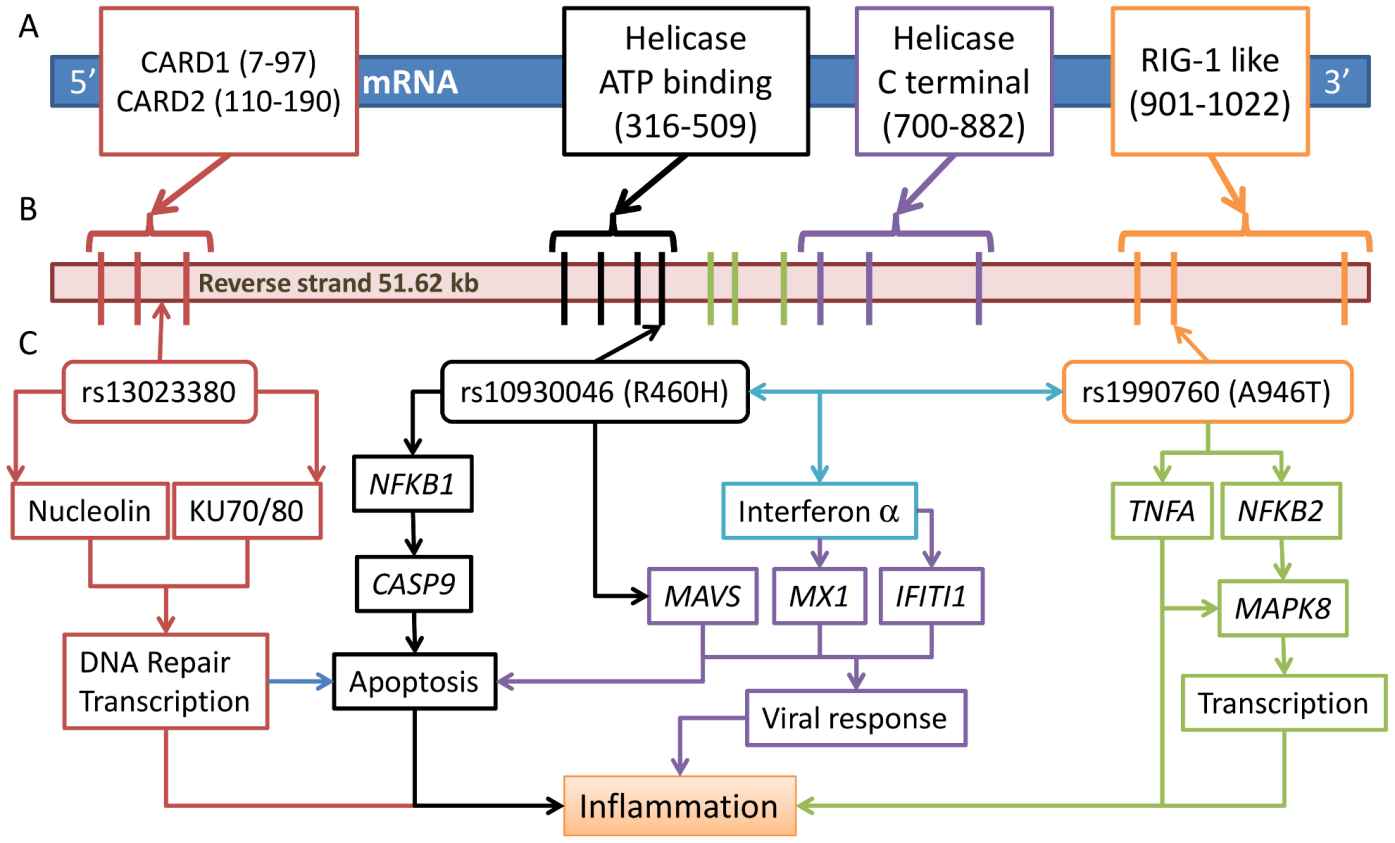
Three risk variants of *IFIH1* and the proposed functional model. (A) *IFIH1* protein (1025 amino acids (aa)) structure shows four conserved domains (start-stop aa) in boxes: a caspase recruitment (CARD) domain 115–200 aa); a helicase ATP-binding domain (305–493 aa), a helicase C-terminal domain (743–826 aa), and a RIG-I regulatory domain (901–1022 aa). (B) Domain prediction by Pfam: Gene intron/exon structure is presented below, with exons represented by thick vertical lines. (C) The three associated SNPs are represented by arrowed boxes. SNP rs10930046 is located in the helicase ATP-binding domain encoded by exons 4–7; rs1990760 is located in the RIG-1 regulatory domain encoded by exons 14–16; rs13023380 is on intron 3. We present a model of the functions implicated by the risk alleles in each variant, where each identified variant has an effect on the expression of *NFκ-B1*, *CASP9*, *MAVS*, *MX1*, *IFIT1*, *NFκ-B2*, *TNFA* and *MAPK8*, and their impact on inflammation, viral response and transcription.

Given the strong association of these three SNPs in *IFIH1* with SLE, we evaluated their effect on the function of the *IFIH1* gene. *IFIH1* has been implicated in binding with dsRNA complexes generated as replication intermediates during RNA viral infections, leading to inflammation and apoptosis [24], [25]. The full length *IFIH1* protein contains 1025aa in the following domains: caspase recruitment (CARD) (aa115–200), helicase ATP-binding (aa305–493), helicase C-terminal (aa743–826) and RIG-I regulatory (aa901–1022) (Figure 5A). Deletion of the ATP-binding domain, which includes rs10930046, induces apoptosis in melanoma cells [26]. The RIG-I regulatory domain, which includes rs1990760, recognizes dsRNA, upon which the helicase domains are activated [27]. Apoptosis has been associated with SLE pathogenesis in humans and mice [28]. Furthermore, Ingenuity Pathway Analysis (IPA) indicates that *IFIH1* interacts with several genes involved in apoptosis and inflammation (Figure S3).

### Apoptosis

**Figure 3:**
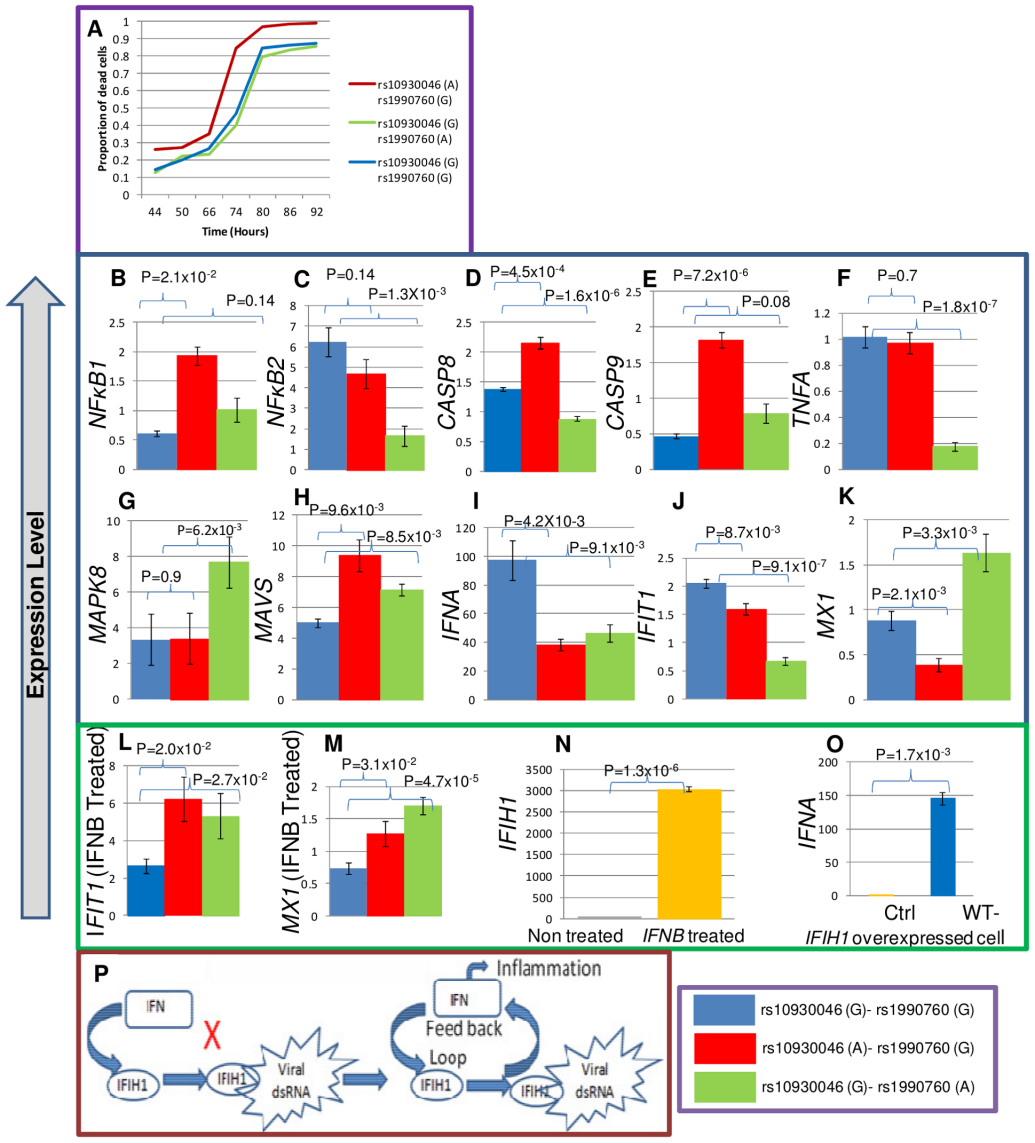
Apoptosis and expression assays for exonic SNPs. (A) K652 cells were transfected with *IFIH1* full length cDNA containing the protective ‘G’ or risk ‘A’ allele for rs10930046 and rs1990760 in the following combinations: ‘A-G’, ‘G-A’ and ‘G-G’. After transfection, the percentage of apoptotic cells was quantified by FACS for annexin V and AAD positivity among GFP+ cells (transfection positive) at seven different time points (in hours). At all time points, the risk allele ‘A’ for rs10930046 produced a significant increase in the proportion of apoptotic cells compared to the ‘G’ allele (mean increase 14.6%, P≤1.4×10^−3^, ‘A-G’ vs ‘G-G’). In contrast, the ‘A’ allele for rs1990760 had no significant effect on apoptosis compared to the ‘G’ allele at any time point (P = 1, ‘G-A’ vs ‘G-G’). *IFIH1* cDNA encoding the risk or protective allele of rs10930046 or rs1990760 was transiently transfected, GFP+ cells were sorted by FACS and total RNA was isolated. cDNAs were subjected to RT-qPCR for quantification of expression of *NFκ-B1*, *NFκ-B2*, *CASP8*, *CASP9*, *TNFA*, *MAPK8*, *MAVS*, *IFNA*, *IFIT1* and *MXI* (B–K). All of these genes showed significantly altered expression with at least one of the risk alleles. Further, we observed that *IFIT1* and *MX1* (L, M) expression both increased with risk alleles when transfected cells were treated with IFN beta. Also, IFN beta stimulation increased *IFIH1* expression (N), and *IFIH1* over-expression induced *IFNA* expression (O), suggesting a positive feedback loop between *IFNA* and *IFIH1* (P). Although it was known that *IFNA* induces *IFIH1* expression, it was not previously shown that *IFIH1* induces *IFNA* expression, which here is designated by “X”. Colors used in the expression figures are blue for ancestral ‘G-G’, red for rs10930046-‘A’+rs1990760-‘G’; and green shows rs10930046-‘G’+rs1990760-‘A’.

To assess the effects of coding variants in apoptosis and inflammation, we mutagenized *IFIH1* cDNA cloned in a mammalian expression vector with a poly-cistronic (IRES) GFP marker at the C-terminus. We over-expressed *IFIH1* in a K562 leukemia cell line and measured cell death for each risk SNP, comparing with the ancestral protective allele. The rs10930046 risk allele ‘A’ significantly increased apoptosis over the protective allele ‘G’ (14.6% average increase at each time point between 44 and 92 hours, P = 0.014) (Figure 3A). In contrast, the risk allele ‘A’ of rs1990760 had little impact on apoptosis (P = 1.0), as expected since it is located in the RIG-1 regulatory domain, which is not involved in apoptosis.

### Gene expression

To assess the effect of these polymorphisms on expression of downstream genes, additional transfected K562 cells were sorted for GFP+ cells by FACS and total RNAs were isolated from these cells. These were subjected to RT-qPCR of 11 genes related to apoptosis, inflammation or viral response: *NFκ-B1*, *NFκ-B2*, *RELA*, *CASP8*, *CASP9*, *TNFα*, *MAPK8*, *MAVS*, *IFNA*, *IFIT1* and *MX1*.

Gene expression analysis showed that over-expression of the ‘A’ allele of rs10930046 significantly increased expression of *NFκ-B1* (>2.8-fold, P = 2.1×10^−2^), *CASP8* (>1.8-fold, P = 4.5×10^−4^), *CASP9* (>3.5-fold, P = 7.2×10^−6^) and *MAVS* (>2-fold, P = 9.6×10^−3^) compared to the ‘G’ allele (Figure 3B, 3D, 3E, 3H) but did not affect expression of *NFκ-B2* (P = 0.14), *TNFα* (P = 0.7) or *MAPK8* (P = 0.9) (Figure 3C, 3F, 3G). While the ‘A’ allele of rs1990760 had no significant effect on expression of *NFκ-B1* (P = 0.14) or *CASP9* (P = 0.08), it showed a significant decrease of *TNFα* (>5-fold, P = 1.8×10^−7^), *NFκ-B2* (>3-fold, P = 1.3×10^−3^) and *CASP8* (>1.5-fold, P = 1.6×10^−6^) expression (Figure 3B, 3C, 3D, 3E, 3F). The rs1990760 risk allele also significantly increased expression of *MAPK8* (>2-fold, P = 6.2×10^−3^) and *MAVS* (>1.6-fold, P = 8.5×10^−3^) (Figure 3G, 3H).

Strikingly, interferon alpha (*IFNA*) expression was reduced for both risk alleles (rs10930046, >2-fold, P = 4.2×10^−3^; rs1990760, 2-fold, P = 9.1×10^−3^) (Figure 3I). Reduced *IFNA* expression in SLE patients had been predicted for the risk allele of rs1990760 [29]. Our results not only confirmed this but also showed that expression of the risk allele of rs10930046 similarly reduced *IFNA* expression (Figure 3I). Similarly, expression of *IFIT1* was also reduced (rs10930046, >0.75-fold, P = 8.7×10^−3^; rs1990760, >3.5-fold, P = 9.1×10^−7^); and *MX1* expression was decreased for rs10930046 (>2.5-fold, P = 2.1×10^−3^) but was increased for rs1990760 (>1.5-fold, P = 3.3×10^−4^) (Figure 3J, 3K). Following induction of transfected cells with Type-1 interferon IFN beta (*IFNB*), *IFIT1* and *MX1* showed strong up-regulation (Figure 3L, 3M) by both SNPs (*IFIT1*: rs10930046, >2-fold, P = 2.0×10^−2^; rs1990760, >2-fold, P = 2.7×10^−2^; *MX1*: rs10930046, >1.3-fold, P = 3.1×10^−2^; rs1990760, >2.2-fold, P = 4.7×10^−5^). *RELA* expression did not change significantly (for rs10930046, P = 0.61 and for rs1990760, P = 0.77) for either risk allele (not shown).

In our expression analysis, significant up-regulation of *CASP8*, *CASP9* and *NFκ-B1* (and unchanged *NFκ-B2* and *TNFα* levels) by the rs10930046 risk allele would be expected to dramatically increase apoptosis, as observed. For rs1990760, levels of these five pro-apoptotic factors are dramatically lowered, consistent with absence of an apoptosis phenotype. *MAVS* (mitochondrial antiviral-signaling protein) expression was increased for both risk alleles. *MAVS* is an antiviral protein in the host defense system whose virus-triggered cleavage is necessary to attenuate apoptosis [30], [31]. However, without viral attack *MAVS* induces apoptosis through caspase and *NFKB* activation [30]. In our case, it could promote apoptosis, particularly for the risk allele of rs10930046. In terms of inflammation, the expression data shows some interesting effects. For rs10930046, neither *TNFα* nor *MAPK8* changed, but for rs1990760, *TNFα* decreased while *MAPK8* increased leading to inflammation signaling through non-apoptotic pathways.

We next examined known transcriptional networks in the context of our expression data. At the root, *IFIH1* and type-1 interferons constitute a positive-feedback loop (Figure 3P). We verified this in our cellular model: indeed, in control cells, *IFIH1* expression increased several hundred-fold upon *IFNB* treatment (Figure 3N), and in *IFIH1* over-expressing cells, *IFNA* expression increased (Figure 3O). Taken together, our results support the predicted *IFIH1*-Type1 interferon feedback loop through *IRF7* [32] and *MAVS* [33].

*IFNA* and *TNFα* are known to drive *IFIT1* expression [34], and the *IFIH1* SNP-driven decrease in *IFIT1* may be mediated through decreased *IFNA* and/or *TNFα*. *MX1* (interferon-induced GTP-binding protein) is also driven by *IFNA* and *TNFα* [35]. Surprisingly, although both *IFNA* and *TNFα* decreased in the presence of rs1990760 ‘A’, *MX1* was significantly up-regulated. This result is similar to a recent paper [29], which showed that when SLE patients' cells were induced with *IFNA*, the rs1990760 ‘A’ risk allele displayed higher levels of *MX1* than ‘G’ allele patients, even though these patients had lower circulating IFNA levels. Our data suggest that *IFIH1* risk alleles at these two coding SNPs down-regulate *IFNA* expression (either through reduced expression or activity of *IFIH1*) and, in turn, *IFNA* down-regulates interferon regulatory antiviral genes, potentially conferring viral susceptibility. It is also possible that the rs1990760 risk variant in *IFIH1* may increase sensitivity of cells to IFNA pathway activation and subsequent IFN-induced gene transcription [36].

### Protein binding, identification, and *in vitro* binding

**Figure 4:**
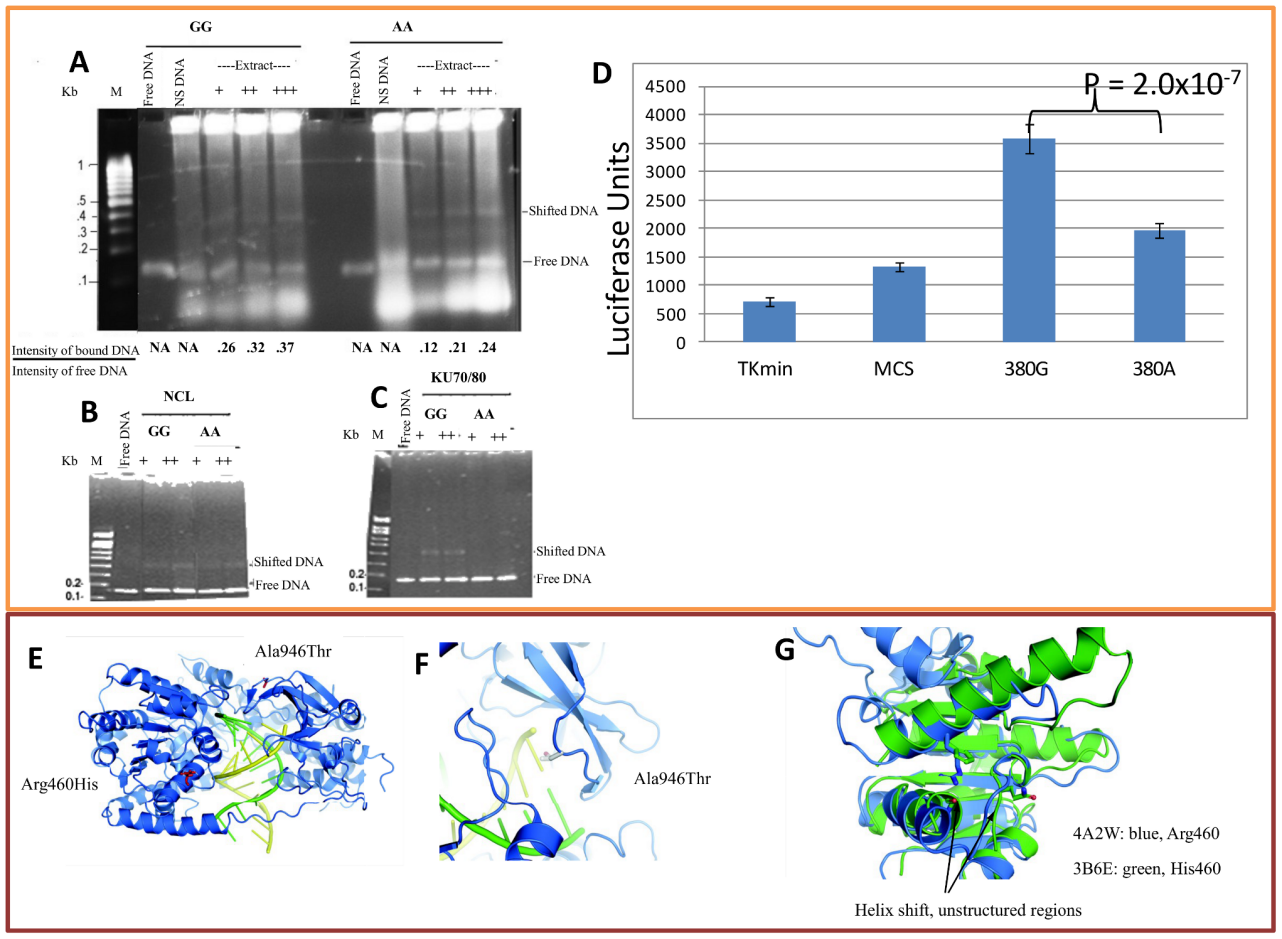
Binding assay for rs13023380 and molecular model of *IFIH1*. (A) EMSA was performed using nuclear protein extracts from K562 cells (A) with 141-bp PCR products including either the protective (‘G’) or risk (‘A’) sequence at rs13023380. Both ‘G’ and ‘A’ allele-containing PCR products bound to a protein complex in the nuclear extracts. However, the ‘A’ allele bound with at least 2-fold reduced efficiency compared to the ‘G’ allele-carrying PCR product, as measured by the intensity of the shifted band relative to the free DNA band in the same lane. As a nonspecific (NS) DNA control, a 140-bp DNA sequence not present in the genome was created by PCR amplification of bisulfite-modified genomic DNA. (B, C) EMSA for purified recombinant Nucleolin and Ku70/80 protein with PCR products carrying the ‘G’ or ‘A’ allele of rs13023380. In both the cases, the ‘G’ allele binds both of these proteins with increased efficiency. +signs are used to denote the increasing amount of protein added in the reaction. Numbers below EMSA pictures denote the ratio between the intensities of protein bound DNA to the free DNA. (D) Luciferase activities of intronic DNA sequences carrying ancestral ‘G’ or risk allele ‘A’. The protective allele has approximately 2-fold higher promoter activity (luciferase units) than risk allele ‘A’ carrying sequences. *Tkmin*-only vector, MCS-vector with multiple cloning sites, 380G-protective allele, 380A-risk allele. (E) Crystal structure of RIG-I in complex with dsRNA (from PDB 3TMI) [27]. Side-chains are shown in red for the positions corresponding to the two coding SNPs in *IFIH1*. Both mutations are in close proximity to the dsRNA-binding pocket. (F) Close-up of the side-chain of Ala946, modeled from 3TMI. The side-chain makes close contact with the opposing helicase “cap” domain; together these two domains regulate dsRNA entry and processing. Threonine is shown in transparent colors. (G) Superimposition of the RIG-I ATP-binding domain (PDB 4A2W) in blue, and the human *IFIH1* ATP-binding domain (PDB 3B6E) in green. The *IFIH1* structure contains the histidine side-chain resulting from the rs10930046 risk allele. Large portions of the *IFIH1* structure are absent in the 3B6E model, and the two helices are shifted by 1.5 Å. In the ancestral protein, Arg460 likely interacts with the Leu421 main-chain oxygen, as well as the negative helix dipole and the side-chains of Gln433, and Glu425 and 428 (not present in 3B6E).

The intronic variant rs13023380 could influence *IFIH1* function either by producing a functional miRNA or by altering the binding efficiency to one or more nuclear regulatory proteins. Through bioinformatic analyses (miRBase: http://www.mirbase.org/) we confirmed that no reported or predicted miRNA-producing or binding sites were present in these sequences. To address whether rs13023380 alters nuclear protein-DNA interaction, we performed EMSAs on nuclear protein extracts from K562 and JURKAT cell lines, using 150-bp PCR products amplified from genomic DNA of ‘AA’ and ‘GG’ homozygous patients. Both PCR products containing the ‘A’ risk sequences or ancestral ‘G’ sequences bound to nuclear protein extract, but DNA containing ‘A’ sequences consistently showed ∼2-fold reduced binding efficiency to a protein complex compared to ‘G’ sequences (Figure 4A, Figure S4F).

To identify any DNA-bound proteins, we performed mass spectrometric sequencing (MALDI-TOF) on the protein/DNA complexes isolated using two separate methods: 2D electrophoresis and protein pull-down. In 2D electrophoresis, the visible DNA-bound protein complex in EMSA was excised from a native PAGE gel (Figure S4A–S4C) and sequenced directly, which identified lupus autoantigen Ku70/80 (*XRCC5/6*), nucleolin (*NCL*) and HSP90AA1/AB1 as the major constituents of the DNA–protein band (Table S11). Using the second method, we performed EMSA with biotinylated PCR products and pulled down the DNA-bound proteins using immobilized streptavidin-coated agarose beads. Subsequent fractionation by SDS-PAGE (Figure S4D), and sequencing of two distinct visible protein bands (not present in the control pull-down product), confirmed NCL and HSP90AB1 (Table S11). We did not identify Ku70/80 in the streptavidin method, possibly because these two proteins were washed off or were present in insufficient quantities to detect and sequence. However, when we performed “super-shift” assays with antibodies to these proteins, surprisingly, anti-NCL and anti-Ku70/80 antibodies released EMSA-bound DNA instead of super-shifting the complex (Figure S4E). It is possible that the antibodies either induce conformational changes in their targets to release DNA or compete with target proteins for DNA binding. Autoantibodies against NCL and Ku70/Ku80 are characteristic features of SLE [37], [38] and release of free DNA from EMSA-bound DNA *in vitro* implies that autoantibodies *in vivo* could impair the function of these proteins by disrupting the binding of bound proteins from target DNA, including the rs13023380 locus.

In light of the observed competition of added antibodies to protein-DNA binding, we determined whether purified recombinant proteins of NCL and Ku70/80 bound to these DNAs. Both recombinant proteins, purified from insect cells, produced identical gel shifts as the nuclear extract (Figure 4B, 4C), but again the risk ‘A’ allele bound to the recombinant proteins with ∼2-fold decreased efficiency relative to the protective ‘G’ allele.

These results prompted us to enquire whether DNA sequence containing rs13023380 and its surroundings could act as a transcriptional regulatory element (TRE, *e.g.* enhancer/silencer) *in vivo*, and if the risk allele has any effect on transcription. The same sequences used for EMSA were cloned before a minimal *TKmin* promoter and a luciferase reporter gene, and luminescence assays were performed. Both sequences increased reporter gene activity over the core vector, suggesting that the rs13023380 locus contains a transcriptional enhancer. The risk allele-carrying sequences showed almost a 2-fold reduction (Figure 4D) in luciferase activity compared to those with the ancestral allele. Taken together, these results suggest that the rs13023380 locus recruits transcriptional activity of *IFIH1* through binding of Ku70/80, NCL and HSP90AA1/AB1 (and potentially more proteins), and that the risk allele at this base position interferes with this enhancer activity, potentially decreasing *IFIH1* transcript levels.

### Molecular modeling

The absolute conservation of Ala946 (rs1990760, Ala946Thr) in all sequenced mammalian genomes, with diverse codons, strongly suggests selection at the amino acid level (Table S9). We performed molecular modeling of *IFIH1*-Thr946, based on the protein structure of the *IFIH1* C-terminal domain (PDB 2RQB), and the full-length structure of the homologous enzyme RIG-I, bound to dsRNA (PDB 3TMI). Ala946 is placed directly at the mouth of the helicase active site; in RIG-I this region makes contact with the helicase “cap”, which mediates dsRNA entry and processing [27]. Mutation of alanine to the bulker threonine side-chain (Figure 4E, 4F) may alter the sterics and/or dynamics of this protein region, leading to loss-of-function.

Similarly, Arg460 (rs10930046, Arg460His) is conserved in all vertebrate genomes sequenced, with diverse codons, again implying amino acid-level selection (Table S10). Comparison with RIG-I (PDB 3TMI) suggests that in the ancestral protein, Arg460 may form hydrogen bonds with the 419–433 loop, most likely with the strictly conserved acidic side-chains of Glu425 and Glu428, and the conserved Gln433 (Figure 4E, 4G). Intriguingly, the crystal structure of the human *IFIH1* ATP-binding (DECH) domain (PDB 3B6E) incorporates the pervasive rs10930046 risk mutation. In this structure the His460 side-chain does not make favorable contacts with the 419–433 loop and much of this loop is poorly structured. Loss of stabilizing interactions of Arg460 might lead to weakened structural integrity of the helicase ATP-binding domain (the 3B6E domain is internally shifted ∼1.5 Å relative to the RIG-I structure; Figure 4G), and subsequently with the helicase C-terminal and RIG-I regulatory domains. DsRNA binding, which occurs at a site proximal to the rs10930046 mutation (Figure 5A), leads to RIG-I dimerization [39]. The disruptive nature of the rs10930046 risk allele on overall protein structural integrity apparently decreases dimerization, as the 3B6E structure was determined as a monomer (all related structures are dimers). Indeed it is likely that the rs10930046 risk allele structure “poisons” an ancestral binding partner, leading to a dominant negative phenotype, consistent with the genetically dominant model, especially in AA.

The intronic rs13023380 risk allele has no effect on the protein-coding sequence of *IFIH1*. The region directly surrounding rs13023380 is rich in strongly conserved C/G bases (Figure S6A). Given the binding of the locus to NCL and other nuclear regulatory proteins, we hypothesized that the site might play a role in mRNA processing. Modeling of the region around rs13023380 predicts a highly structured pre-mRNA, with strongly favorable folding free energies (CentroidFold, ncRNA.org) (Figure S6B). In the ancestral pre-mRNA, the rs13023380 base is part of a highly structured 7-mer RNA stem with a 7-base loop (Figure S6B). In the risk allele pre-mRNA, mutation of the conserved rs13023380 base disrupts RNA stem formation, and likely perturbs structure and stability of the loop (Figure S6C, S6D), which might disrupt the binding of RNA-binding proteins (such as NCL [40]), impairing pre-mRNA trafficking and processing.

## Discussion

Our whole genome admixture scan identified 7 admixture peaks associated with SLE in AA, with the strongest at 2q22–24, containing the *IFIH1* gene. Three SNPs (two coding: rs1990760 and rs10930046, and one intronic: rs13023380) accounted for the increased risk. *IFIH1* has been associated with Type 1 diabetes (T1D) [41], IgA deficiency [18], Graves' disease [17], and suggestively linked to SLE [20], [42]. The role of *IFIH1* in apoptosis and inflammation makes it potentially critical for SLE progression. Moreover, allele frequency differences in associated and non-associated SNPs (high F_ST_ values), together with the differences in the number of rare variants between EA and AA, imply a strong positive selection in EA (intriguingly, for the SLE-risk alleles at all three positions), as previously suggested [23]. In AA, local European ancestry at these loci correlates with increased risk.

Variant rs1990760 has been recently reported to affect expression of viral resistance genes *IFIT1* and *MX1* in SLE patients [29]. The risk allele of rs1990760 positively correlated with interferon-induced gene expression in SLE patients who were positive for anti-dsDNA antibodies [29]. Another report on rs1990760 suggested that the risk allele correlated with increased expression of *IFIH1* in T1D patients [32]. The rs10930046 risk allele has been implicated in psoriasis susceptibility [43]. Here we have systematically examined the effects of the two coding SNPs on immune cell biology, and demonstrated that the rs10930046 risk allele dramatically increases apoptosis, and that both significantly perturb inflammatory gene profiles. The intronic risk allele disrupts a transcriptional enhancer that recruits nucleolin, lupus autoantigen Ku70/80 and HSP90, potentially decreasing IFIH1 transcript levels. Combined with molecular modeling, our results strongly suggest that these effects are due to several specific amino acid and nucleotide substitutions, rather than to indirect effects due to LD with other SNPs.

SLE is commonly identified with an up-regulation of the interferon pathway [44]. Intriguingly, our results suggest that the two non-synonymous *IFIH1* mutations down-regulate interferon signaling. However, recent findings demonstrated that SLE patients with anti-DNA antibodies have lower serum *IFNA* levels [29], and this dose-dependent decrease suggests that there exists a sub-population of SLE patients with lower serum *IFNA* levels with increased IFN sensitivity [36]. Heterogeneity is also observed in clinical *TNFα* levels; rs1990760 would seem a likely candidate to be associated with low *TNFα* levels in this patient sub-population [45].

The intronic SNP (rs13023380) discovered in this study has not been previously implicated in SLE or any other medical condition. The transcriptional enhancer uncovered in this genomic region, and the risk allele's disruption of its activity, opens up new avenues for investigation. Nucleolin, in addition to contributing to RNA polymerase 1 function [46], is known to be a principal component of the B-cell transcription factor complex LR1 [47], which binds the Ig heavy chain switch region and functions in Ig recombination. Disruption of nucleolin binding to the rs13023380 risk allele may dysregulate polymerase binding, *IFIH1* transcription, autoantibody production and interaction. The region surrounding rs13023380 is rich in highly conserved C/G bases, which are preferentially recognized by NCL and Ku70/80 [48], [49]. In addition to perturbing transcription at the locus, molecular modeling implicates the base substitution in destabilizing non-spliced mRNA, further altering proper regulation of expression levels. Nucleolin, as a matrix-binding protein [50], could also provide a scaffold for matrix and DNA during immunoglobulin hyper-recombination. Thus in SLE patients, a positive feedback loop potentially exists where genetic susceptibility creates a biochemical imbalance, dysregulating NCL, which may then promote antibody hypermutation and autoantibody production, further destabilizing the cellular network. Similarly, Ku70/80 facilitates DNA repair and promotes transcription initiation by complexing with RNA polymerase 1. Disruption of Ku70/80 binding to the rs13023380 locus would be expected to have similar consequences for autoantibody production and interaction. Additionally, Ku70/80 mediates the predominant pathway of non-homologous end joining (NHEJ) during immunoglobulin class switch recombination (CSR) [51], [52]. Our discovery that antibodies directed against NCL and Ku70/80 promote release of dsDNA by nuclear proteins suggests that in SLE, hallmark autoantibodies against these two proteins may alter their activity. Thus in SLE patients, genetic susceptibility could create a biochemical imbalance that dysregulates NCL, Ku70/80, or other nucleic acid regulatory proteins binding to the rs13023380 locus (and other DNA sequences). Follow-up studies could systematically explore the effect of both antibodies and SNP-induced protein mutations on the DNA-binding and transcriptional properties of a number of gene products implicated in SLE and other diseases.

From an evolutionary point of view, evidence suggests that *IFIH1* is under strong positive selection, especially in EA. The derived allele of rs10930046 (risk in AA) is highly differentiated in ethnically diverse populations and allele frequency increases from AA (60%) to EA (>98%), and may be acting as a protective allele (for some condition other than SLE). This indicates that at some time point, the risk allele may have offered competitive advantage to individuals by increasing apoptosis in defense to new threats of infection encountered during migrations to the New World from Africa [53]. This selection is evident in an observed gradient of geographical distribution (Figure S5) of the allele frequency [53]. Therefore, the different haplotype block structure between the two groups derived from African and European homozygotes is expected.

To evaluate the epidemiological significance of *IFIH1* polymorphism in the genetic and ethnic background of SLE in AA and EA populations, we estimated the joint PAR. The joint PAR from the three SNPs for AA and EA are 18.1% and 14.7%, respectively. Most of the increased PAR in AA was attributable to rs1090046 (12.5%). Interestingly, the admixture peak at 2q22–24 is associated with increased local European ancestry, suggesting that European ancestry at this locus confers a higher risk of SLE compared to African ancestry. Indeed, we observed the strongest locus-specific LOD score at 2q22–24 using a fixed prior risk of 1.5, meaning that carrying one European ancestry allele confers 1.5 fold increased risk of SLE relative to having no European ancestry alleles. Furthermore, we addressed the question whether the locus-specific ancestry risk ratio calculated from the estimated OR of the three SNPs in AA and their allele frequencies in ancestral populations accounts for the European ancestry risk ratio of ∼1.5 estimated from the admixture scan. The total increased ancestry risk (45%) due to these three SNPs (λ_rs1990760_ = 1.12, λ_rs10930046_ = 1.12, λ_rs13023380_ = 1.16) were close to the increased risk 50% estimated from our admixture mapping (λ = 1.5). Therefore, the locus-specific ancestry risk ratio corroborates the ancestry risk ratio estimated from the admixture scan.

In summary, to our knowledge this is the first study to use a whole-genome admixture mapping design to identify SLE susceptibility loci, confirm case-control association analysis in AA and EA, and identify novel variants within *IFIH1* associated with SLE susceptibility. We report three independently associated *IFIH1* variants with significant ethnic variation, providing a possible basis for differences in SLE risk between ethnically diverse populations. In addition, we show allele-specific differential cellular signaling and predict an *in vivo* role of Ku70/80 and NCL autoantibodies that could impair function of *IFIH1* by disrupting DNA binding. Therefore, these results clearly establish *IFIH1* as an SLE susceptibility gene and provide mechanisms for the *IFIH1* variants in SLE etiopathogenesis.

## Materials and Methods

### Study design

We designed our study in four stages: In *Stage 1*, we performed a case-only admixture scan on 1032 AA SLE cases (Figure 1, Table S1) followed by exploring the largest admixture peak at 2q22–24 with a more focused candidate gene analysis in both AA and EA populations (*Stage 2*), including out-of-study controls from dbGaP for AA (2675) and EA (6208) to boost statistical power. In *Stage 3*, an imputation-based analysis was performed to fine-map our selected gene using SNPs from ImmunoChip arrays [54]. In *Stage 4*, we experimentally tested the biochemical function of associated SNPs in cellular models, *in vitro* protein experiments and molecular modeling.

### Demographics

For our admixture mapping (AM), we used 1032 African-American (AA) SLE cases and 1726 AA controls (Table S1). Individuals were recruited by the coordinating institutions: Lupus Family Repository and Registry (LFRR) at the Oklahoma Medical Research Foundation (OMRF, 540 AA), and the University of Alabama at Birmingham (UAB) (492 AA) through the PROFILE study group. Cases fulfilled at least 4 of 11 criteria from the American College of Rheumatology (ACR) [55], [56] based on medical record review.

In the follow-up case control study (CC), we used both AA (CC_AA_: 1525 cases and 1810 controls,) and European-Americans (EA) (CC_EA_: 3968 cases and 3542 controls). There was an overlap of 737 cases between AM and CC. We increased the sample size using out of study controls from publicly available datasets in dbGaP [57] (dbGaP see Web Resources), including 942 AA and 2267 EA controls from the Study of Addiction (SAGE); 784 AA and 1449 EA controls from Health ABC (HABC); 2492 EA controls from the Wellcome Trust Consortium (WTCCC see Web Resources); 949 AA controls from the Dallas Heart Study (DHS see Web Resources) provided by Dr. Helen H. Hobbs. AA and EA samples were collected by the coordinating institutions: LFRR, BIOLUPUS, Medical University of South Carolina, the PROFILE study group, the Oklahoma Lupus cohort, the Feinstein Institute of Medical Research and ODRCC.

All individuals were de-identified prior to being genotyped. Within each stage of this experiment, all cases and controls were independent. This study was approved by the Institutional Review Boards of the OMRF or the ethical committees at the institutions where subjects were recruited.

### Genotyping and quality control

Rigorous quality control (QC) was applied to all data used in this study. Subjects were excluded from analysis if they had <95% genotyping success or were population-stratification outliers. Using ancestral informative markers (AIMs), we performed principal components analysis (PCA) using EIGENSOFT [58] (EIGENSOFT see Web Resources) and STRUCTURE [59] (STRUCTURE see Web Resources) to identify outliers, hidden population structure and estimate individual ancestry proportions (European). Relatedness between individuals was calculated using PLINK [60] (PLINK see Web Resources) and GCTA [61]. All related and duplicate individuals (r>0.25) were removed. SNPs were removed for >10% missing genotyping, being out of Hardy-Weinberg equilibrium (HWE, P<0.001 in controls) or for poor clustering. SNPs were also removed for minor allele frequency (MAF) <1%. We used AIMs which passed QC, had a minimum intermarker distance >1 Megabase and were not in linkage disequilibrium (LD) in the ancestral populations. In addition to these QC measures, imputed SNPs were included in the analysis only if Rsq >0.90. This ensures the all high quality imputed SNPs were included in the analysis.

Genomic DNA samples in AM from SLE patients were genotyped at OMRF using Affymetrix MALD 3K panel, including 2154 SNPs that passed QC. Fourteen duplicate individuals were removed. DHS samples were genotyped by Perlegen Sciences including 800 AIMs and 10 SNPs within 2q22–24. Samples in the follow-up CC study were genotyped on a custom Illumina iSelect platform. SAGE, HABC and WTCCC genotypes were merged with our data for further QC. PCA was performed for all samples using 401 SNPs to detect PCA outliers from SAGE, HABC and WTCCC. We used 163 AIMs to compare our samples with SAGE, and 755 AIMs to compare our samples with DHS (Figure S1). After QC, individuals were removed from analysis if they were related or duplicate (57 AA and 138 EA), had a missing genotype call rate >5% (19 AA and 361 EA), or were within 2 standard deviations of the mean first eigenvector (30 AA and 51 EA) (Figure S1). Since we used several thousand out of study controls from different publicly available sources [dbGaP (SAGE, HABC) and WTCCC], we had to take very strict QC. Some from the out of study control samples were identified as outliers by PCA; these individuals were mainly the AA samples with >75% European ancestry, and EA samples with <90% European ancestry. All SNPs that passed QC were used in the analysis.

### Stage 1: Admixture mapping

#### SNP panel used for admixture mapping

Our initial marker set was taken from the Smith panel [62], which has been validated as having little LD in the parental African and European populations. To optimize these SNPs for our AM study, 1533 AIMs were selected for case-only analysis. We used ANCESTRYMAP [7] (ANCESTRYMAP see Web Resources) to check the plausibility of parental frequencies provided in the prior files. Prior frequencies with likelihood ratio statistic (S) >10 were replaced with frequencies of Utah residents from the CEPH collection representative of north and west European ancestry (CEPH) and of Nigerian Yorubans (YRI) from the HapMap [63] Project (HAPMAP see Web Resources). SNPs with S >10 score that were not represented in HapMap were excluded. All AIMs had at least a 30% allele difference between African and European populations. Our final set of AIMs included 1440 SNPs for case-only analysis, and 800 SNPs for case-control analysis with the average σ (absolute allele frequency differences between 2 ancestry populations) of 0.56 for both AIM panels.

#### Admixture mapping

We used ANCESTRYMAP [7] as the main tool for AM primarily because it gets unique LGS local scores, which points to a possible association not only at a given maker but also at any chromosomal position. ANCESTRYMAP [7] uses a Hidden Markov Model (HMM) for estimating ancestry along the genome. A Markov Chain Monte Carlo (MCMC) is used to account for uncertainty in HMM by comparing the likelihood of each locus as being associated with the disease versus being a locus unrelated to disease (the log base 10 of this likelihood ratio is LOD score). To obtain a genome-wide assessment of a disease locus, a genome-wide score was provided by averaging the locus specific LOD at each point. We considered a locus specific LOD score >5 as significant at the genome-wide level, which is quite conservative considering that a genome-wide score >2 can be declared significant [3]. ANCESTRYMAP requires a disease risk distribution as a prior to be used in a Bayesian likelihood ratio test. We initially investigated the genome-wide score under a series of disease risk priors from 0.4 to 3 times increased risk of disease due to one copy of European ancestry under the multiplicative genetic model. Prior distribution of risks was given by a gamma distribution with a mean increase in risk of 1.6-fold, with a standard deviation of 0.3. A LOD score greater than 2 was considered as suggestive and greater than 5 was considered to have genome-wide significance [3], [7]. We implemented this prior distribution by testing a grid of 27 risk models from 0.4 to 3.0, weighting the LOD scores obtained in each model according to the distribution. We ran ANCESTRYMAP for a burn-in period of 100 iterations with 200 follow-on iterations. In order to narrow down the estimate of disease risk we used a weighted risk range between 1.3 and 1.6 at 0.01 steps. We repeated the analysis using only females and then only males. We also repeated the analyses using alternate AIMs (odd and even) to verify that our results were not influenced by any individual AIM. To confirm that the chosen risk model distribution had no effect on our results we ran an unweighted risk distribution (from 0.4 to 3), as well as a fixed risk model at 1.5.

In order to confirm the existence of the detected peaks, we used 1726 controls with similar parameters as our case-only analysis. Our case-control analysis used ANCESTRYMAP, which calculates a Z distributed case-control statistic (Table S2).

We also used the program ADMIXMAP [6] (ADMIXMAP see Web Resources) to validate our previous results. ADMIXMAP uses a similar MCMC algorithm as ANCESTRYMAP to model probability distributions conditional on genotype, phenotypic values and *a priori* ancestral genotype frequencies. Models from ADMIXMAP calculate results in terms of standard normal Z-statistics and p-values. We applied 20,000 MCMC iterations with 1000 burn-in iterations in ADMIXMAP. Individual admixture estimates from ANCESTRYMAP were validated using both ADMIXMAP and STRUCTURE (10,000 MCMC iterations and 50,000 burn-in iterations). ADMIXMAP Z-statistics (and P-values) were compared to ANCESTRYMAP LOD scores at each genotyped marker. Tests for Case-only and Case-control design were carried with both programs. Bearing in mind that the more powerful Case-only design also had a higher density of variants [1440 AIMs for ANCESTRYMAP and 1355 AIMs (no AIMs from X chromosome) for ADMIXMAP, versus 800 AIMs/743 AIMs for the Case-control design], the peaks identified were at similar locations of the genome (Table S2).

#### Computer simulations to evaluate empirical statistical significance

To evaluate the empirical significance of the case-only genome-wide LOD score, we simulated 100 replicate data sets (1032 cases) without the disease locus using ANCESTRYMAP. We generated a normal distribution of the best scores attained and calculated the probability that our best marker score (6.28) would have occurred by chance.

### Stage 2: Follow up of 2q22–24 admixture signal using Case-Control analysis

After QC our analysis included 1525 AA SLE cases and 1810 AA healthy controls. We genotyped 347 highly informative AIMs to detect hidden population structure and correct for spurious associations. To assess robustness of these results we analyzed 3968 EA cases and 3542 EA.

In order to follow the admixture signal within 2q22–24, we genotyped 284 SNPs from 20 candidate genes. The peak and 95% CI of the 2q22–24 region spanned about 21 MB. We used logistic regression in PLINK, with individual admixture estimates as a covariate to identify and remove individual outliers, and to correct for admixture and population stratification in our data analysis. We increased our sample sets using out-of-study controls for a total of 1525 cases and 4485 controls in AA, and 3968 cases and 9750 controls in EA.

#### Population structure estimation

We estimated global admixture proportions of our AA samples using STRUCTURE. AIMs were selected for each dataset based on the platform in which they were genotyped. We used 216 AIMs to estimate admixture proportions in CC AA samples, 755 AIMs for DHS samples, and 642 AIMs for SAGE and HABC samples. CEPH and YRI HapMap populations served as fixed prior populations. The optimal number of admixture components was determined using methods described by Evanno [64]. Global ancestry of EA was estimated using 748 common SNPs overlapping all populations using EIGENSTRAT.

#### Local ancestry estimation

We estimated local ancestry proportions for AA and EA using STRUCTURE and corrected for European ancestry as a covariate in the logistic regression procedure in PLINK. We only selected markers within the admixture peak (2q22–24) for correct estimation of our area of interest. Estimated average European ancestry was similar for AA cases and controls (17.5±9.0% vs. 17.3±9.1%, P_t-test_ = 0.48). For AA, we used STRUCTURE with the linkage model, CEPH and YRI HapMap populations as fixed prior populations with seven SNPs to estimate the local European ancestral proportions for DHS, and nine SNPs for SAGE, HABC and WTCCC. We estimated local ancestry at the three SNPs of interest for Stage1-AA samples using ANCESTRYMAP with 195 genome-wide AIMs and averaged their European ancestry proportion. EA samples with >95% European ancestry were included in analysis.

We confirmed local ancestry estimates for AA samples using a PCA based local ancestry deconvolution method [65] implemented in PCAdmix. Phased CEPH and YRI HapMap populations were used as references. Because the aforementioned method requires phased known haplotypes, we used ten iterations in BEAGLE [66] to phase AA genotypes (149 SNPs). Local ancestry estimation was restricted to the coordinates between 138 MB and 164 MB, and created eight moving windows of 20 SNPs. We used the estimated ancestral state assignment (European or African) to group individuals with two European haplotypes, mixed haplotypes, or two African haplotypes, and extracted their genotypes for rs1990760, rs10930046 and rs13023380. We calculated F_ST_ [67] between CEPH (N = 87), LWK (N = 110), MKK (N = 156), Ghanaian individuals (N = 92), YRI (N = 147), AA with two European haplotypes (N = 129), and AA with two African haplotypes (N = 2124) for each variant using Arlequin [68]. Allele frequencies were compared to assign the most likely origin of the risk allele. Additionally we compared the LD structure of these groups for our three SNPs of interest.

#### Population stratification

In order to account for the effect of global population stratification on allelic association in AA, we used European admixture proportion as a logistic regression covariate. We used the first four principal components in a logistic regression model to correct for EA population stratification. Also, to account for the effect of local admixture on allelic association, we used local ancestry proportion as a logistic regression covariate for both AA and EA.

#### Population attributable risk

Population Attributable Risk (PAR) was estimated for the set of (rs1990760, rs10930046 and rs13023380) for AA and EA; and for the set (rs1990760 and rs13023380) for AA and EA. We followed the formula:
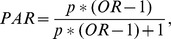
Where p is the frequency of the risk allele for controls, and OR is the odds ratio for that allele. The joint PAR was calculated as:
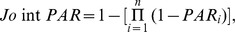
Where n denotes the number of SNPs included in the calculation [21], [69].

#### Locus-specific ancestry risk

We estimated the locus-specific ancestry risk ratio (λ), defined as the increased disease risk due to one copy of European ancestry allele at a specific bi-allelic locus by:
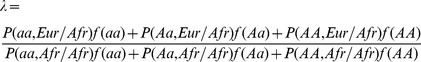
(1)where P(aa, 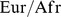), P(Aa, 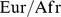) and P(AA, 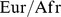) are genotype frequencies for aa, Aa and AA genotypes at the locus with one copy of the European ancestry allele, respectively; P(aa, Afr/Afr), P(Aa, Afr/Afr) and P(AA, Afr/Afr) are genotype frequencies for aa, Aa, AA genotypes at the locus with zero copy of the European ancestry allele, respectively. The f(aa), f(Aa), f(AA) denote the disease penetrance for each genotype that we assume constant between ancestry populations. For our particular case, “a” is used for the protective “G” allele of the three independent SNPs identified in this study, and “A” stands for the risk allele “A”. Let the “a” allele frequency is p and q in European and African ancestry population, respectively, and assuming HWE holds for the estimated locus, formula (1) can be further expressed as:
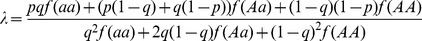
(2)Dividing the numerator and denominator in equation (1) by f(AA), we have:
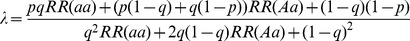
(3)where RR(aa) and RR(Aa) are the disease relative risk ratio for genotype aa and Aa compared with AA, respectively. For less common diseases, like SLE, the relative risk ratio can be further approximated by odds ratio (OR); therefore, we estimated λ as
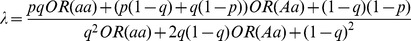
(4)where OR(aa) and OR(Aa) are the disease odds ratios for the aa and Aa genotype relative to AA genotype. The OR was estimated from our case-control analysis in AAs assuming a multiplicative genetic model and the allele frequencies of the three investigated SNPs in European and African ancestry populations are approximated by their frequencies in HapMap CEU and YRI samples.

#### Stratified ancestry risk

To assess whether European ancestry at 2q22–24 is associated with increased disease risk, we used a subset (N = 716) of our CC_AA_ cases) and repeated our admixture scan. These samples were also included in our initial AM that contained the genotype for the most diverse SNP rs10930046 (Fst_CEU-YRI_ = 0.38; In_CEU-YRI_ = 0.23), then we ran an admixture scan using ANCESTRYMAP separately for the three different genotypes (N_AA_ = 279, N_AG_ = 323, N_GG_ = 114) using both a fixed risk model of 1.5, and the previously described weighted risk model distribution. We compared the LOD score across genotypes at 2q22–24.

### Stage 3: Imputation-based fine-map analysis

#### Imputation-based association analysis

In order to optimize the efficiency of our experiment, we performed an imputation-based analysis on *IFIH1*. There are two components to this approach: (i) predicting (“imputing”) cohort genotypes, and (ii) analyzing association between cohort genotypes and phenotypes. In order to impute genotypes for study samples, existing models for population genetic variation across multiple markers were leveraged. Imputation was also used to estimate missing genotypes. Imputation was performed using MACH [22], [70], which provides a quantitative assessment of estimate uncertainty (r^2^) [70]. Allelic association results were consistent when accounting for imputation uncertainty using mach2dat [71].

A subset of our study samples, both of AA and EA, were genotyped on the ImmunoChip array, which provides a finer map with 233 *IFIH1* SNPs. To investigate whether there were new causative variants in the *IFIH1* region (162,817,515 to 162,901,398 bp, build 36), we used fine mapped controls as a reference panel to impute our study samples.

For AA imputation, the target sample set contained 1525 cases and 1810 controls for AA genotyped for 22 SNPs, 949 DHS samples with 11 SNPs, 942 SAGE samples with 27 SNPs, and 784 HABC samples with 27 SNPs from *IFIH1* (162,817,515 to 162,901,398 bp). Our reference panel had 207 AA controls with 233 SNPs in and around *IFIH1*. After QC, 123 SNPs were used for imputation. Imputation results contained 74 SNPs (61 SNPs of which were not genotyped in the target set) with r^2^≥90% and MAF>1%. Of these 74 SNPs there were 64 SNPs with P-values<0.05 and 41 SNPs with P values<5×10^−5^ (Table S6). The rationale for using a high threshold for imputation quality (r^2^≥90%) was to reduce bias due to different genotyping platforms and different number of variants genotyped on those platforms.

For EA imputation, the target sample contained 3968 cases and 3542 controls with 22 SNPs, 2267 SAGE samples with 27 SNPs, 1449 HABC samples with 27 SNPs, and 2492 samples with six SNPs in and around *IFIH1*. Our reference sample had 594 controls with 233 SNPs and 126 SNPs with MAF≥1% were used for imputation (Most of them have low allele frequencies and only 13 SNPs have MAF>5%). As a result of imputation, only 36 SNPs passed QC(HWE>0.001, MAF≥1% and r^2^≥90%). Besides 4 genotyped SNPs with P<0.05, no additional imputed SNP with P<0.05 was found. (Table S7).

#### Conditional analysis

In order to identify if there were other independent SNPs associated to SLE, we performed a conditional SNP analysis using WHAP [72]. For AA and EA, first we performed two-SNP pair-wise conditional analysis to identify independent effect of each SNP, starting with the most significant SNP. If the most significant SNP does not explain the entire association of the pair, then the other SNP is considered as independent SNP. Once we identified all possible independent SNPs, we then used the same conditional procedure using the two most significant independent SNPs and so on until all the associations were explained (Figure 2B, 2C for AA and Figure 2E, 2F for EA). We also performed conditional logistic regressions in SAS to reassess the independence of each SNP identified earlier. We stratified each one of the three variants (rs13023380, rs10930046 and rs1990760) while including the other two SNPs in the model as covariates. Independence of each SNP was determined if the SNPs in the model remained significant [73]. To see if any haplotype (based on the independent SNPs) could explain the whole association, we also used conditional haplotype analysis. The haplotypes were constructed using the EM algorithm implemented in WHAP.

### Stage 4: Experimental validation

#### Cloning, site-directed mutagenesis, and transient transfection

The full length *IFIH1* cDNA was fused with a C-terminal GFP and cloned under a *CMV* promoter with neomycin resistance gene in an expression-ready mammalian expression vector (M46- P receiver; Capital Bioscience, MD see Web Resources). cDNA sequence was confirmed by sequencing in both directions.

Site-directed mutagenesis (‘G’>‘A’) was performed independently at both rs10930046 and rs1990760 in the parent clone using the site directed mutagenesis kit (Stratagene see Web Resources). After mutagenesis, all clones were sequenced in both directions to confirm the ‘G’>‘A’ conversion at these bases. No other base modifications were observed. Three clones (ancestral rs10930046–rs1990760 ‘G-G’, risk for rs10930046 and non-risk for rs1990760 ‘A-G’ and non-risk for rs1990760 and risk for rs10930046 ‘G-A’) were obtained and used in the allele-specific apoptosis and gene expression assays.

#### Fluorescent activated cell sorting (FACS) analysis for measurement of apoptosis

Vectors carrying *IFIH1* alternative alleles were transiently transfected in a myeloid leukemia K562 cell line. Cell survival was measured through an apoptotic assay (Annexin PE apoptotic kit, Becton Dickinson Bioscience see Web Resources) in flow cytometry (Fluorescent Activated Cell Sorting). Cells were stained with Annexin-PE and AAD and were analyzed as the percentage of cell death among GFP+ cells (transfected cell count) through Annexin+, and Annexin+ AAD+. Measurements were taken at 44, 50, 66, 74, 80, 86 and 92 hours. Only vectors without the *IFIH1* gene did not induce apoptosis in these cells.

Contingency tables were created for the counts of dead cells versus the count of healthy cells for cells carrying the risk allele ‘A’ versus the protective allele ‘G’. Counts were corrected using the Yates method when appropriate. We compared the proportion of dead cells between the ancestral ‘G-G’ for rs10930046–rs1990760, and the ‘A-G’ and ‘G-A’ cells using a Wilcoxon signed ranked test with continuity correction [74].

#### Expression analysis of apoptosis and inflammation related genes in *IFIH1* overexpressed cells

*IFIH1* cDNA (carrying protective alleles for rs10930046 and rs1990760, or the risk allele for rs10930046 or rs1990760) were transfected and GFP+ cells were sorted using FACs. Total RNA was isolated from these sorted cells, reverse transcribed and subjected to qPCR for: *GAPDH* (internal control), *IFIH1*, *NFκ-B1*, *NFκ-B2*, *RELA*, *TNF*, *MAPK8*, *CASP9*, *CASP8*, *IFNA*, *MAVS*, *IFIT1* and *MX1* genes. qPCR fold changes were calculated after normalizing with *GAPDH* expression. Fold changes in expression of these genes were again normalized to *IFIH1* expression. These experiments were performed in duplicates, independently repeated three times, and then differences were analyzed with a paired t-test with unequal variance. In some of the cases (*e.g.* IFN beta treatment), non-transfected controls were also used to prepare cDNA and qPCR for *IFNA* and *IFIH1* measurement.

#### Electrophoretic mobility shift assay (EMSA)

We amplified 150-mer DNA sequences surrounding SNP rs130233380 from homozygous ‘GG’ (protective genotype) and ‘AA’ (risk genotype) carrying patients using PCR (150-mer, forward primer- 5′-ATAACCATCCAGTTAAGAGATAGG and reverse primer-5′ ACTCAAGATGTGATTGCAAAA TAAGG). PCR products were sequenced to confirm ‘GG’ and ‘AA’ genotype and that these PCR products did not have any other base modification. EMSA was performed using non-radioactive EMSA kit (Invitrogen,USA see Web Resources) with SYBR which detects DNA at 1 ng sensitivity. Nuclear extracts were prepared from K562 and Jurkat cell lines with a nuclear extract isolation kit (EpiQuik Nuclear Extraction Kit, Epigentek, USA). Both PCR amplified DNA sequences were purified and incubated with the nuclear extracts for 25 minutes at 25°C with increasing the amount of nuclear extract. Reaction mixtures were loaded in 2.2% agarose gel (Figure 4B) and 2.5% agarose gel (Figure 4C), where a 150-mer DNA was separated out from the 150-mer free DNA. Non-specific DNA is a 140-mer PCR product from bisulfate treated genomic DNA that does not occur in the cell. DNA bands were visualized with the dye SYBR. Intensity (as a measure of DNA quantity) of the shifted band and free DNA bands were calculated with TOTALLABQUANT (Totallabquant Limited, UK see Web Resources) for each lane. The ratio of DNA quantity for the shifted band to the free DNA band in each lane was calculated as a measure of binding efficiency.

#### Identification of proteins from EMSA complex by 2D electrophoresis

EMSA reactions were performed in large scale as described above, and a native PAGE gel (precast, 4–15%, Biorad laboratories) was run in the first dimension. Gels were stained with SYBR Green to locate the DNA-bound protein band (Figure S4A). For the second dimension, the gel was rotated 90 degrees and continued to run for 6 hours and again stained with SYBR green to locate the position of DNA-bound protein band (Figure S4B). The same gel was stained with Coomassie protein stain (Figure S4C), and the protein band aligned with the DNA bound band (arrow). Bands were excised from the gel and sequenced in a mass spectrometry core facility (LMBCR see Web Resources). Gel bands were digested with Trypsin and used for MASS spectrometry sequencing with HPLC and MS/MS analysis by the Dionex UltiMate 3000 and ABI MDS Sciex Qstar Elite, respectively. Data obtained from these analyses were submitted to the MASCOT (Matrix Science) server for protein identification against the SwissProt protein database (2011). These bands constituted Nucleolin (*NCL*), Ku70 (*XRCC5*), and Ku80 (*XRCC6*) as major constituents of nuclear protein (Table S11).

#### Identification of proteins from EMSA complex by immobilized streptavidin agarose beads

EMSA reactions were performed in large scale (10×) with PCR products with biotin-labeled primers for rs13023380 of *IFIH1* and rs1143679 of *ITGAM* as control. EMSAs were confirmed as described earlier using aliquots of these reactions. Streptavidin coated agarose beads (Invitrogen, USA) were added and incubated with gentle rotary shaking for 1 hour to bind biotinylated DNA bound proteins with streptavidin beads. Unbound cellular proteins were removed with repeated washing (10×) with 1× EMSA buffer by centrifugation. SDS-PAGE protein dye was added to beads and heated to 95°C and spun for 5 minutes to take supernatant as eluent. Both beads and eluents were fractionated in an SDS-PAGE gel to separate the component proteins in separate bands. Gels were stained with Coomassie blue for proteins, and a distinct band was visible (Figure S4D; arrow) that was not present in *ITGAM* control. This band yielded *NCL* as a major constituent of the band (Table S11).

#### Release of EMSA bound DNA by anti-Nucleolin and anti-Ku70/80 antibodies

EMSA was performed with Jurkat cell nuclear extract using the same PCR products but with a reduced proportion to ensure all DNA was bound with protein and no free DNA was left. This protein-bound DNA was equally divided into several aliquots and antibodies against Actin, NCL, Ku70/80, or HSP90AA1/HSP90AB1 were added to each tube and incubated for 1 hour at room temperature. The 2.5% agarose gel was run, stained with SYBR Green, and photographed. The free DNA band again appeared in the lane where NCL or Ku70/80 antibodies were added.

#### EMSA with purified recombinant protein of Nucleolin and Ku70/Ku80

EMSA was performed using the same PCR products carrying ‘G’ or ‘A’ allele of rs13023380 with purified recombinant protein, NCL (from insect cells, ABCAM, USA) and Ku70/80 (from insect cells, Biorbyt, UK). Protein-bound DNA was shifted, and confirmed specific binding of those proteins with the specific DNA fragments.

#### Luciferase assay

The exact sequence for risk and protective allele that were used for EMSA were cloned before a minimal *TKmin* (Thymidylate Kinase) promoter-carrying vector expressing luciferase. HeLa cells were transiently transfected with only the vector, vector carrying only MCS (Multiple cloning sites), and risk and protective allele carrying 150-mer DNA sequences. Luciferase activity was measured (Xactagen Inc, USA) after 48 hrs of transfection using Gaussia Luciferase Assay (GLOW) with the Gaussia luciferase assay kit (catalog # 31001, Xactagen, LLC) per manufacturer's directions. Medium was aspirated from transfected cells, plated in white 96-well assay plates. Cells were washed once by addition and aspiration of 100 uL of GLum.1 assay buffer. 50 uL of GLum.1 assay reagent (assay buffer plus coelenterazine substrate) was then added to each well and incubated (dark) for 5 minutes prior to reading luminescence (SpectraMax L, Molecular Devices: 475 nm).

Each experiment had three replicates at each time interval, and each experiment was repeated eight times, thus totaling 24 data points for each construct. These measurements (Relative Luminiscence Units) were normalized to a ‘cell-only’ control. After normalization, the average values were used to draw the bar chart and statistical significance was calculated using a two-sided unequal variance t-test.

#### Conservation of the protective alleles across genomes

Available genome sequences for *IFIH1* were downloaded from the UCSC Genome Browser (USCS see Web Resources). In some cases sequences were missing from the alignment or obviously incomplete or incorrect (*e.g.* missing exons or having long regions of non-homologous sequence). In these cases genomic sequences were obtained from the NCBI sequencing Trace Archive (BLAST [75] see Web Resources) and aligned using ClustalW [76] (ClustalW see Web Resources).

#### Molecular modeling

Structure files were retrieved from the Protein Data Bank (Protein Data Bank see Web Resources):

3TMI: human RIG-I bound to dsRNA. X-ray diffraction to 2.9 Å.

4A2W: duck RIG-I without nucleic acid. X-ray diffraction to 3.7 Å.

3B6E: human IFIH1, DECH (helicase ATP-binding) domain, incorporating translated rs10930046 risk allele. X-ray diffraction to 1.6 Å.

2RQB: human IFIH1, helicase C-terminal domain, incorporating translated rs1990760 protective allele. NMR.

All protein structural figures were rendered in PyMOL (PyMOL see Web Resources).

Single-stranded RNA (pre-mRNA) secondary structure prediction was performed using CentroidFold [77].

### Web resources

The URLs for the data presented here are as follows:

PLINK, http://pngu.mgh.harvard.edu/~purcell/plink

STRUCTURE, http://pritch.bsd.uchicago.edu/structure.html

EIGENSOFT, http://genepath.med.harvard.edu/~reich/Software.htm

ANCESTRYMAP, http://genepath.med.harvard.edu/~reich/Software.htm

ADMIXMAP, http://homepages.ed.ac.uk/pmckeigu/admixmap

MACH, http://www.sph.umich.edu/csg/abecasis/MACH/index.html

HAPMAP, http://www.hapmap.org

dbGaP, http://www.ncbi.nlm.nih.gov/gap

Wellcome Trust Consortium, http://www.wtccc.org.uk

DHS, http://clinicaltrials.gov/ct2/show/NCT00344903

Ingenuity Pathway Analysis, http://www.ingenuity.com

Capital Bioscience, MD: http://www.capitalbiosciences.com/

Becton Dickinson Bioscience: http://www.bd.com/

Stratagene: www.stratagene.com/

Invitrogen,USA: www.invitrogen.com

Totallabquant: http://www.totallab.com

UCSC Genome Browser: http://genome.ucsc.edu

NCBI sequencing Trace Archive: http://blast.ncbi.nlm.nih.gov

ClustalW: http://www.clustal.org/clustal2/

Protein Data Bank: http://www.rcsb.org

PyMOL: http://www.pymol.org/

CentroidFold: http://www.ncrna.org

LMBCR: www.ouhsc.edu/lmbcr

miRBase: http://www.mirbase.org/

## Supporting Information

Figure S1 PCA-based population structure for AA and EA. We maximized the number of ancestral informative markers (AIMs) given these datasets were genotyped in different platforms: (A) 755 SNPs for CC_AA_ and DHS AA samples, (B) first 2 principal components using 163 SNPs for AA samples (SAGE and CC_AA_), (C) 401 SNPs EA and AA samples (SAGE, HABC and WTCCC).
(PDF)

Figure S2 Linkage disequilibrium blocks for AA African and AA European haplotypes. Linkage disequilibrium blocks for African-American (AA) individuals with 2 African haplotypes (AA_AFR_, N = 2124), AA with 2 European Haplotypes (AA_EUR_, N = 129), AA controls (N = 4485), HAPMAP CEPH (CEPH, N = 87), EA controls (N = 9750), HAPMAP Yoruba from Nigeria (YRI, N = 153), HAPMAP Luhya from Kenya (LWK, N = 110), and HAPMAP Masaai from Kenya (MKK, N = 156), and Ghana samples (N = 92). Values and color pattern shown on gray-scale were based on R-square between each pair of SNPs, values and colors shown in red were based on D′ between each pair of SNPs.
(PDF)

Figure S3 Gene network related to *IFIH1*. Solid lines show direct interactions, dashed lines show indirect relationships.
(PDF)

Figure S4 Identification of proteins from EMSA. (A) EMSA reaction was run in native PAGE gel and stained with SYBR to locate protein-bound DNA band (arrow). (B) Marker was excised from the gel and gel is rotated to 90 degree, ran again and stained with SYBR green to locate 2 d protein-bound DNA band (arrow). (C) The same gel was stained with Coomassie for visualizing protein and aligned DNA-bound protein band was excised and sequenced by MASS-spectometry. (D) EMSA bound biotinylated DNA-proteins complex were pulled down with streptavidin coated agarose beads and fractionated in an SDS-PAGE gel. Protein bands that were present only in *IFIH1* (arrows) in comparison to *ITGAM* (control) were sequenced. (E) The addition of the respective antibodies facilitated release of free DNA from the EMSA bound complex. Here EMSA was saturated such that there was no free DNA (EMSA lane). EMSA bound DNA was divided to 4 aliquots and antibodies were added in each tube, incubated for 1 hr and loaded in a native PAGE gel. Distinct free DNAs were released in the anti-NCL and anti-KU70/80 lanes, but not in the anti-actin lane. (F) EMSA was performed using nuclear protein extracts from Jurkat cells with 141-bp PCR products including either the protective ‘G’ or risk ‘A’ sequence at rs13023380. Both ‘G’ and ‘A’ allele-containing PCR products bound to a protein complex in the nuclear extracts. However, the ‘A’ allele bound with at least 2-fold reduced efficiency compared to the ‘G’ allele-carrying PCR product, as measured by the intensity of the shifted band relative to the free DNA band in the same lane. As a nonspecific (NS) DNA control, a 140-bp DNA sequence not present in the genome was created by PCR amplification of bisulfite-modified genomic DNA.
(PDF)

Figure S5 Geographical distribution of allele frequencies for our 3 independently associated SNPs in *IFIH1*. Allele frequency differences between populations of the Human Genome Diversity Project for our three independent SNPs of interest show a south-to-north gradient of increase in allele frequency of derived (risk) alleles. Source HGDP Selection browser (http://hgdp.uchicago.edu/cgi-bin/gbrowse/HGDP/) (accessed January 2012).
(PDF)

Figure S6 Alignment of genomic region surrounding rs13023380 for available primate genomes. (A) The base corresponding to rs13023380 is universally conserved as ‘G’. Sequence is shown reverse complement, in the direction of the pre-mRNA. (B) Nucleolin, in addition to binding dsDNA, is also a sequence-specific ssRNA-binding protein, particularly of intronic pre-mRNA species, controlling their processing and trafficking [40]. Loss of pre-mRNA structure may impair trafficking and splicing. The region of single-stranded pre-mRNA surrounding rs13023380 is predicted to have a great degree of secondary structure, with a strongly favorable folding free energy. Secondary structure prediction is shown from CentroidFold (ncrna.org); a ∼1 kb fragment surrounding the rs13023380 locus was used for alignment and RNA folding. Aligned sequences of human, chimpanzee, gorilla, and orangutan were used for folding. (C) The rs13023380 risk allele sequence disrupts formation of a strongly conserved stem-loop structure, and globally disrupts RNA folding. (D) Control RNA folding experiment with reverse complement sequence shows little secondary structure and essentially zero folding free energy, consistent with pre-mRNA reading direction in the “sense” orientation of (A)–(C). For all panels, heat color shows the probability of base pair formation, from 0 (blue) to 1 (red).
(PDF)

Table S1 Overview of study design, samples sizes, ethnicity and demographics. Our study comprised admixture mapping on African Americans (AA), followed by a case-control study on AA and individuals of European Ancestry (EA). Data sources: Oklahoma Medical Research Foundation (OMRF); Dallas Heart Study (DHS); University of Alabama at Birmingham (UAB); Study of Addiction Gene x Environment (SAGE); Health ABC (HABC); Wellcome Trust Consortium Case Control Study (WTCC). CC_AA_ and CC_EA_ are cases and controls from AA and EA samples genotyped at OMRF.
(DOCX)

Table S2 Admixture mapping signals generated by ANCESTRYMAP and ADMIXMAP using a low-density or high-density map. We report logarithm of odds (LOD) scores for ANCESTRYMAP in case-only design, and P-values for significance derived from the Z-distributed case-control statistic from ANCESTRYMAP and ADMIXMAP. The 95% confidence interval (CI) for each peak was calculated where the ANCESTRYMAP local ancestry significantly deviated from the mean global European. The strongest peak was found at 2q22.2–q24.3, spanning 21 megabases (MB).
(DOCX)

Table S3 Rationale for selecting 20 candidate genes at 2q22–24. In order to follow-up the peak at 2q22–24, we chose 20 genes to target on the basis of previous information about their function and reported clinical associations related with SLE. Positions based on genome build 36.
(DOCX)

Table S4 Follow-up case-control association results. Case-control association results for the follow-up study at selected candidate genes from the strongest admixture peak (2q22–q24) using 1525 cases and 1810 controls from CC_AA_ and 3968 cases and 3542 controls from CC_EA_ pinpoint IFIH1 as a candidate gene for SLE. * Allele frequencies given correspond to the A1 allele. Local ancestry correction is labeled as Pc, whereas P-value refers to the uncorrected value.
(DOCX)

Table S5 Genetic models of association of *IFIH1* with SLE. Genetic models at each independent SNP for African-Americans (AA) and European-Americans (EA) show that the best model for rs1990760 and rs130233890 are allelic, and dominant for rs10930046. The best model was chosen using the Akaike information criterion (AIC).
(DOCX)

Table S6 Imputation based association analysis for African Americans (N = 1525 cases; 4485 controls). Pc denotes the local ancestry-corrected P-value. ∧Rsq denotes the quality measure of the squared correlation between imputed and true genotypes. * denotes the 11 SNPs that were genotyped in DHS controls.
(DOCX)

Table S7 Imputation based association analysis for European Americans (N = 3968 cases; 9750 controls). ∧Rsq denotes the quality measure of the squared correlation between imputed and true genotypes. Pc stands for the P-value corrected for local ancestry.
(DOCX)

Table S8 Risk allele frequencies and Fst values between populations for three *IFIH1* SNPs. Local ancestry at three SNPs in *IFIH1* was estimated for AA. Individuals whose ancestral state was European (N = 129) and African (N = 2124) were selected, and their risk allele (“A”) frequency compared with allele frequencies in CEPH and YRI. F_ST_ was calculated between these groups.
(DOCX)

Table S9 Alignment of genomic region surrounding rs1990760 for available mammal genomes. The base corresponding to rs1990760 is nearly universally conserved as “G”, with a resulting alanine codon. Sequence is shown reverse complement, in the direction of the reading frame, showing five codons in either direction. Squirrel may have a threonine (Thr) side-chain; interestingly, tenrec may have a 2-amino acid deletion. Alignment of Chr2: 163124034–163124066, 33 bps (reverse complement).
(DOCX)

Table S10 Alignment of genomic region surrounding rs10930046 for available vertebrate genomes. The base corresponding to rs10930046 is universally conserved as “G”, with a resulting arginine codon. Sequence is shown reverse complement, in the direction of the reading frame, showing five codons in either direction. Alignment of Chr2: 163137967–163137999, 33 bps (reverse complement).
(DOCX)

Table S11 Mass Spectrometry sequencing and MASCOT database searches of 2D-gel and streptavidin agarose beads of EMSA.
(DOCX)

Text S1 Additional list of investigators.
(DOCX)
