# Supplementary figures and images for: Admixture Mapping in Lupus Identifies Multiple Functional Variants within IFIH1 Associated with Apoptosis, Inflammation, and Autoantibody Production
Source: PLoS Genet. 2013 Feb 18;9(2):e1003222. doi: 10.1371/journal.pgen.1003222 (PMC3575474; doi:10.1371/journal.pgen.1003222)

**Figure S1**

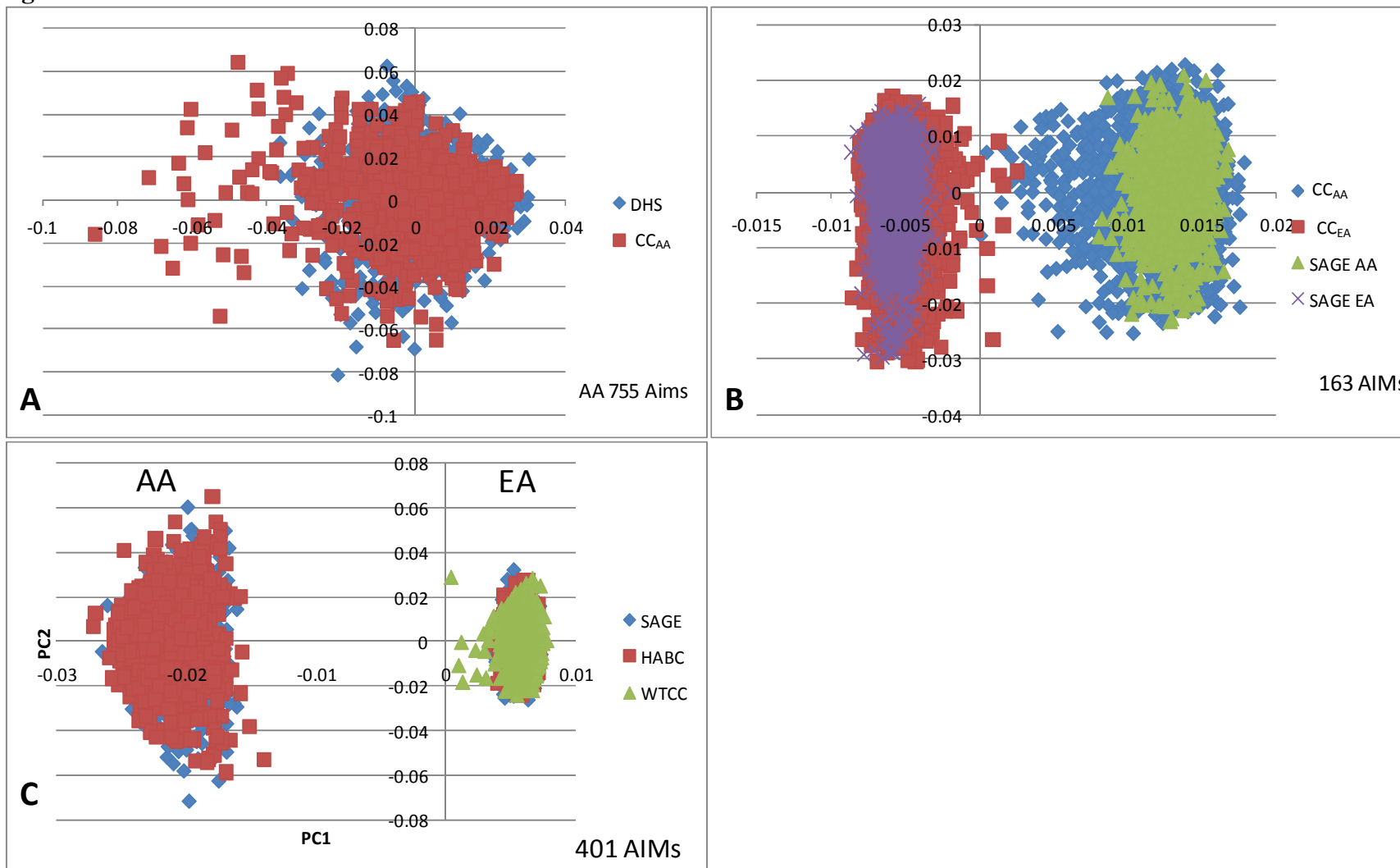

Supplement: Figure S1 — PCA-based population structure for AA and EA. We maximized the number of ancestral informative markers (AIMs) given these datasets were genotyped in different platforms: (A) 755 SNPs for CCAA and DHS AA samples, (B) first 2 principal components using 163 SNPs for AA samples (SAGE and CCAA), (C) 401 SNPs EA and AA samples (SAGE, HABC and WTCCC). (PDF) [file pgen.1003222.s001.pdf]

Figure S2.

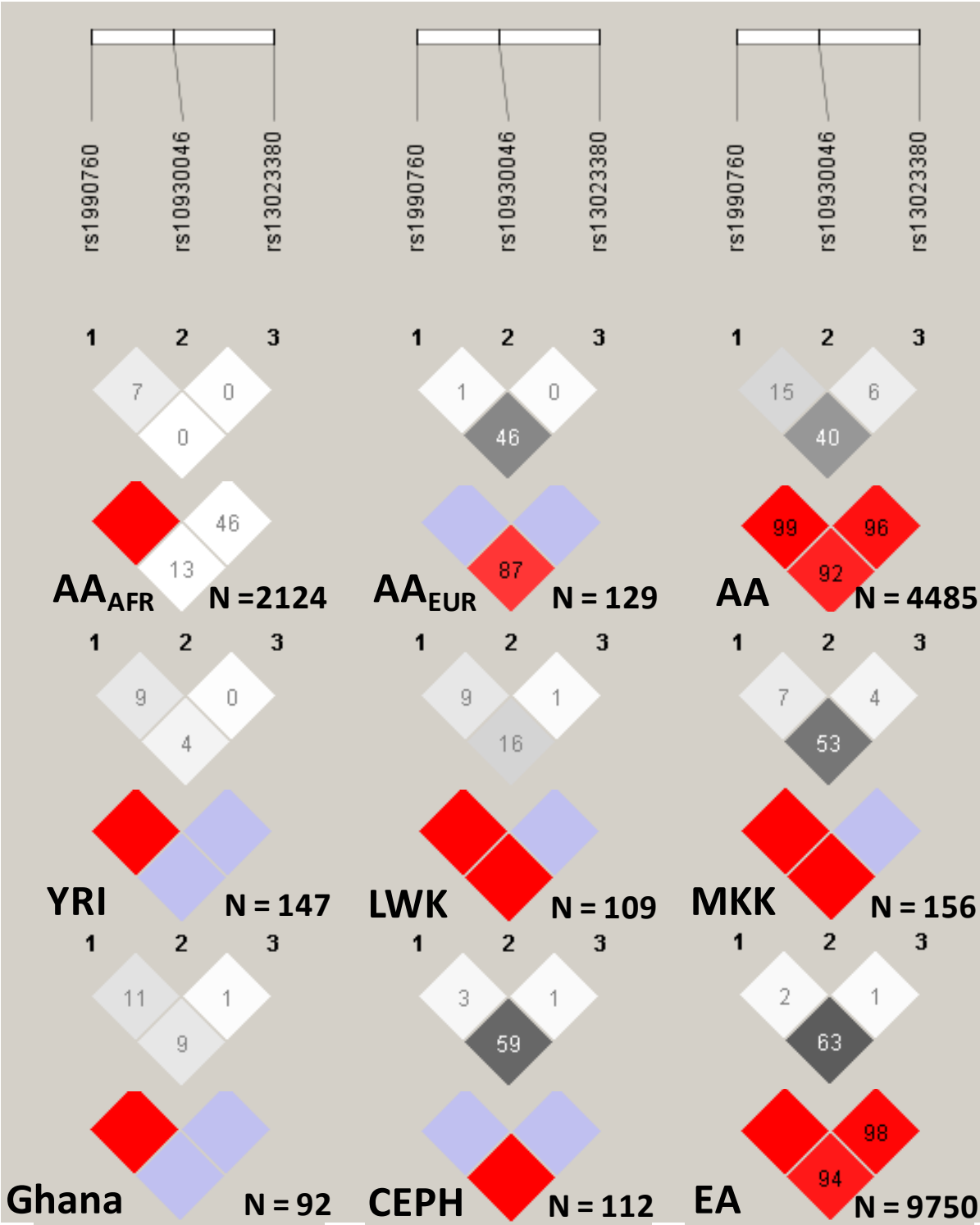

Supplement: Figure S2 — Linkage disequilibrium blocks for AA African and AA European haplotypes. Linkage disequilibrium blocks for African-American (AA) individuals with 2 African haplotypes (AAAFR, N = 2124), AA with 2 European Haplotypes (AAEUR, N = 129), AA controls (N = 4485), HAPMAP CEPH (CEPH, N = 87), EA controls (N = 9750), HAPMAP Yoruba from Nigeria (YRI, N = 153), HAPMAP Luhya from Kenya (LWK, N = 110), and HAPMAP Masaai from Kenya (MKK, N = 156), and Ghana samples (N = 92). Values and color pattern shown on gray-scale were based on R-square between each pair of SNPs, values and colors shown in red were based on D′ between each pair of SNPs. (PDF) [file pgen.1003222.s002.pdf]

**Figure S4**

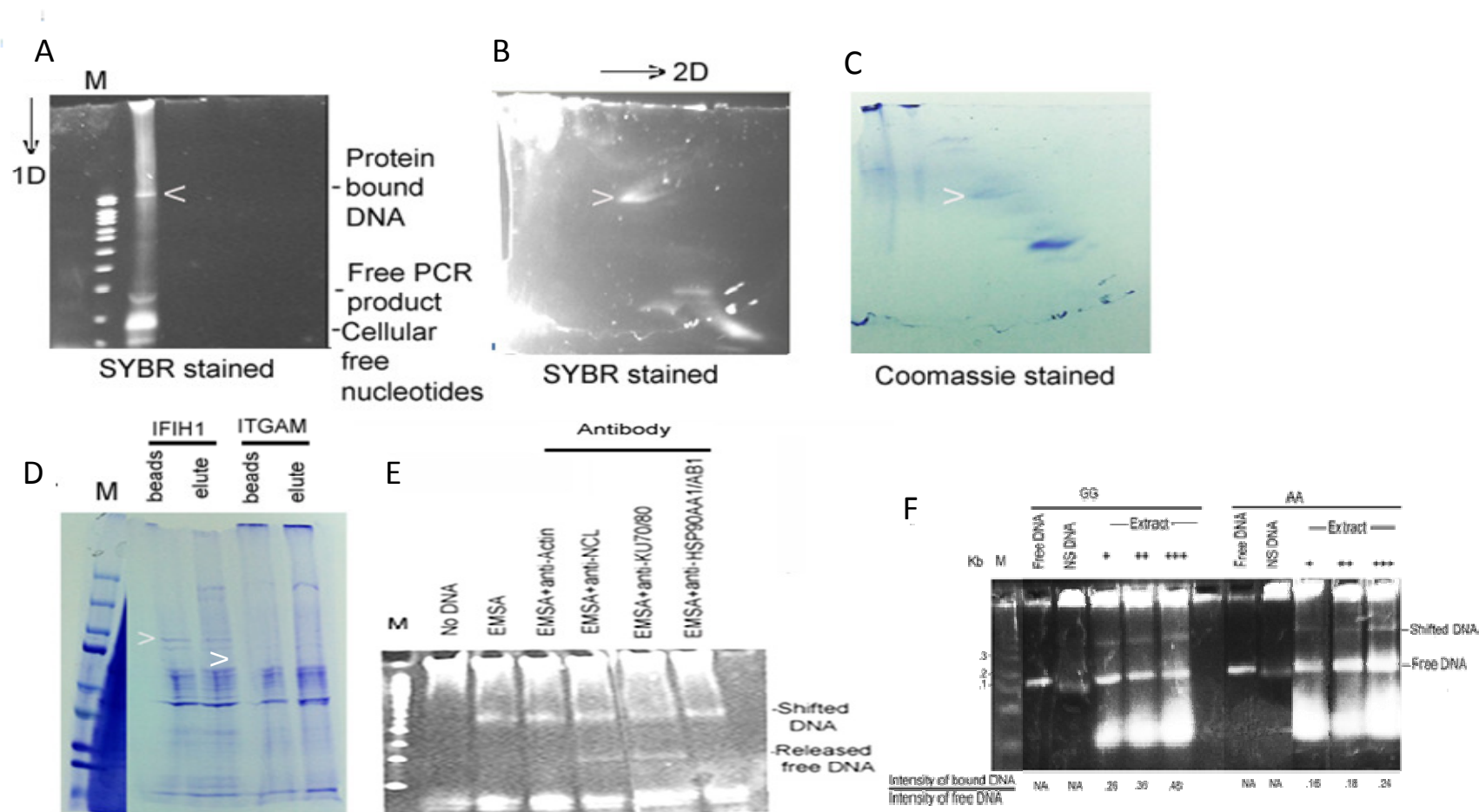

Supplement: Figure S4 — Identification of proteins from EMSA. (A) EMSA reaction was run in native PAGE gel and stained with SYBR to locate protein-bound DNA band (arrow). (B) Marker was excised from the gel and gel is rotated to 90 degree, ran again and stained with SYBR green to locate 2 d protein-bound DNA band (arrow). (C) The same gel was stained with Coomassie for visualizing protein and aligned DNA-bound protein band was excised and sequenced by MASS-spectometry. (D) EMSA bound biotinylated DNA-proteins complex were pulled down with streptavidin coated agarose beads and fractionated in an SDS-PAGE gel. Protein bands that were present only in IFIH1 (arrows) in comparison to ITGAM (control) were sequenced. (E) The addition of the respective antibodies facilitated release of free DNA from the EMSA bound complex. Here EMSA was saturated such that there was no free DNA (EMSA lane). EMSA bound DNA was divided to 4 aliquots and antibodies were added in each tube, incubated for 1 hr and loaded in a native PAGE gel. Distinct free DNAs were released in the anti-NCL and anti-KU70/80 lanes, but not in the anti-actin lane. (F) EMSA was performed using nuclear protein extracts from Jurkat cells with 141-bp PCR products including either the protective ‘G’ or risk ‘A’ sequence at rs13023380. Both ‘G’ and ‘A’ allele-containing PCR products bound to a protein complex in the nuclear extracts. However, the ‘A’ allele bound with at least 2-fold reduced efficiency compared to the ‘G’ allele-carrying PCR product, as measured by the intensity of the shifted band relative to the free DNA band in the same lane. As a nonspecific (NS) DNA control, a 140-bp DNA sequence not present in the genome was created by PCR amplification of bisulfite-modified genomic DNA. (PDF) [file pgen.1003222.s004.pdf]

**Figure S5**

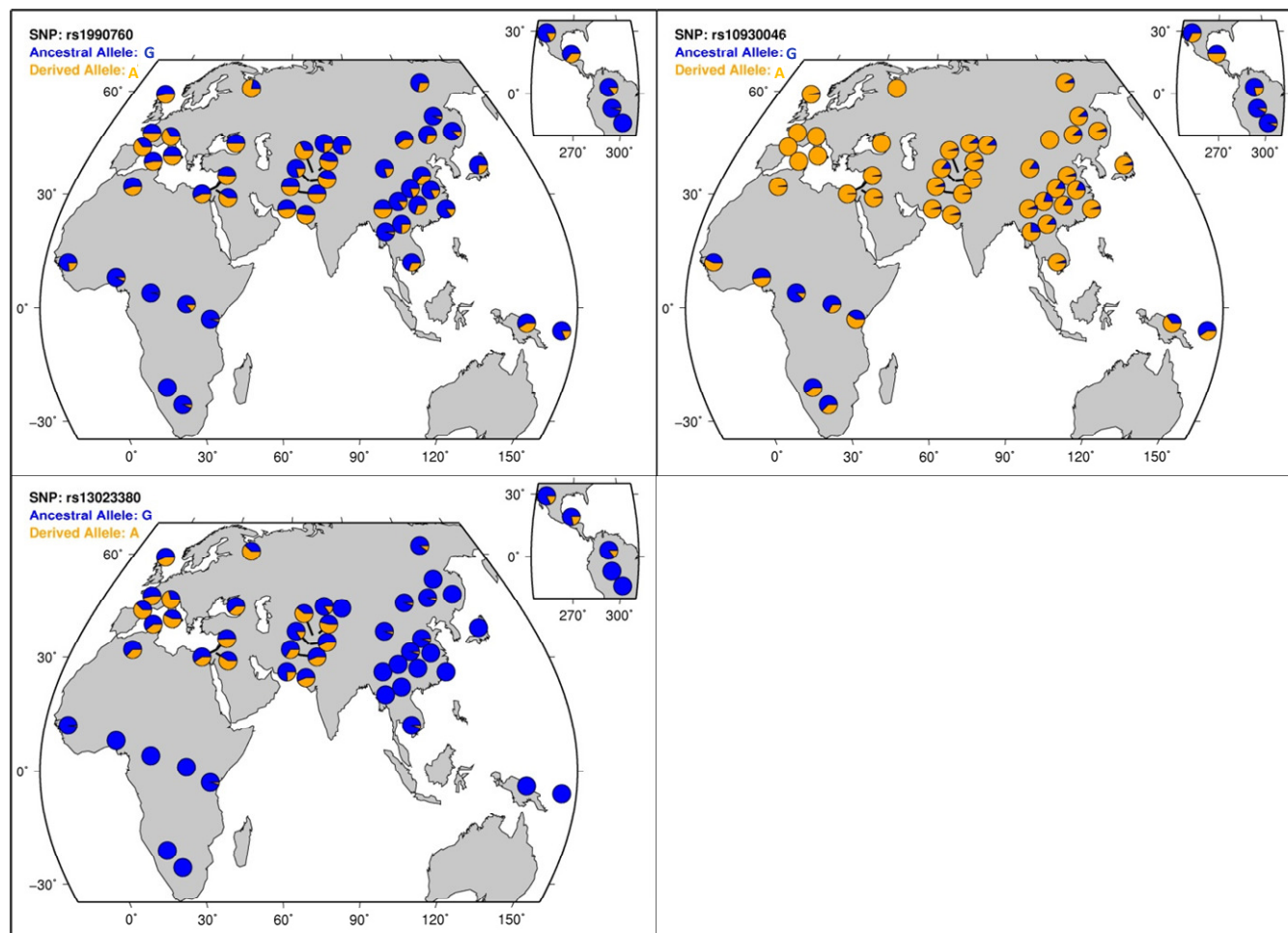

Supplement: Figure S5 — Geographical distribution of allele frequencies for our 3 independently associated SNPs in IFIH1. Allele frequency differences between populations of the Human Genome Diversity Project for our three independent SNPs of interest show a south-to-north gradient of increase in allele frequency of derived (risk) alleles. Source HGDP Selection browser (http://hgdp.uchicago.edu/cgi-bin/gbrowse/HGDP/) (accessed January 2012). (PDF) [file pgen.1003222.s005.pdf]
