# Supplementary material for: Admixture Mapping in Lupus Identifies Multiple Functional Variants within IFIH1 Associated with Apoptosis, Inflammation, and Autoantibody Production
Source: PLoS Genet. 2013 Feb 18;9(2):e1003222. doi: 10.1371/journal.pgen.1003222 (PMC3575474; doi:10.1371/journal.pgen.1003222)
Supplement: Figure S3 — Gene network related to IFIH1. Solid lines show direct interactions, dashed lines show indirect relationships. (PDF) [file pgen.1003222.s003.pdf]

Figure S3

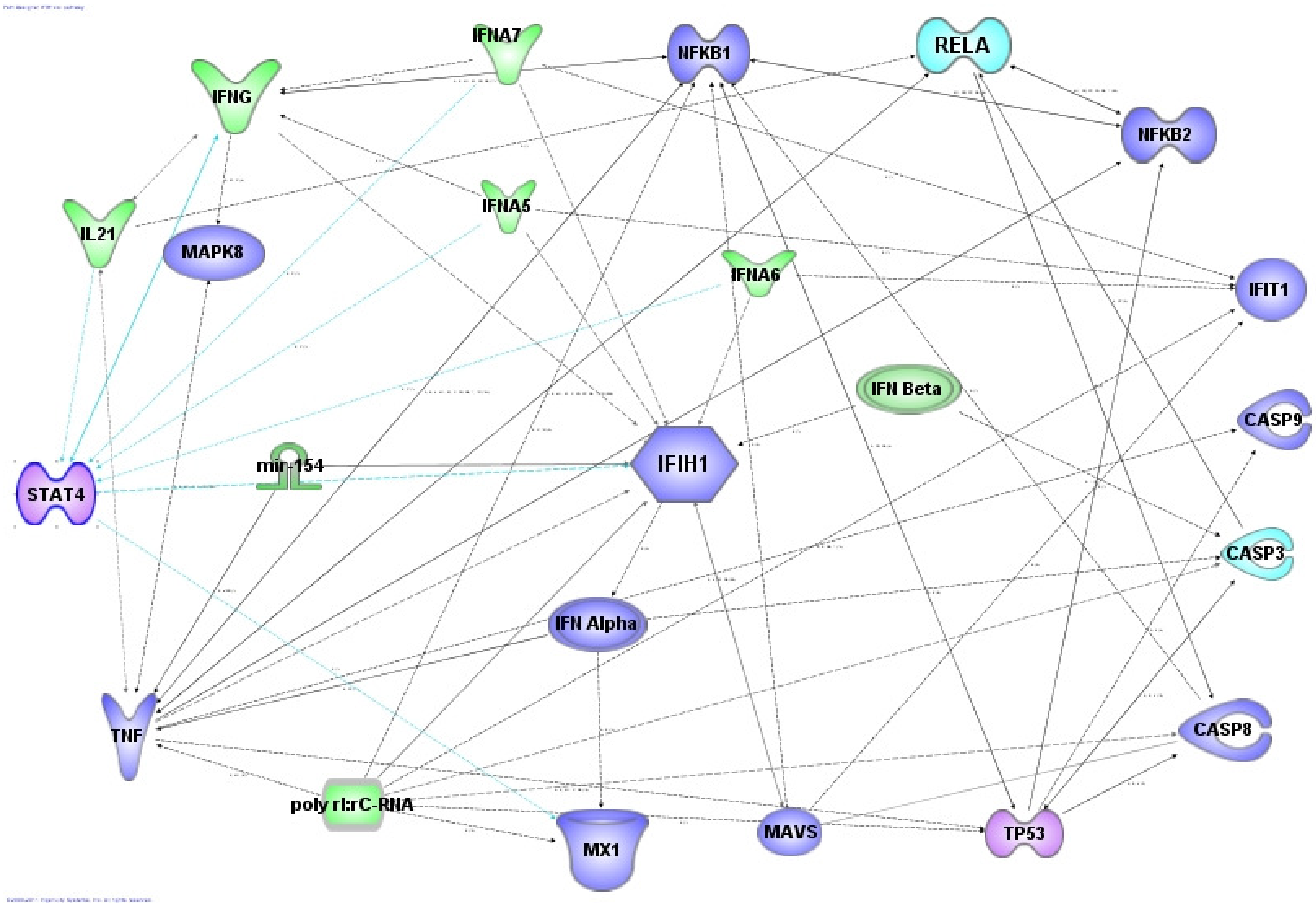
IFNA7
NFKB1
RELA
IFNG
NFKB2
IFNA5
IL21
MAPK8
IFNA6
IFIT1
IFN Beta
CASP9
mir-154
IFIH1
STAT4
CASP3
IFN Alpha
CASP8
TNF
poly rI:rC-RNA
MAVS
TP53
MX1
