# Supplementary material for: Admixture Mapping in Lupus Identifies Multiple Functional Variants within IFIH1 Associated with Apoptosis, Inflammation, and Autoantibody Production
Source: PLoS Genet. 2013 Feb 18;9(2):e1003222. doi: 10.1371/journal.pgen.1003222 (PMC3575474; doi:10.1371/journal.pgen.1003222)

Figure S6

A

Baboon
CCATGGCTTTTGGGCTGGTCTTAGGCA**C**TTTCTGTCATTGTAGGCAAAGCTGTTCTTCTTTGTGTTGTTTACAAAA
Rhesus
CCATGGCTTTTGGGCTGGTCTTAGGCA**C**TTTCTGTCATTGTAGGCAAAGCTGTTCTTCTTTGTGTTGTTTACAAAA
Human
CCATGGCTTTTGGGCTGGTCTTAGGCA**C**TTGCTGTCATTGTAGGCAAGGCTGTTCTTTTTTGTGTTGTTTACAAAA
Chimp
CCATGGCTTTTGGGCTGGTCTTAGGCA**C**TTGCTGTCATTGTAGGCAAGGCTGTTCTTTTTTGTGTTGTTTACAAAA
Gorilla
CCATGGCTTTTGGGCTGGTCTTAGGCA**C**TTGCTGTCATTGTAGGCAAGGCTGTTCTTTTTTGTGTTGTTTACAAAA
Orangutan
CCATGGCTTTTGGGCTGGTCTTAGGCA**C**TTGCTGTCATTGTAGGCAAGGCTGTTCTTTTTTGTGTTGTTTACAAAA
Gibbon
CCATGGCTTTTGGGCTGGTCTTAGGCA**C**TTTCTGTCATTGTAGGCAAGACTGTTCTTTTTTGTGTTGTTTACAAAA
Marmoset CCATGGCTTTTGGACTGGTCTTAGACA**C**TTTCT----TTGTAGGCAAAGCTGTTCTTTTTTCTGTTGTTTACAAAA
Consensus
CCAUGGCUUUUGGGCUGGUCUUAGGCA**C**UUKCUgucaUUGUAGGCAARGCUGUUCUUYUUUGUGUUGUUUACAAAA

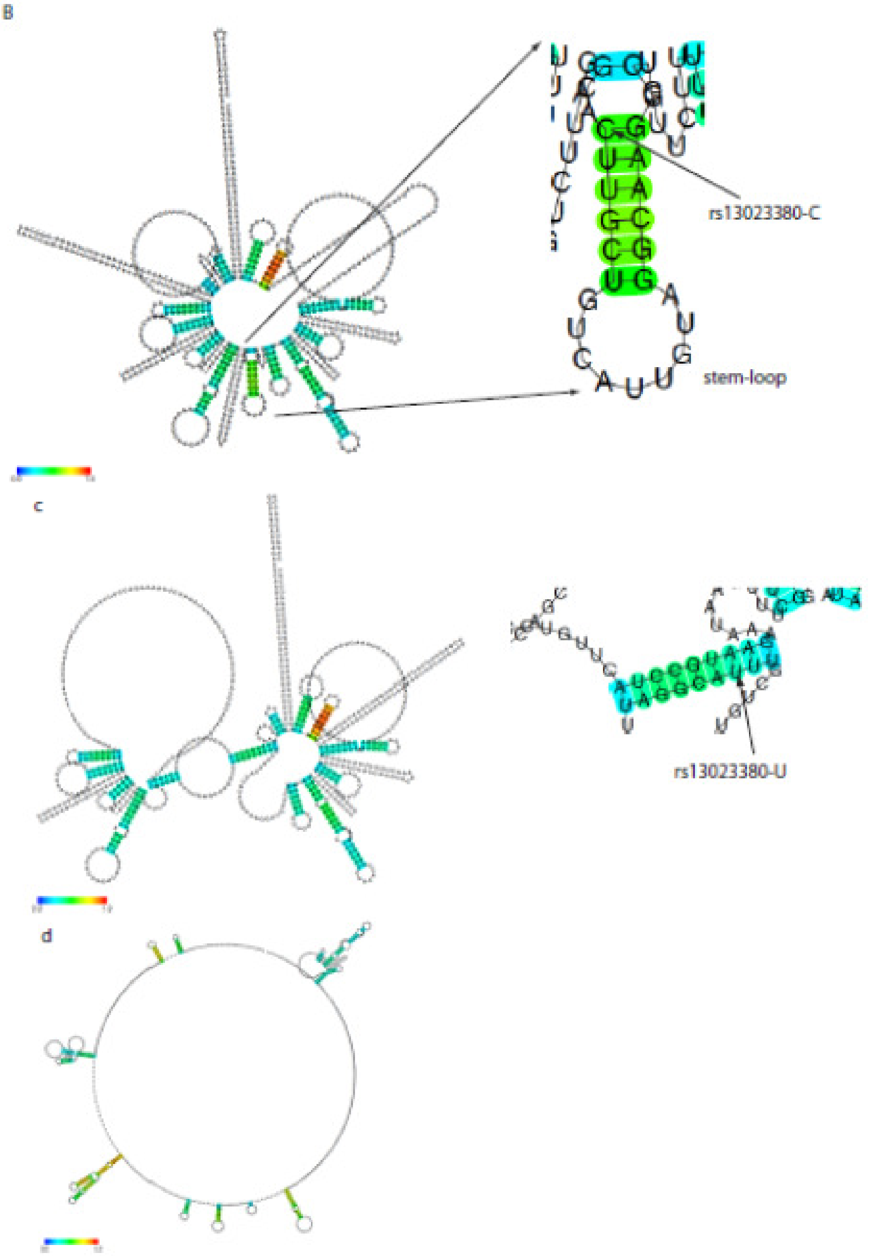

Supplement: Figure S6 — Alignment of genomic region surrounding rs13023380 for available primate genomes. (A) The base corresponding to rs13023380 is universally conserved as ‘G’. Sequence is shown reverse complement, in the direction of the pre-mRNA. (B) Nucleolin, in addition to binding dsDNA, is also a sequence-specific ssRNA-binding protein, particularly of intronic pre-mRNA species, controlling their processing and trafficking [40]. Loss of pre-mRNA structure may impair trafficking and splicing. The region of single-stranded pre-mRNA surrounding rs13023380 is predicted to have a great degree of secondary structure, with a strongly favorable folding free energy. Secondary structure prediction is shown from CentroidFold (ncrna.org); a ∼1 kb fragment surrounding the rs13023380 locus was used for alignment and RNA folding. Aligned sequences of human, chimpanzee, gorilla, and orangutan were used for folding. (C) The rs13023380 risk allele sequence disrupts formation of a strongly conserved stem-loop structure, and globally disrupts RNA folding. (D) Control RNA folding experiment with reverse complement sequence shows little secondary structure and essentially zero folding free energy, consistent with pre-mRNA reading direction in the “sense” orientation of (A)–(C). For all panels, heat color shows the probability of base pair formation, from 0 (blue) to 1 (red). (PDF) [file pgen.1003222.s006.pdf]
