# Supplementary material for: Admixture Mapping in Lupus Identifies Multiple Functional Variants within IFIH1 Associated with Apoptosis, Inflammation, and Autoantibody Production
Source: PLoS Genet. 2013 Feb 18;9(2):e1003222. doi: 10.1371/journal.pgen.1003222 (PMC3575474; doi:10.1371/journal.pgen.1003222)
Supplement: Table S1 — Overview of study design, samples sizes, ethnicity and demographics. Our study comprised admixture mapping on African Americans (AA), followed by a case-control study on AA and individuals of European Ancestry (EA). Data sources: Oklahoma Medical Research Foundation (OMRF); Dallas Heart Study (DHS); University of Alabama at Birmingham (UAB); Study of Addiction Gene x Environment (SAGE); Health ABC (HABC); Wellcome Trust Consortium Case Control Study (WTCC). CCAA and CCEA are cases and controls from AA and EA samples genotyped at OMRF. (DOCX) [file pgen.1003222.s007.docx]

**Table S1.** **Overview of study design, samples sizes, ethnicity and demographics.** Our study comprised admixture mapping on African Americans (AA), followed by a case-control study on AA and individuals of European Ancestry (EA). Data sources: Oklahoma Medical Research Foundation (OMRF); Dallas Heart Study (DHS); University of Alabama at Birmingham (UAB); Study of Addiction Gene x Environment (SAGE); Health ABC (HABC); Wellcome Trust Consortium Case Control Study (WTCC). CC_AA_ and CC_EA_ are cases and controls from AA and EA samples genotyped at OMRF.

| Study | Ethnicity | Data sets | Status | Male | Female | Total |
| --- | --- | --- | --- | --- | --- | --- |
| Admixture Mapping | AA | OMRF+UAB | Case | 84 | 948 | 1032 |
|  |  | DHS | Control | 729 | 997 | 1726 |
| Case-Control  analysis | AA | CC_AA_ | Case | 122 | 1403 | 1525 |
|  |  |  | Control | 574 | 1236 | 1810 |
|  |  | DHS | Control | 354 | 595 | 949 |
|  |  | SAGE | Control | 454 | 488 | 942 |
|  |  | HABC | Control | 340 | 444 | 784 |
|  | EA | CC_EA_ | Case | 364 | 3604 | 3968 |
|  |  |  | Control | 1180 | 2362 | 3542 |
|  |  | SAGE | Control | 996 | 1271 | 2267 |
|  |  | HABC | Control | 767 | 682 | 1449 |
|  |  | WTCCC | Control | 1238 | 1254 | 2492 |
