# Supplementary material for: Admixture Mapping in Lupus Identifies Multiple Functional Variants within IFIH1 Associated with Apoptosis, Inflammation, and Autoantibody Production
Source: PLoS Genet. 2013 Feb 18;9(2):e1003222. doi: 10.1371/journal.pgen.1003222 (PMC3575474; doi:10.1371/journal.pgen.1003222)
Supplement: Table S2 — Admixture mapping signals generated by ANCESTRYMAP and ADMIXMAP using a low-density or high-density map. We report logarithm of odds (LOD) scores for ANCESTRYMAP in case-only design, and P-values for significance derived from the Z-distributed case-control statistic from ANCESTRYMAP and ADMIXMAP. The 95% confidence interval (CI) for each peak was calculated where the ANCESTRYMAP local ancestry significantly deviated from the mean global European. The strongest peak was found at 2q22.2–q24.3, spanning 21 megabases (MB). (DOCX) [file pgen.1003222.s008.docx]

**Table S2.** **Admixture mapping signals generated by ANCESTRYMAP and ADMIXMAP using a low-density or high-density map.** We report logarithm of odds (LOD) scores for ANCESTRYMAP in case-only design, and P-values for significance derived from the Z-distributed case-control statistic from ANCESTRYMAP and ADMIXMAP. The 95% confidence interval (CI) for each peak was calculated where the ANCESTRYMAP local ancestry significantly deviated from the mean global European. The strongest peak was found at 2q22.2-q24.3, spanning 21 megabases (MB).

| Admixture signals | | Size  (Mb) | ANCESTRYMAP | | | ADMIXMAP | | |
| --- | --- | --- | --- | --- | --- | --- | --- | --- |
|  |  |  | High-density map | Low-density  map | | High-density map | Low-density  map | |
| Cytogenetic  Position | 95% CI |  | Case-only  (LOD) | Case-only  (LOD) | Case-control  (P-value) | Case-only  (P-value) | Case only  (P-value) | Case-control  (P-value) |
| 2q11.2-q12.1 | [84.26, 103.24] | 18.97 | 3.609 | 1.749 | 2.69x10^-1^ | 4.82x10^-3^ | 4.44x10^-2^ | 1.70x10^-1^ |
| **2q22.2-q24.3** | **[142.36, 163.6]** | **21.24** | **6.286** | **3.001** | **3.69x10^-2^** | **2.99x10^-8^** | **7.24x10^-5^** | **4.40x10^-3^** |
| 2q31.1-q32.3 | [169.46, 192.26] | 22.79 | 3.522 | 1.340 | 6.36x10^-2^ | 1.64x10^-4^ | 2.11x10^-2^ | 6.15x10^-1^ |
| 7q22.3-q31.1 | [105.78, 111.96] | 6.18 | 3.264 | 0.610 | 7.49x10^-2^ | 6.49x10^-6^ | 8.32x10^-2^ | 9.83x10^-1^ |
| 9q34.13-q34.3 | [133.6, 140.85] | 7.26 | 3.431 | -0.300 | 8.49x10^-1^ | 1.41x10^-5^ | 2.79x10^-2^ | 1.62x10^-1^ |
| 14q24.3 | [73.02, 76.64] | 3.61 | 2.439 | 0.277 | 9.34x10^-2^ | 3.84x10^-5^ | 7.00x10^-3^ | 1.20x10^-2^ |
| 19p12-q12 | [21.84, 35.97] | 14.13 | 3.204 | 0.820 | 8.63x10^-3^ | 1.15x10^-5^ | 1.13x10^-3^ | 5.97x10^-3^ |
