# Supplementary material for: Admixture Mapping in Lupus Identifies Multiple Functional Variants within IFIH1 Associated with Apoptosis, Inflammation, and Autoantibody Production
Source: PLoS Genet. 2013 Feb 18;9(2):e1003222. doi: 10.1371/journal.pgen.1003222 (PMC3575474; doi:10.1371/journal.pgen.1003222)
Supplement: Table S3 — Rationale for selecting 20 candidate genes at 2q22–24. In order to follow-up the peak at 2q22–24, we chose 20 genes to target on the basis of previous information about their function and reported clinical associations related with SLE. Positions based on genome build 36. (DOCX) [file pgen.1003222.s009.docx]

**Table S3. Rationale for selecting 20 candidate genes at 2q22-24.** In order to follow-up the peak at 2q22-24, we chose 20 genes to target on the basis of previous information about their function and reported clinical associations related with SLE. Positions based on genome build 36.

| **Gene** | **Start** | **End** | **Name** | **Reason for selection** |
| --- | --- | --- | --- | --- |
| *ARHGAP15* | 143,603,432 | 144,242,390 | Rho GTPase activating protein 15 | Expressed in lymphoid cells; recently reported that ArhGAP15 is a master negative regulator of neutrophil function |
| *ZEB2* | 144,862,052 | 144,994,385 | Zinc finger E-box binding homeobox 2 | Related to neurological activity (neurological manifestation is a diagnostic feature of lupus) |
| *ACVR2A* | 148,319,039 | 148,404,862 | Activin A receptor, type IIA | Recent report suggested that Acvr2a is a T-helper 17 (T_h_17) cell-specific gene induced during T_h_17 differentiation. Also, this gene is associated with cancer |
| *NMI* | 151,835,230 | 151,854,619 | N-myc (and STAT) interactor | The NMI protein interacts with all Signal Transducer and Activator of Transcription proteins ([STATs](http://en.wikipedia.org/wiki/STAT_protein); including STAT4, an SLE susceptibility gene) except [STAT2](http://en.wikipedia.org/wiki/STAT2) and augments STAT-mediated transcription in response to cytokines [IL-2](http://en.wikipedia.org/wiki/Interleukin_2) and [IFN-gamma](http://en.wikipedia.org/wiki/Interferon-gamma) |
| *TNFAIP6* | 151,922,350 | 151,944,805 | Tumor necrosis factor, alpha-induced protein 6 (*TNFAIP6*) | In the same family as *TNFAIP3* (an SLE susceptibility gene) |
| *RIF1* | 151,974,673 | 152,040,664 | RAP1 interacting factor homolog | DNA-binding protein, associated with cancer |
| *NEB* | 152,050,098 | 152,299,234 | Nebulin | Associated with cancer |
| *CACNB4* | 152,402,381 | 152,663,789 | Voltage-gated calcium channel, beta 4 subunit | Related to neurological activity, associated with idiopathic generalized epilepsy |
| *ARL5A* | 152,645,592 | 152,685,009 | ADP-ribosylation factor-like 5A | Between *STAM2* and *CACNB4,* strong LD |
| *STAM2* | 152,683,351 | 152,740,751 | Signal transducing adaptor molecule (SH3 domain and ITAM motif) 2 | Related to kinases and cytokine stimulation |
| *RPRM* | 154,042,097 | 154,043,567 | Reprimo, TP53 dependent G2 arrest mediator candidate | Associated with cancer |
| *GALNT13* | 154,436,671 | 155,018,734 | UDP-N-acetyl-alpha-D-galactosamine:polypeptide N-acetylgalactosaminyltransferase 13 (GalNAc-T13) | Associated with cancer |
| *KCNJ3* | 155,263,338 | 155,421,259 | Potassium channel, inwardly-rectifying, subfamily J, member 3 | Associated with cancer |
| *ACVR1C* | 158,097,149 | 158,193,644 | Activin A receptor, type IC | Binds to ACVR2A, a growth factor |
| *PKP4* | 159,021,721 | 159,246,186 | Plakophilin 4 | Adhesion molecule that may be associated with facilitation of ongoing inflammation during autoimmunity |
| *CCDC148* | 159,027,869 | 159,313,265 | coiled-coil domain containing 148 | Strong LD |
| *DAPL1* | 159,360,086 | 159,380,742 | Death associated protein 1 | Related to apoptosis function (defective apoptosis is a feature of SLE) |
| *BAZ2B* | 159,883,735 | 160,181,304 | Bromodomain adjacent to zinc finger | Related to transcriptional regulation |
| ***IFIH1*** | **162,831,834** | **162,883,284** | **Interferon-induced helicase 1** | **Related to interferon and apoptosis, strong candidate for type 1 diabetes** |
| *KCNH7* | 162,936,162 | 163,403,485 | Voltage-gated potassium channel, subfamily H (eag-related) | Related to signal transduction, suggestive association with multiple sclerosis |
