# Supplementary material for: Admixture Mapping in Lupus Identifies Multiple Functional Variants within IFIH1 Associated with Apoptosis, Inflammation, and Autoantibody Production
Source: PLoS Genet. 2013 Feb 18;9(2):e1003222. doi: 10.1371/journal.pgen.1003222 (PMC3575474; doi:10.1371/journal.pgen.1003222)
Supplement: Table S4 — Follow-up case-control association results. Case-control association results for the follow-up study at selected candidate genes from the strongest admixture peak (2q22–q24) using 1525 cases and 1810 controls from CCAA and 3968 cases and 3542 controls from CCEA pinpoint IFIH1 as a candidate gene for SLE. * Allele frequencies given correspond to the A1 allele. Local ancestry correction is labeled as Pc, whereas P-value refers to the uncorrected value. (DOCX) [file pgen.1003222.s010.docx]

**Table S4. Follow-up case-control association results.** Case-control association results for the follow-up study at selected candidate genes from the strongest admixture peak (2q22-q24) using 1525 cases and 1810 controls from CC_AA_ and 3968 cases and 3542 controls from CC_EA_ pinpoint IFIH1 as a candidate gene for SLE. * Allele frequencies given correspond to the A1 allele. Local ancestry correction is labeled as Pc, whereas P-value refers to the uncorrected value.

| **SNP** | **Gene** | **Function** | **Position** | **A1/A2** |  | | **CC_AA_** | | | |  | | | **CC_EA_** | | |
| --- | --- | --- | --- | --- | --- | --- | --- | --- | --- | --- | --- | --- | --- | --- | --- | --- |
|  |  |  |  |  | **Case Freq.*** | **Control**  **Freq.*** | | **P-value** | **Pc** | **OR (95%CI)** | **Case** | **Control** | **P-value** | | **Pc** | **OR (95%CI)** |
|  |  |  |  |  |  |  |  |  |  |  | **Freq.*** | **Freq.*** |  |  |  |  |
| rs354706 | *ARHGAP15* | near-gene-5 | 143,602,686 | A/G | 0.332 | 0.31 | | 5.82x10^-2^ | 1.45x10^-1^ | 1.11  (1-1.23) | 0.606 | 0.599 | 3.46x10^-1^ | | 3.39x10^-1^ | 1.03  (0.97-1.1) |
| rs354708 | *ARHGAP15* | near-gene-5 | 143,603,005 | T/A | 0.657 | 0.674 | | 1.42x10^-1^ | 2.82x10^-1^ | 0.84  (1.03-0.93) | 0.398 | 0.405 | 3.71x10^-1^ | | 3.65x10^-1^ | 0.97  (0.91-1.04) |
| rs354709 | *ARHGAP15* | near-gene-5 | 143,603,423 | A/G | 0.67 | 0.69 | | 7.85x10^-2^ | 1.88x10^-1^ | 0.82  (1.01-0.91) | 0.396 | 0.404 | 3.13x10^-1^ | | 3.11x10^-1^ | 0.97  (0.91-1.03) |
| rs4662327 | *ARHGAP15* | intron | 143,876,221 | G/A | 0.875 | 0.879 | | 5.72x10^-1^ | 8.90x10^-1^ | 0.83  (1.11-0.96) | 0.633 | 0.631 | 8.60x10^-1^ | | 8.24x10^-1^ | 1.01  (0.94-1.08) |
| rs13405986 | *ARHGAP15* | intron | 143,878,204 | A/G | 0.877 | 0.881 | | 5.37x10^-1^ | 9.38x10^-1^ | 0.82  (1.11-0.95) | 0.631 | 0.633 | 8.24x10^-1^ | | 8.55x10^-1^ | 0.99  (0.93-1.06) |
| rs10189912 | *ARHGAP15* | intron | 143,879,079 | A/G | 0.881 | 0.888 | | 3.87x10^-1^ | 8.14x10^-1^ | 0.8  (1.09-0.93) |  |  |  | |  |  |
| rs12991555 | *ARHGAP15* | intron | 143,903,986 | A/T | 0.224 | 0.23 | | 5.87x10^-1^ | 3.93x10^-1^ | 0.97  (0.86-1.09) | 0.368 | 0.371 | 7.54x10^-1^ | | 7.19x10^-1^ | 0.99  (0.93-1.06) |
| rs17230746 | *ARHGAP15* | reference | 143,911,035 | C/A | 0.943 | 0.946 | | 6.58x10^-1^ | 7.87x10^-1^ | 0.77  (1.18-0.95) | 0.781 | 0.787 | 4.28x10^-1^ | | 4.24x10^-1^ | 0.97  (0.9-1.05) |
| rs13413953 | *ARHGAP15* | intron | 143,953,506 | T/C | 0.929 | 0.936 | | 3.15x10^-1^ | 9.93x10^-1^ | 0.75  (1.1-0.91) | 0.640 | 0.638 | 7.50x10^-1^ | | 7.05x10^-1^ | 1.01  (0.95-1.08) |
| rs10048736 | *ARHGAP15* | intron | 143,955,773 | G/A | 0.809 | 0.801 | | 4.27x10^-1^ | 2.12x10^-1^ | 0.93  (1.19-1.05) | 0.625 | 0.622 | 7.23x10^-1^ | | 6.84x10^-1^ | 1.01  (0.95-1.08) |
| rs6734367 | *ARHGAP15* | intron | 144,030,717 | G/A | 0.87 | 0.858 | | 1.64x10^-1^ | 1.24x10^-1^ | 0.96  (1.28-1.11) |  |  |  | |  |  |
| rs7604660 | *ARHGAP15* | intron | 144,033,499 | A/T | 0.502 | 0.499 | | 8.41x10^-1^ | 4.83x10^-1^ | 0.92  (1.11-1.01) | 0.225 | 0.228 | 6.38x10^-1^ | | 6.84x10^-1^ | 0.98  (0.91-1.06) |
| rs4662339 | *ARHGAP15* | intron | 144,035,302 | C/A | 0.827 | 0.822 | | 5.35x10^-1^ | 5.11x10^-1^ | 0.92  (1.18-1.04) | 0.820 | 0.820 | 9.93x10^-1^ | | 9.46x10^-1^ | 1  (0.92-1.09) |
| rs4416158 | *ARHGAP15* | intron | 144,039,308 | A/G | 0.827 | 0.823 | | 6.97x10^-1^ | 6.67x10^-1^ | 0.9  (1.16-1.03) | 0.819 | 0.820 | 9.30x10^-1^ | | 8.76x10^-1^ | 1  (0.92-1.08) |
| rs7587725 | *ARHGAP15* | intron | 144,040,672 | G/A | 0.574 | 0.574 | | 9.84x10^-1^ | 7.05x10^-1^ | 0.91  (1.1-1) | 0.778 | 0.774 | 5.52x10^-1^ | | 5.91x10^-1^ | 1.02  (0.95-1.11) |
| rs4233568 | *ARHGAP15* | intron | 144,045,650 | G/A | 0.181 | 0.195 | | 1.57x10^-1^ | 1.42x10^-1^ | 0.91  (0.8-1.04) | 0.199 | 0.195 | 5.54x10^-1^ | | 4.95x10^-1^ | 1.03  (0.94-1.12) |
| rs7606634 | *ARHGAP15* | intron | 144,046,027 | C/A | 0.563 | 0.568 | | 7.16x10^-1^ | 4.48x10^-1^ | 0.89  (1.08-0.98) | 0.786 | 0.782 | 5.20x10^-1^ | | 5.56x10^-1^ | 1.03  (0.95-1.11) |
| rs10187194 | *ARHGAP15* | intron | 144,046,491 | T/A | 0.49 | 0.495 | | 7.06x10^-1^ | 3.64x10^-1^ | 0.98  (0.89-1.08) | 0.785 | 0.781 | 5.27x10^-1^ | | 5.61x10^-1^ | 1.03  (0.95-1.11) |
| rs4325696 | *ARHGAP15* | intron | 144,049,324 | T/C | 0.61 | 0.611 | | 9.41x10^-1^ | 7.64x10^-1^ | 0.9  (1.1-1) | 0.608 | 0.605 | 6.56x10^-1^ | | 7.12x10^-1^ | 1.02  (0.95-1.08) |
| rs10170378 | *ARHGAP15* | intron | 144,050,548 | T/G | 0.804 | 0.796 | | 4.14x10^-1^ | 3.90x10^-1^ | 0.93  (1.19-1.05) | 0.793 | 0.793 | 9.12x10^-1^ | | 9.77x10^-1^ | 1  (0.93-1.09) |
| rs13424620 | *ARHGAP15* | intron | 144,053,557 | G/A | 0.322 | 0.32 | | 8.99x10^-1^ | 6.76x10^-1^ | 1.01  (0.91-1.12) | 0.609 | 0.606 | 7.24x10^-1^ | | 7.98x10^-1^ | 1.01  (0.95-1.08) |
| rs7563343 | *ARHGAP15* | intron | 144,053,615 | T/C | 0.55 | 0.555 | | 6.63x10^-1^ | 8.61x10^-1^ | 0.89  (1.08-0.98) | 0.785 | 0.782 | 6.75x10^-1^ | | 7.20x10^-1^ | 1.02  (0.94-1.1) |
| rs10171904 | *ARHGAP15* | intron | 144,099,111 | A/G | 0.675 | 0.674 | | 9.19x10^-1^ | 5.81x10^-1^ | 0.91  (1.11-1.01) | 0.399 | 0.402 | 7.14x10^-1^ | | 7.83x10^-1^ | 0.99  (0.93-1.06) |
| rs13393509 | *ARHGAP15* | intron | 144,099,874 | A/C | 0.949 | 0.945 | | 5.51x10^-1^ | 7.11x10^-1^ | 0.86  (1.33-1.07) | 1.000 | 1.000 | 9.38x10^-1^ | | 9.46x10^-1^ | 1.12  (0.07-17.86) |
| rs1517914 | *ARHGAP15* | intron | 144,117,018 | A/G | 0.509 | 0.508 | | 9.64x10^-1^ | 5.11x10^-1^ | 0.91  (1.1-1) | 0.912 | 0.911 | 7.97x10^-1^ | | 6.93x10^-1^ | 1.01  (0.91-1.14) |
| rs12691685 | *ARHGAP15* | intron | 144,120,119 | A/G | 0.53 | 0.533 | | 8.01x10^-1^ | 3.65x10^-1^ | 0.9  (1.09-0.99) | 0.916 | 0.916 | 9.33x10^-1^ | | 8.21x10^-1^ | 1.01  (0.9-1.13) |
| rs12691687 | *ARHGAP15* | intron | 144,121,718 | C/A | 0.526 | 0.532 | | 6.36x10^-1^ | 2.65x10^-1^ | 0.89  (1.08-0.98) | 0.916 | 0.916 | 9.61x10^-1^ | | 8.65x10^-1^ | 1  (0.89-1.13) |
| rs13430960 | *ARHGAP15* | intron | 144,126,419 | A/G | 0.766 | 0.776 | | 3.27x10^-1^ | 3.85x10^-1^ | 0.84  (1.06-0.94) | 0.705 | 0.701 | 5.74x10^-1^ | | 4.85x10^-1^ | 1.02  (0.95-1.09) |
| rs13431916 | *ARHGAP15* | intron | 144,127,787 | T/C | 0.932 | 0.931 | | 9.03x10^-1^ | 8.97x10^-1^ | 0.84  (1.23-1.01) | 1.000 | 1.000 | 2.90x10^-1^ | | 9.99x10^-1^ |  |
| rs10211220 | *ARHGAP15* | intron | 144,131,629 | T/G | 0.539 | 0.543 | | 7.40x10^-1^ | 4.25x10^-1^ | 0.89  (1.08-0.98) | 0.839 | 0.840 | 8.20x10^-1^ | | 9.73x10^-1^ | 0.99  (0.91-1.08) |
| rs6750487 | *ARHGAP15* | intron | 144,132,375 | G/A | 0.888 | 0.885 | | 6.22x10^-1^ | 6.18x10^-1^ | 0.89  (1.21-1.04) | 0.866 | 0.867 | 9.12x10^-1^ | | 8.01x10^-1^ | 1  (0.9-1.09) |
| rs4146024 | *ARHGAP15* | intron | 144,133,632 | A/G | 0.014 | 0.019 | | 7.44x10^-2^ | 2.36x10^-2^ | 0.71  (0.48-1.04) | 0.070 | 0.069 | 8.21x10^-1^ | | 7.55x10^-1^ | 1.02  (0.89-1.15) |
| rs10171182 | *ARHGAP15* | intron | 144,134,053 | G/C | 0.208 | 0.211 | | 7.40x10^-1^ | 8.50x10^-1^ | 0.98  (0.87-1.1) | 0.153 | 0.151 | 7.29x10^-1^ | | 6.20x10^-1^ | 1.02  (0.93-1.11) |
| rs1980207 | *ARHGAP15* | intron | 144,144,180 | G/C | 0.835 | 0.835 | | 9.46x10^-1^ | 8.56x10^-1^ | 0.87  (1.13-1) | 0.685 | 0.686 | 8.90x10^-1^ | | 7.90x10^-1^ | 1  (0.93-1.07) |
| rs12691689 | *ARHGAP15* | intron | 144,144,756 | C/A | 0.942 | 0.942 | | 9.53x10^-1^ | 4.06x10^-1^ | 0.81  (1.22-0.99) | 0.693 | 0.695 | 7.91x10^-1^ | | 6.94x10^-1^ | 0.99  (0.92-1.06) |
| rs10928195 | *ARHGAP15* | intron | 144,145,078 | G/C | 0.586 | 0.587 | | 9.53x10^-1^ | 6.30x10^-1^ | 0.9 (1.1-1) | 0.864 | 0.861 | 6.31x10^-1^ | | 4.98x10^-1^ | 1.02  (0.93-1.12) |
| rs1517912 | *ARHGAP15* | intron | 144,146,240 | T/G | 0.52 | 0.519 | | 9.42x10^-1^ | 5.85x10^-1^ | 0.91  (1.11-1) | 0.879 | 0.878 | 8.72x10^-1^ | | 7.54x10^-1^ | 1.01  (0.91-1.11) |
| rs13391855 | *ARHGAP15* | intron | 144,147,300 | T/A | 0.154 | 0.17 | | 6.42x10^-2^ | 1.51x10^-1^ | 0.88  (0.77-1.01) | 0.001 | 0.000 | 4.07x10^-1^ | | 4.06x10^-1^ | 1.78  (0.45-7.14) |
| rs1517911 | *ARHGAP15* | intron | 144,151,489 | T/G | 0.562 | 0.564 | | 8.65x10^-1^ | 3.27x10^-1^ | 0.9  (1.09-0.99) | 0.987 | 0.988 | 6.58x10^-1^ | | 6.67x10^-1^ | 0.94  (0.7-1.26) |
| rs4662212 | *ARHGAP15* | intron | 144,155,311 | A/G | 0.961 | 0.962 | | 8.96x10^-1^ | 7.84x10^-1^ | 0.77  (1.26-0.98) | 0.998 | 0.997 | 4.95x10^-1^ | | 5.14x10^-1^ | 1.25  (0.66-2.36) |
| rs13382860 | *ARHGAP15* | intron | 144,156,776 | A/C | 0.305 | 0.302 | | 8.18x10^-1^ | 9.19x10^-1^ | 1.01  (0.91-1.12) | 0.353 | 0.356 | 6.48x10^-1^ | | 4.53x10^-1^ | 0.98  (0.92-1.05) |
| rs6708276 | *ARHGAP15* | intron | 144,158,620 | G/A | 0.321 | 0.322 | | 9.21x10^-1^ | 8.41x10^-1^ | 0.99  (0.9-1.1) | 0.342 | 0.344 | 7.50x10^-1^ | | 5.43x10^-1^ | 0.99 (0.92-1.06) |
| rs9653185 | *ARHGAP15* | intron | 144,158,746 | G/A | 0.986 | 0.986 | | 8.16x10^-1^ | 7.17x10^-1^ | 0.63  (1.44-0.95) | 1.000 | 0.998 | **1.07x10^-2^** | | 2.09x10^-2^ | 4.49  (1.27-15.93) |
| rs2381553 | *ARHGAP15* | intron | 144,162,450 | T/G | 0.676 | 0.677 | | 9.37x10^-1^ | 9.73x10^-1^ | 0.9  (1.1-1) | 0.630 | 0.626 | 6.68x10^-1^ | | 4.73x10^-1^ | 1.01  (0.95-1.08) |
| rs1996447 | *ARHGAP15* | intron | 144,166,651 | T/C | 0.934 | 0.933 | | 9.36x10^-1^ | 2.85x10^-1^ | 0.83  (1.22-1.01) | 0.639 | 0.636 | 7.22x10^-1^ | | 8.39x10^-1^ | 1.01  (0.95-1.08) |
| rs16822931 | *ARHGAP15* | intron | 144,166,762 | G/A | 0.906 | 0.901 | | 5.34x10^-1^ | 3.58x10^-1^ | 0.89  (1.24-1.05) | 0.822 | 0.829 | 3.01x10^-1^ | | 2.30x10^-1^ | 0.96  (0.88-1.04) |
| rs10928196 | *ARHGAP15* | intron | 144,169,030 | T/G | 0.968 | 0.961 | | 1.42x10^-1^ | 7.76x10^-2^ | 0.93  (1.6-1.22) | 0.919 | 0.920 | 7.50x10^-1^ | | 7.43x10^-1^ | 0.98  (0.87-1.11) |
| rs10173104 | *ARHGAP15* | intron | 144,169,520 | G/A | 0.877 | 0.87 | | 3.93x10^-1^ | 6.38x10^-1^ | 0.92  (1.23-1.07) | 0.999 | 1.000 | 4.97x10^-1^ | | 4.89x10^-1^ | 0.56  (0.1-3.06) |
| rs11688898 | *ARHGAP15* | intron | 144,176,808 | G/A | 0.991 | 0.99 | | 6.23x10^-1^ | 4.48x10^-1^ | 0.68  (1.89-1.14) | 0.949 | 0.946 | 5.15x10^-1^ | | 6.32x10^-1^ | 1.05 (0.91-1.21) |
| rs10173341 | *ARHGAP15* | intron | 144,177,213 | C/A | 0.638 | 0.643 | | 6.80x10^-1^ | 3.03x10^-1^ | 0.88  (1.08-0.98) | 0.990 | 0.989 | 6.60x10^-1^ | | 6.45x10^-1^ | 1.07 (0.78-1.48) |
| rs17775308 | *ARHGAP15* | intron | 144,177,809 | C/A | 0.998 | 0.999 | | 3.40x10^-1^ | 3.70x10^-1^ | 0.12  (2.11-0.5) | 0.996 | 0.996 | 9.47x10^-1^ | | 8.86x10^-1^ | 1.02  (0.62-1.67) |
| rs13401442 | *ARHGAP15* | intron | 144,177,951 | A/G | 0.887 | 0.875 | | 1.34x10^-1^ | 2.39x10^-1^ | 0.97  (1.3-1.12) | 0.999 | 1.000 | 8.21x10^-1^ | | 7.98x10^-1^ | 0.84  (0.19-3.76) |
| rs13408590 | *ARHGAP15* | intron | 144,179,745 | G/A | 0.991 | 0.992 | | 7.13x10^-1^ | 9.68x10^-1^ | 0.54  (1.53-0.91) | 0.949 | 0.954 | 1.96x10^-1^ | | 2.39x10^-1^ | 0.9  (0.78-1.05) |
| rs2381506 | *ARHGAP15* | intron | 144,181,284 | G/A | 0.999 | 0.999 | | 8.64x10^-1^ | 9.64x10^-1^ | 0.12  (5.98-0.84) | 0.996 | 0.994 | 2.85x10^-1^ | | 2.68x10^-1^ | 1.29  (0.81-2.04) |
| rs10198845 | *ARHGAP15* | intron | 144,182,557 | G/C | 0.068 | 0.077 | | 1.69x10^-1^ | 2.54x10^-2^ | 0.87  (0.72-1.06) | 0.363 | 0.362 | 9.22x10^-1^ | | 8.58x10^-1^ | 1  (0.94-1.08) |
| rs7560928 | *ARHGAP15* | intron | 144,183,642 | G/A | 0.599 | 0.6 | | 9.42x10^-1^ | 4.28x10^-1^ | 0.9  (1.1-1) | 0.988 | 0.989 | 6.24x10^-1^ | | 6.35x10^-1^ | 0.93  (0.68-1.26) |
| rs2381507 | *ARHGAP15* | intron | 144,184,229 | G/C | 0.832 | 0.848 | | 8.97x10^-2^ | 2.48x10^-1^ | 0.78  (1.02-0.89) | 0.648 | 0.644 | 5.52x10^-1^ | | 3.38x10^-1^ | 1.02  (0.95-1.09) |
| rs6757588 | *ARHGAP15* | intron | 144,184,693 | T/G | 0.899 | 0.915 | | **3.09x10^-2^** | 2.09x10^-1^ | 0.71  (0.98-0.83) | 0.644 | 0.637 | 3.52x10^-1^ | | 2.07x10^-1^ | 1.03  (0.97-1.1) |
| rs7565663 | *ARHGAP15* | intron | 144,185,437 | A/T | 0.955 | 0.947 | | 1.46x10^-1^ | 2.33x10^-1^ | 0.94  (1.48-1.18) | 0.999 | 0.999 | 6.34x10^-1^ | | 6.38x10^-1^ | 1.28  (0.46-3.53) |
| rs10164854 | *ARHGAP15* | intron | 144,186,557 | A/G | 0.11 | 0.121 | | 1.63x10^-1^ | 2.81x10^-1^ | 0.9  (0.77-1.05) | 0.000 | 0.000 | 9.10x10^-1^ | | 9.22x10^-1^ | 0.89  (0.13-6.34) |
| rs10165124 | *ARHGAP15* | intron | 144,186,762 | G/C | 0.103 | 0.113 | | 2.31x10^-1^ | 3.69x10^-1^ | 0.91  (0.78-1.06) | 0.000 | 0.000 | 9.08x10^-1^ | | 9.25x10^-1^ | 0.89  (0.13-6.33) |
| rs12465492 | *ARHGAP15* | intron | 144,193,665 | A/G | 0.178 | 0.177 | | 9.15x10^-1^ | 6.67x10^-1^ | 1.01  (0.89-1.14) | 0.377 | 0.384 | 3.30x10^-1^ | | 1.91x10^-1^ | 0.97  (0.9-1.03) |
| rs7606067 | *ARHGAP15* | intron | 144,194,221 | A/G | 0.303 | 0.296 | | 5.26x10^-1^ | 9.32x10^-1^ | 1.04  (0.93-1.15) | 0.528 | 0.536 | 3.10x10^-1^ | | 2.24x10^-1^ | 0.97  (0.91-1.03) |
| rs11890082 | *ARHGAP15* | intron | 144,197,747 | T/G | 0.982 | 0.981 | | 8.86x10^-1^ | 9.84x10^-1^ | 0.72  (1.47-1.03) | 1.000 | 1.000 | 2.90x10^-1^ | | 9.99x10^-1^ |  |
| rs16822957 | *ARHGAP15* | intron | 144,198,031 | C/A | 0.997 | 0.998 | | 4.02x10^-1^ | 4.36x10^-1^ | 0.27  (1.71-0.67) | 0.997 | 0.999 | **1.24x10^-3^** | | 3.76x10^-3^ | 0.23  (0.09-0.61) |
| rs11896630 | *ARHGAP15* | intron | 144,198,724 | A/G | 0.991 | 0.991 | | 9.99x10^-1^ | 8.84x10^-1^ | 0.59  (1.69-1) | 1.000 | 1.000 |  | |  |  |
| rs11893109 | *ARHGAP15* | intron | 144,199,898 | T/C | 0.995 | 0.993 | | 3.21x10^-1^ | 3.15x10^-1^ | 0.73  (2.56-1.37) | 1.000 | 1.000 |  | |  |  |
| rs10189240 | *ARHGAP15* | intron | 144,200,963 | T/G | 0.842 | 0.846 | | 6.57x10^-1^ | 9.01x10^-1^ | 0.85  (1.11-0.97) | 0.634 | 0.625 | 2.71x10^-1^ | | 1.69x10^-1^ | 1.04  (0.97-1.11) |
| rs2292834 | *ARHGAP15* | intron | 144,202,639 | T/G | 0.945 | 0.947 | | 7.61x10^-1^ | 7.14x10^-1^ | 0.78  (1.2-0.97) | 0.952 | 0.955 | 3.74x10^-1^ | | 3.87x10^-1^ | 0.93  (0.8-1.09) |
| rs2292833 | *ARHGAP15* | intron | 144,202,711 | A/G | 0.999 | 0.999 | | 7.97x10^-1^ | 7.75x10^-1^ | 0.21  (7.57-1.26) | 1.000 | 1.000 |  | |  |  |
| rs16822962 | *ARHGAP15* | intron | 144,205,606 | G/A | 0.892 | 0.883 | | 2.38x10^-1^ | 1.42x10^-1^ | 0.94  (1.28-1.1) | 0.808 | 0.810 | 7.47x10^-1^ | | 6.19x10^-1^ | 0.99  (0.91-1.07) |
| rs4146023 | *ARHGAP15* | intron | 144,205,718 | A/G | 0.971 | 0.968 | | 4.10x10^-1^ | 5.06x10^-1^ | 0.85  (1.49-1.13) | 0.997 | 0.996 | 4.92x10^-1^ | | 5.18x10^-1^ | 1.21  (0.7-2.09) |
| rs13005371 | *ARHGAP15* | intron | 144,207,373 | C/A | 0.934 | 0.934 | | 9.66x10^-1^ | 2.62x10^-1^ | 0.83  (1.22-1) | 0.621 | 0.622 | 9.00x10^-1^ | | 7.66x10^-1^ | 1  (0.93-1.06) |
| rs10169606 | *ARHGAP15* | intron | 144,209,384 | C/G | 0.85 | 0.857 | | 4.19x10^-1^ | 8.66x10^-1^ | 0.82  (1.09-0.94) |  |  |  | |  |  |
| rs12619951 | *ARHGAP15* | intron | 144,210,131 | A/G | 0.951 | 0.954 | | 6.89x10^-1^ | 6.70x10^-1^ | 0.76  (1.2-0.96) | 0.952 | 0.956 | 2.62x10^-1^ | | 2.71x10^-1^ | 0.92  (0.79-1.07) |
| rs10496952 | *ARHGAP15* | intron | 144,211,229 | G/A | 0.933 | 0.932 | | 9.45x10^-1^ | 2.70x10^-1^ | 0.83  (1.22-1.01) | 0.622 | 0.622 | 9.26x10^-1^ | | 9.53x10^-1^ | 1  (0.94-1.07) |
| rs13001389 | *ARHGAP15* | intron | 144,213,177 | T/C | 0.918 | 0.917 | | 8.69x10^-1^ | 3.03x10^-1^ | 0.85  (1.21-1.01) | 0.619 | 0.620 | 9.39x10^-1^ | | 8.13x10^-1^ | 1  (0.93-1.07) |
| rs12691691 | *ARHGAP15* | intron | 144,215,177 | C/T | 0.65 | 0.661 | | 3.47x10^-1^ | 8.84x10^-1^ | 0.86  (1.05-0.95) | 0.796 | 0.803 | 2.91x10^-1^ | | 2.16x10^-1^ | 0.96 (0.88-1.04) |
| rs11687380 | *ARHGAP15* | intron | 144,217,523 | T/G | 0.93 | 0.931 | | 8.70x10^-1^ | 3.84x10^-1^ | 0.81  (1.19-0.98) | 0.617 | 0.617 | 9.92x10^-1^ | | 8.84x10^-1^ | 1  (0.94-1.07) |
| rs12616872 | *ARHGAP15* | intron | 144,218,509 | G/A | 0.963 | 0.969 | | 1.68x10^-1^ | 1.80x10^-1^ | 0.63  (1.08-0.83) | 0.960 | 0.963 | 4.06x10^-1^ | | 4.38x10^-1^ | 0.93  (0.79-1.1) |
| rs12986858 | *ARHGAP15* | intron | 144,219,915 | A/G | 0.184 | 0.173 | | 2.76x10^-1^ | 5.22x10^-1^ | 1.07  (0.95-1.22) | 0.420 | 0.417 | 7.53x10^-1^ | | 6.49x10^-1^ | 1.01  (0.95-1.08) |
| rs16822986 | *ARHGAP15* | intron | 144,220,164 | T/G | 0.971 | 0.968 | | 4.87x10^-1^ | 1.49x10^-1^ | 0.83  (1.47-1.11) | 0.866 | 0.860 | 2.64x10^-1^ | | 2.00x10^-1^ | 1.05  (0.96-1.16) |
| rs1712957 | *ARHGAP15* | intron | 144,221,668 | G/A | 0.356 | 0.348 | | 4.62x10^-1^ | 7.78x10^-1^ | 1.04  (0.94-1.15) | 0.570 | 0.569 | 9.42x10^-1^ | | 5.72x10^-1^ | 1  (0.94-1.07) |
| rs938817 | *ARHGAP15* | intron | 144,222,160 | T/G | 0.846 | 0.839 | | 4.53x10^-1^ | 4.04x10^-1^ | 0.92  (1.2-1.05) | 0.827 | 0.826 | 8.13x10^-1^ | | 9.97x10^-1^ | 1.01  (0.93-1.1) |
| rs12474666 | *ARHGAP15* | intron | 144,222,498 | T/A | 0.812 | 0.816 | | 6.75x10^-1^ | 6.39x10^-1^ | 0.86  (1.1-0.97) | 0.839 | 0.842 | 5.67x10^-1^ | | 4.04x10^-1^ | 0.97  (0.89-1.06) |
| rs938816 | *ARHGAP15* | intron | 144,224,940 | A/T | 0.121 | 0.124 | | 6.94x10^-1^ | 6.01x10^-1^ | 0.97  (0.84-1.13) | 0.158 | 0.154 | 4.66x10^-1^ | | 3.06x10^-1^ | 1.03  (0.95-1.13) |
| rs6745506 | *ARHGAP15* | intron | 144,225,895 | C/A | 0.876 | 0.876 | | 9.83x10^-1^ | 9.26x10^-1^ | 0.86  (1.16-1) | 0.838 | 0.842 | 5.02x10^-1^ | | 3.55x10^-1^ | 0.97  (0.89-1.06) |
| rs10208710 | *ARHGAP15* | intron | 144,226,352 | G/A | 0.9 | 0.884 | | **3.62x10^-2^** | 7.41x10^-2^ | 1.01  (1.38-1.18) | 1.000 | 1.000 | 8.90x10^-1^ | | 9.02x10^-1^ | 1.12  (0.23-5.55) |
| rs11898334 | *ARHGAP15* | intron | 144,227,699 | T/G | 0.506 | 0.512 | | 6.50x10^-1^ | 5.89x10^-1^ | 0.89  (1.08-0.98) | 0.547 | 0.543 | 6.00x10^-1^ | | 9.12x10^-1^ | 1.02 (0.95-1.09) |
| rs9653186 | *ARHGAP15* | intron | 144,228,854 | C/G | 0.895 | 0.879 | | 5.23x10^-2^ | 1.01x10^-1^ | 1  (1.36-1.16) | 0.999 | 1.000 | 4.05x10^-1^ | | 4.03x10^-1^ | 0.56  (0.14-2.24) |
| rs16822998 | *ARHGAP15* | intron | 144,229,068 | G/C | 0.974 | 0.978 | | 2.78x10^-1^ | 2.13x10^-1^ | 0.61  (1.15-0.84) | 1.000 | 1.000 | 9.36x10^-1^ | | 9.55x10^-1^ | 1.12  (0.07-17.91) |
| rs2030215 | *ARHGAP15* | intron | 144,230,987 | G/A | 0.998 | 0.996 | | 1.86x10^-1^ | 1.87x10^-1^ | 0.74  (4.46-1.82) | 0.989 | 0.990 | 4.66x10^-1^ | | 4.82x10^-1^ | 0.89  (0.65-1.22) |
| rs1517915 | *ARHGAP15* | intron | 144,233,539 | A/G | 0.595 | 0.587 | | 5.21x10^-1^ | 6.60x10^-1^ | 0.94  (1.14-1.03) | 0.722 | 0.714 | 2.71x10^-1^ | | 3.96x10^-1^ | 1.04  (0.97-1.12) |
| rs13391674 | *ARHGAP15* | intron | 144,234,744 | G/A | 0.933 | 0.945 | | **3.26x10^-2^** | 2.07x10^-2^ | 0.66  (0.98-0.8) | 0.994 | 0.998 | **8.63x10^-5^** | | 1.91x10^-4^ | 0.31  (0.17-0.58) |
| rs13392176 | *ARHGAP15* | intron | 144,235,198 | A/G | 0.07 | 0.059 | | 7.23x10^-2^ | 4.54x10^-2^ | 1.2  (0.98-1.46) | 0.000 | 0.000 | 7.50x10^-1^ | | 7.38x10^-1^ | 1.34  (0.22-8) |
| rs1712956 | *ARHGAP15* | intron | 144,235,386 | G/A | 0.998 | 0.996 | | **4.36x10^-2^** | 4.26x10^-2^ | 0.99  (7.39-2.7) | 0.989 | 0.991 | 3.86x10^-1^ | | 3.95x10^-1^ | 0.87  (0.63-1.2) |
| rs16823000 | *ARHGAP15* | intron | 144,236,204 | A/G | 0.807 | 0.805 | | 8.45x10^-1^ | 7.83x10^-1^ | 0.9  (1.14-1.01) | 0.998 | 1.000 | **2.27x10^-2^** | | 3.41x10^-2^ | 0.26  (0.07-0.91) |
| rs6740197 | *ARHGAP15* | intron | 144,237,111 | A/G | 0.713 | 0.733 | | 7.57x10^-2^ | 4.77x10^-2^ | 0.81  (1.01-0.91) | 0.783 | 0.778 | 4.65x10^-1^ | | 4.68x10^-1^ | 1.03  (0.95-1.11) |
| rs7568511 | *ARHGAP15* | intron | 144,240,156 | T/C | 0.864 | 0.855 | | 2.92x10^-1^ | 2.97x10^-1^ | 0.94  (1.24-1.08) | 0.843 | 0.840 | 7.21x10^-1^ | | 8.98x10^-1^ | 1.02  (0.93-1.11) |
| rs7581136 | *ARHGAP15* | intron | 144,240,342 | C/A | 0.426 | 0.449 | | 6.83x10^-2^ | 1.91x10^-1^ | 0.91  (0.83-1.01) | 0.203 | 0.211 | 2.30x10^-1^ | | 2.80x10^-1^ | 0.95  (0.88-1.03) |
| rs16855324 | *ZEB2* | intron | 144,864,228 | C/A | 0.678 | 0.67 | | 4.77x10^-1^ | 6.87x10^-1^ | 0.94  (1.15-1.04) | 0.814 | 0.821 | 2.59x10^-1^ | | 2.60x10^-1^ | 0.95  (0.88-1.04) |
| rs10185359 | *ZEB2* | intron | 144,872,201 | C/T | 0.533 | 0.561 | | **2.02x10^-2^** | 8.75x10^-2^ | 0.81  (0.98-0.89) | 0.768 | 0.781 | 7.02x10^-2^ | | 7.13x10^-2^ | 0.93  (0.86-1.01) |
| rs34890427 | *ZEB2* | coding-synonymous | 144,873,814 | T/A | 0.991 | 0.991 | | 8.97x10^-1^ | 8.45x10^-1^ | 0.58  (1.62-0.97) | 1.000 | 1.000 |  | |  |  |
| rs6711223 | *ZEB2* | reference | 144,874,294 | G/A | 0.969 | 0.967 | | 6.92x10^-1^ | 8.10x10^-1^ | 0.8  (1.39-1.06) | 0.998 | 0.998 | 7.48x10^-1^ | | 7.58x10^-1^ | 1.12  (0.56-2.24) |
| rs12618034 | *ZEB2* | intron | 144,876,515 | C/A | 1 | 0.999 | | 2.46x10^-1^ | 3.80x10^-1^ | 0.38  (30.35-3.39) | 1.000 | 1.000 | 3.44x10^-1^ | | 9.99x10^-1^ |  |
| rs34961586 | *ZEB2* | coding-synonymous | 144,877,962 | G/C | 0.001 | 0 | | 4.68x10^-1^ | 5.08x10^-1^ | 2.37  (0.21-26.11) | 0.001 | 0.001 | 5.28x10^-1^ | | 5.46x10^-1^ | 1.43  (0.47-4.38) |
| rs10928370 | *ACVR2A* | intron | 148,390,035 | T/G | 0.671 | 0.648 | | **4.46x10^-2^** | 1.61x10^-1^ | 1  (1.23-1.11) | 0.985 | 0.987 | 2.07x10^-1^ | | 1.94x10^-1^ | 0.84  (0.63-1.1) |
| rs12105745 | *ACVR2A* | intron | 148,390,132 | G/A | 0.634 | 0.646 | | 3.30x10^-1^ | 4.43x10^-1^ | 0.86  (1.05-0.95) | 0.471 | 0.482 | 1.94x10^-1^ | | 2.10x10^-1^ | 0.96  (0.9-1.02) |
| rs13026650 | *ACVR2A* | intron | 148,390,671 | G/A | 0.696 | 0.707 | | 3.59x10^-1^ | 3.33x10^-1^ | 0.86  (1.06-0.95) | 0.682 | 0.691 | 2.53x10^-1^ | | 2.52x10^-1^ | 0.96  (0.9-1.03) |
| rs7601098 | *ACVR2A* | intron | 148,393,084 | G/C | 0.657 | 0.636 | | 7.04x10^-2^ | 2.02x10^-1^ | 0.99  (1.21-1.1) | 0.927 | 0.930 | 4.44x10^-1^ | | 4.47x10^-1^ | 0.95  (0.84-1.08) |
| rs16828024 | *ACVR2A* | intron | 148,393,603 | G/A | 0.091 | 0.094 | | 6.36x10^-1^ | 8.44x10^-1^ | 0.96  (0.81-1.14) | 0.013 | 0.011 | 3.40x10^-1^ | | 3.22x10^-1^ | 1.16  (0.86-1.56) |
| rs3820716 | *ACVR2A* | intron | 148,396,730 | T/C | 0.635 | 0.641 | | 6.27x10^-1^ | 7.65x10^-1^ | 0.88  (1.08-0.97) | 0.532 | 0.520 | 1.64x10^-1^ | | 1.80x10^-1^ | 1.05  (0.98-1.12) |
| rs2303392 | *ACVR2A* | intron | 148,396,897 | G/C | 0.305 | 0.294 | | 2.99x10^-1^ | 2.80x10^-1^ | 1.06  (0.95-1.18) | 0.322 | 0.311 | 1.67x10^-1^ | | 1.67x10^-1^ | 1.05  (0.98-1.13) |
| rs3771884 | *NMI* | utr-3 | 151,835,407 | C/A | 0.83 | 0.825 | | 6.11x10^-1^ | 9.76x10^-1^ | 0.91  (1.17-1.03) | 0.997 | 0.998 | 6.09x10^-2^ | | 6.31x10^-2^ | 0.54  (0.28-1.04) |
| rs446791 | *NMI* | intron | 151,838,125 | A/G | 0.605 | 0.618 | | 2.78x10^-1^ | 9.04x10^-1^ | 0.86  (1.05-0.95) |  |  |  | |  |  |
| rs4665150 | *NMI* | intron | 151,842,395 | A/G | 0.384 | 0.377 | | 5.32x10^-1^ | 7.55x10^-1^ | 1.03  (0.93-1.14) | 0.448 | 0.439 | 3.03x10^-1^ | | 2.86x10^-1^ | 1.04  (0.97-1.1) |
| rs11551174 | *NMI* | coding-synonymous | 151,843,661 | G/A | 0.943 | 0.95 | | 1.95x10^-1^ | 2.28x10^-1^ | 0.7  (1.08-0.87) | 0.938 | 0.943 | 1.95x10^-1^ | | 1.87x10^-1^ | 0.91  (0.8-1.05) |
| rs289831 | *NMI* | intron | 151,843,760 | T/G | 0.652 | 0.644 | | 4.85x10^-1^ | 9.44x10^-1^ | 0.94  (1.15-1.04) | 0.883 | 0.874 | 8.33x10^-2^ | | 8.00x10^-2^ | 1.09  (0.99-1.21) |
| rs3856557 | *NMI* | intron | 151,845,207 | C/A | 0.634 | 0.637 | | 7.82x10^-1^ | 9.58x10^-1^ | 0.89  (1.09-0.99) | 0.558 | 0.566 | 3.47x10^-1^ | | 3.39x10^-1^ | 0.97  (0.91-1.04) |
| rs3854012 | *NMI* | intron | 151,845,352 | A/G | 0.612 | 0.616 | | 7.52x10^-1^ | 9.64x10^-1^ | 0.89  (1.09-0.98) | 0.553 | 0.556 | 6.68x10^-1^ | | 6.40x10^-1^ | 0.99  (0.92-1.05) |
| rs289828 | *NMI* | intron | 151,845,427 | A/G | 0.869 | 0.87 | | 9.65x10^-1^ | 4.56x10^-1^ | 0.86  (1.15-1) | 0.607 | 0.602 | 5.81x10^-1^ | | 5.60x10^-1^ | 1.02  (0.95-1.09) |
| rs6757821 | *NMI* | intron | 151,846,283 | A/G | 0.659 | 0.661 | | 8.62x10^-1^ | 9.47x10^-1^ | 0.9  (1.1-0.99) | 0.580 | 0.579 | 9.22x10^-1^ | | 8.93x10^-1^ | 1  (0.94-1.07) |
| rs6747782 | *NMI* | intron | 151,846,361 | A/G | 0.645 | 0.646 | | 9.61x10^-1^ | 9.72x10^-1^ | 0.9  (1.1-1) | 0.577 | 0.576 | 9.00x10^-1^ | | 8.59x10^-1^ | 1  (0.94-1.07) |
| rs3771886 | *NMI* | reference | 151,846,768 | A/G | 0.642 | 0.648 | | 6.43x10^-1^ | 7.01x10^-1^ | 0.88  (1.08-0.98) | 0.577 | 0.577 | 9.92x10^-1^ | | 9.78x10^-1^ | 1  (0.94-1.07) |
| rs3771888 | *NMI* | intron | 151,847,035 | A/G | 0.644 | 0.651 | | 5.93x10^-1^ | 6.45x10^-1^ | 0.88  (1.08-0.97) | 0.578 | 0.578 | 9.77x10^-1^ | | 9.92x10^-1^ | 1  (0.94-1.07) |
| rs6751802 | *NMI* | intron | 151,847,268 | C/A | 0.644 | 0.651 | | 5.87x10^-1^ | 6.41x10^-1^ | 0.88  (1.08-0.97) | 0.577 | 0.578 | 9.39x10^-1^ | | 9.69x10^-1^ | 1  (0.93-1.06) |
| rs10169816 | *NMI* | intron | 151,847,517 | G/A | 0.645 | 0.65 | | 6.68x10^-1^ | 7.25x10^-1^ | 0.88  (1.08-0.98) | 0.579 | 0.578 | 9.89x10^-1^ | | 9.58x10^-1^ | 1  (0.94-1.07) |
| rs1048135 | *NMI* | reference | 151,847,662 | C/A | 0.884 | 0.897 | | 1.06x10^-1^ | 9.15x10^-1^ | 0.75  (1.03-0.88) | 0.559 | 0.566 | 3.70x10^-1^ | | 3.59x10^-1^ | 0.97  (0.91-1.04) |
| rs9989812 | *NMI* | intron | 151,848,645 | A/G | 0.984 | 0.988 | | 2.09x10^-1^ | 1.70x10^-1^ | 0.51  (1.16-0.77) | 1.000 | 1.000 | 3.44x10^-1^ | | 9.99x10^-1^ |  |
| rs13383563 | *NMI* | intron | 151,851,770 | T/C | 0.612 | 0.635 | | 5.61x10^-2^ | 4.92x10^-1^ | 0.82  (1-0.91) | 0.867 | 0.861 | 2.22x10^-1^ | | 2.21x10^-1^ | 1.06  (0.97-1.16) |
| rs2113509 | *NMI* | intron | 151,852,443 | C/T | 0.526 | 0.553 | | **3.06x10^-2^** | 2.02x10^-1^ | 0.81  (0.99-0.9) | 0.855 | 0.849 | 2.96x10^-1^ | | 2.92x10^-1^ | 1.05  (0.96-1.15) |
| rs2161841 | *NMI* | intron | 151,852,567 | G/A | 0.726 | 0.716 | | 3.88x10^-1^ | 6.74x10^-1^ | 0.94  (1.17-1.05) | 0.873 | 0.865 | 1.24x10^-1^ | | 1.26x10^-1^ | 1.08  (0.98-1.19) |
| rs2278089 | *NMI* | near-gene-5 | 151,854,918 | A/C | 0.869 | 0.869 | | 9.85x10^-1^ | 4.39x10^-1^ | 0.87  (1.15-1) | 0.606 | 0.602 | 5.90x10^-1^ | | 5.68x10^-1^ | 1.02  (0.95-1.09) |
| rs2342909 |  |  | 151,918,959 | T/G | 0.873 | 0.869 | | 6.83x10^-1^ | 3.69x10^-1^ | 0.89  (1.19-1.03) |  |  |  | |  |  |
| rs3771889 | *TNFAIP6* | intron | 151,922,884 | G/A | 0.72 | 0.714 | | 5.89x10^-1^ | 9.56x10^-1^ | 0.93  (1.15-1.03) | 0.861 | 0.863 | 8.21x10^-1^ | | 8.26x10^-1^ | 0.99  (0.9-1.09) |
| rs3771891 | *TNFAIP6* | intron | 151,926,431 | T/C | 0.982 | 0.986 | | 1.16x10^-1^ | 2.16x10^-1^ | 0.5  (1.08-0.73) | 0.934 | 0.927 | 1.30x10^-1^ | | 1.31x10^-1^ | 1.1  (0.97-1.25) |
| rs16829997 | *TNFAIP6* | intron | 151,931,278 | T/C | 0.996 | 0.995 | | 3.19x10^-1^ | 3.73x10^-1^ | 0.69  (3.07-1.46) | 0.996 | 0.998 | **2.18x10^-2^** | | 2.35x10^-2^ | 0.49  (0.26-0.91) |
| rs10180428 | *TNFAIP6* | intron | 151,931,727 | C/G | 0.974 | 0.977 | | 5.81x10^-1^ | 4.60x10^-1^ | 0.67  (1.25-0.92) | 1.000 | 1.000 | 4.98x10^-1^ | | 5.10x10^-1^ | 2.24  (0.2-24.73) |
| rs3771894 | *TNFAIP6* | intron | 151,932,507 | C/A | 0.999 | 0.999 | | 6.40x10^-1^ | 6.43x10^-1^ | 0.34  (5.88-1.4) | 0.999 | 1.000 | 8.19x10^-1^ | | 8.19x10^-1^ | 0.84  (0.19-3.75) |
| rs3771895 | *TNFAIP6* | intron | 151,932,585 | A/G | 0.416 | 0.416 | | 9.93x10^-1^ | 9.71x10^-1^ | 1  (0.91-1.1) | 0.519 | 0.518 | 8.74x10^-1^ | | 8.54x10^-1^ | 1.01  (0.94-1.07) |
| rs3771898 | *TNFAIP6* | intron | 151,932,887 | T/G | 0.998 | 0.998 | | 7.65x10^-1^ | 7.78x10^-1^ | 0.27  (2.61-0.84) | 0.999 | 0.999 | 9.35x10^-1^ | | 9.41x10^-1^ | 0.96  (0.32-2.84) |
| rs10432475 | *TNFAIP6* | intron | 151,933,186 | A/G | 0.981 | 0.982 | | 7.30x10^-1^ | 8.37x10^-1^ | 0.66  (1.34-0.94) | 0.885 | 0.886 | 9.47x10^-1^ | | 9.40x10^-1^ | 1  (0.9-1.1) |
| rs10432476 | *TNFAIP6* | intron | 151,933,334 | C/T | 0.586 | 0.589 | | 7.70x10^-1^ | 7.92x10^-1^ | 0.89  (1.09-0.99) | 0.508 | 0.510 | 8.54x10^-1^ | | 8.74x10^-1^ | 0.99  (0.93-1.06) |
| rs10201647 | *TNFAIP6* | intron | 151,933,539 | T/G | 0.975 | 0.977 | | 5.96x10^-1^ | 4.73x10^-1^ | 0.67  (1.26-0.92) | 1.000 | 1.000 | 5.00x10^-1^ | | 5.11x10^-1^ | 2.24  (0.2-24.67) |
| rs1046668 | *TNFAIP6* | reference | 151,934,816 | G/A | 0.283 | 0.286 | | 7.74x10^-1^ | 8.52x10^-1^ | 0.98  (0.88-1.1) | 0.140 | 0.138 | 6.78x10^-1^ | | 6.81x10^-1^ | 1.02  (0.93-1.12) |
| rs4664031 | *TNFAIP6* | intron | 151,937,003 | A/G | 0.88 | 0.891 | | 1.68x10^-1^ | 9.88x10^-1^ | 0.77  (1.05-0.9) | 0.620 | 0.625 | 5.49x10^-1^ | | 5.77x10^-1^ | 0.98  (0.92-1.05) |
| rs13389056 | *TNFAIP6* | intron | 151,938,272 | T/G | 0.916 | 0.915 | | 8.19x10^-1^ | 8.77x10^-1^ | 0.86  (1.21-1.02) | 1.000 | 0.999 | 2.44x10^-1^ | | 2.61x10^-1^ | 2.23  (0.56-8.92) |
| rs3845844 | *TNFAIP6* | intron | 151,939,540 | A/G | 0.81 | 0.808 | | 8.12x10^-1^ | 9.95x10^-1^ | 0.9  (1.15-1.02) | 0.865 | 0.866 | 8.84x10^-1^ | | 8.89x10^-1^ | 0.99  (0.9-1.09) |
| rs1046675 | *TNFAIP6* | utr-3 | 151,944,644 | A/G | 0.807 | 0.806 | | 8.88x10^-1^ | 9.20x10^-1^ | 0.89  (1.14-1.01) | 0.860 | 0.863 | 6.43x10^-1^ | | 6.46x10^-1^ | 0.98  (0.89-1.07) |
| rs2444272 | *RIF1* | intron | 152,001,866 | A/G | 0.59 | 0.581 | | 4.34x10^-1^ | 5.62x10^-1^ | 0.94  (1.15-1.04) | 0.661 | 0.661 | 9.42x10^-1^ | | 9.51x10^-1^ | 1  (0.94-1.07) |
| rs2444263 | *RIF1* | missense | 152,019,816 | T/G | 0.596 | 0.585 | | 3.35x10^-1^ | 4.62x10^-1^ | 0.95  (1.16-1.05) | 0.668 | 0.665 | 6.59x10^-1^ | | 6.69x10^-1^ | 1.02  (0.95-1.09) |
| rs16830047 | *RIF1* | coding-synonymous | 152,022,631 | G/A | 0.851 | 0.846 | | 6.36x10^-1^ | 3.94x10^-1^ | 0.9  (1.19-1.03) |  |  |  | |  |  |
| rs2123465 | *RIF1* | missense | 152,028,364 | T/G | 0.595 | 0.58 | | 2.22x10^-1^ | 3.08x10^-1^ | 0.96  (1.17-1.06) | 0.658 | 0.655 | 6.63x10^-1^ | | 6.74x10^-1^ | 1.02  (0.95-1.09) |
| rs2444257 | *RIF1* | reference | 152,030,341 | A/T | 0.396 | 0.409 | | 2.92x10^-1^ | 4.64x10^-1^ | 0.95  (0.86-1.05) | 0.277 | 0.283 | 4.59x10^-1^ | | 4.78x10^-1^ | 0.97  (0.91-1.05) |
| rs1065177 | *RIF1* | reference | 152,039,664 | G/C | 0.598 | 0.587 | | 3.83x10^-1^ | 5.83x10^-1^ | 0.95  (1.15-1.04) | 0.722 | 0.717 | 4.67x10^-1^ | | 4.85x10^-1^ | 1.03  (0.96-1.1) |
| rs1061305 | *NEB* | reference | 152,055,225 | A/G | 0.573 | 0.57 | | 8.39x10^-1^ | 8.22x10^-1^ | 0.92  (1.11-1.01) | 0.595 | 0.599 | 6.48x10^-1^ | | 7.29x10^-1^ | 0.99  (0.92-1.05) |
| rs34368668 | *NEB* | reference | 152,071,730 | C/A | 0.966 | 0.954 | | **2.16x10^-2^** | 4.79x10^-2^ | 1.04  (1.73-1.34) | 0.996 | 0.997 | 8.73x10^-1^ | | 8.78x10^-1^ | 0.96  (0.55-1.67) |
| rs3732309 | *NEB* | missense | 152,081,220 | C/A | 1 | 0.999 | | 4.05x10^-1^ | 3.99x10^-1^ | 0.26  (24.35-2.53) | 0.999 | 0.999 | 8.71x10^-1^ | | 8.65x10^-1^ | 0.9  (0.24-3.34) |
| rs35625617 | *NEB* | missense | 152,091,767 | G/A | 0.997 | 0.995 | | 1.25x10^-1^ | 5.90x10^-2^ | 0.83  (4.38-1.9) | 0.978 | 0.978 | 9.17x10^-1^ | | 9.40x10^-1^ | 1.01  (0.81-1.26) |
| rs16830170 | *NEB* | reference | 152,096,555 | G/A | 0.971 | 0.971 | | 9.72x10^-1^ | 8.35x10^-1^ | 0.75  (1.33-1) | 0.999 | 0.999 | 6.25x10^-1^ | | 6.41x10^-1^ | 1.34  (0.41-4.4) |
| rs2288200 | *NEB* | reference | 152,112,464 | T/A | 0.113 | 0.123 | | 1.99x10^-1^ | 4.45x10^-1^ | 0.91  (0.78-1.05) | 0.004 | 0.005 | 6.48x10^-1^ | | 6.53x10^-1^ | 0.89  (0.55-1.46) |
| rs35707762 | *NEB* | reference | 152,113,147 | C/A | 0.996 | 0.998 | | 4.05x10^-1^ | 5.38x10^-1^ | 0.28  (1.66-0.69) | 0.988 | 0.987 | 6.25x10^-1^ | | 6.33x10^-1^ | 1.07  (0.81-1.43) |
| rs34471889 | *NEB* | missense | 152,128,406 | G/A | 0.994 | 0.993 | | 7.34x10^-1^ | 8.36x10^-1^ | 0.61  (2.02-1.11) | 1.000 | 1.000 |  | |  |  |
| rs34504204 | *NEB* | reference | 152,129,844 | A/0 | 1 | 1 | |  |  |  | 0.999 | 0.998 | 2.36x10^-1^ | | 2.42x10^-1^ | 1.76  (0.68-4.55) |
| rs2288210 | *NEB* | missense | 152,130,322 | G/C | 0.383 | 0.381 | | 8.50x10^-1^ | 8.28x10^-1^ | 1.01  (0.91-1.12) | 0.333 | 0.333 | 9.62x10^-1^ | | 9.40x10^-1^ | 1  (0.94-1.07) |
| rs16830236 | *NEB* | reference | 152,131,971 | T/G | 0.962 | 0.962 | | 9.62x10^-1^ | 8.08x10^-1^ | 0.77  (1.28-0.99) | 1.000 | 1.000 | 2.64x10^-1^ | | 2.89x10^-1^ | 3.36  (0.35-32.35) |
| rs35227368 | *NEB* | missense | 152,141,057 | T/A | 0.093 | 0.098 | | 5.48x10^-1^ | 9.37x10^-1^ | 0.95  (0.81-1.12) | 0.004 | 0.004 | 6.70x10^-1^ | | 6.74x10^-1^ | 0.89  (0.53-1.51) |
| rs6713162 | *NEB* | missense | 152,204,772 | G/A | 0.61 | 0.642 | | **6.53x10^-3^** | 1.48x10^-1^ | 0.79  (0.96-0.87) | 0.158 | 0.171 | **2.48x10^-2^** | | 2.73x10^-2^ | 0.91  (0.83-0.99) |
| rs35974308 | *NEB* | missense | 152,207,389 | G/A | 1 | 0.999 | | 3.88x10^-1^ | 4.18x10^-1^ | 0.27  (25.11-2.61) |  |  |  | |  |  |
| rs35555631 | *NEB* | missense | 152,207,998 | G/A | 0.996 | 0.994 | | 1.73x10^-1^ | 2.33x10^-1^ | 0.8  (3.26-1.62) | 1.000 | 1.000 |  | |  |  |
| rs34532796 | *NEB* | missense | 152,226,960 | T/G | 0.985 | 0.98 | | 1.97x10^-1^ | 2.59x10^-1^ | 0.88  (1.85-1.28) | 1.000 | 1.000 | 9.10x10^-1^ | | 9.09x10^-1^ | 1.12  (0.16-7.96) |
| rs35946547 | *NEB* | coding-synonymous | 152,228,359 | T/G | 0.999 | 0.999 | | 5.42x10^-1^ | 5.87x10^-1^ | 0.31  (9.22-1.69) | 1.000 | 1.000 |  | |  |  |
| rs10170273 | *NEB* | coding-synonymous | 152,229,342 | T/C | 0.578 | 0.608 | | **1.33x10^-2^** | 2.11x10^-1^ | 0.8  (0.97-0.88) | 0.845 | 0.828 | **4.38x10^-3^** | | 5.01x10^-3^ | 1.13  (1.04-1.24) |
| rs35016946 | *NEB* | reference | 152,230,150 | C/A | 1 | 1 | | 3.59x10^-1^ | 9.99x10^-1^ |  | 1.000 | 1.000 | 7.48x10^-1^ | | 7.54x10^-1^ | 0.75  (0.12-4.47) |
| rs7426114 | *NEB* | missense | 152,235,818 | C/T | 0.693 | 0.711 | | 1.09x10^-1^ | 8.88x10^-1^ | 0.83  (1.02-0.92) | 0.842 | 0.827 | **1.81x10^-2^** | | 2.01x10^-2^ | 1.11  (1.02-1.21) |
| rs34577613 | *NEB* | missense | 152,235,854 | A/G | 0.379 | 0.386 | | 5.72x10^-1^ | 7.33x10^-1^ | 0.97  (0.88-1.07) | 0.101 | 0.116 | **2.84x10^-3^** | | 3.30x10^-3^ | 0.85  (0.77-0.95) |
| rs34800215 | *NEB* | missense | 152,235,882 | G/C | 0.95 | 0.95 | | 9.48x10^-1^ | 7.41x10^-1^ | 0.79  (1.24-0.99) | 0.983 | 0.982 | 4.63x10^-1^ | | 4.73x10^-1^ | 1.1  (0.86-1.4) |
| rs35654397 | *NEB* | coding-synonymous | 152,237,156 | G/C | 0.001 | 0.001 | | 5.30x10^-1^ | 7.36x10^-1^ | 1.76  (0.29-10.54) | 0.004 | 0.002 | 1.63x10^-1^ | | 1.62x10^-1^ | 1.53  (0.84-2.78) |
| rs6709752 | *NEB* | coding-synonymous | 152,237,207 | C/A | 0.999 | 0.998 | | 2.16x10^-1^ | 2.43x10^-1^ | 0.6  (8.53-2.26) | 1.000 | 1.000 | 2.90x10^-1^ | | 9.99x10^-1^ |  |
| rs6711382 | *NEB* | missense | 152,239,323 | G/A | 0.643 | 0.647 | | 7.10x10^-1^ | 2.35x10^-1^ | 0.89  (1.09-0.98) | 0.903 | 0.887 | **1.07x10^-3^** | | 1.36x10^-3^ | 1.19  (1.07-1.32) |
| rs34234609 | *NEB* | missense | 152,240,100 | C/A | 0.992 | 0.99 | | 4.66x10^-1^ | 5.66x10^-1^ | 0.72  (2.05-1.22) | 0.999 | 1.000 | **2.15x10^-2^** | | 3.74x10^-2^ | 0.2  (0.05-0.92) |
| rs35194393 | *NEB* | reference | 152,244,525 | A/C | 1 | 0.999 | | 4.05x10^-1^ | 4.35x10^-1^ | 0.26  (24.34-2.53) | 1.000 | 1.000 |  | |  |  |
| rs6735208 | *NEB* | reference | 152,244,744 | T/A | 0.603 | 0.613 | | 3.67x10^-1^ | 6.26x10^-1^ | 0.87  (1.05-0.96) | 0.164 | 0.181 | **5.41x10^-3^** | | 6.25x10^-3^ | 0.89  (0.81-0.97) |
| rs12464543 | *NEB* | intron | 152,274,679 | A/G | 0.603 | 0.633 | | **1.41x10^-2^** | 2.73x10^-1^ | 0.8  (0.97-0.88) | 0.161 | 0.178 | **5.73x10^-3^** | | 7.01x10^-3^ | 0.88  (0.81-0.96) |
| rs4611637 | *NEB* | coding-synonymous | 152,282,227 | G/A | 0.373 | 0.347 | | **2.54x10^-2^** | 3.36x10^-1^ | 1.12  (1.01-1.24) | 0.842 | 0.822 | **1.03x10^-3^** | | 1.21x10^-3^ | 1.16  (1.06-1.26) |
| rs35686968 | *NEB* | missense | 152,289,061 | G/C | 0.005 | 0.003 | | 1.57x10^-1^ | 2.45x10^-1^ | 1.73  (0.8-3.73) | 0.021 | 0.028 | **6.19x10^-3^** | | 7.36x10^-3^ | 0.75  (0.61-0.92) |
| rs13410490 | *CACNB4* | utr-3 | 152,402,694 | A/G | 0.828 | 0.806 | | **2.34x10^-2^** | 7.74x10^-2^ | 1.02  (1.31-1.16) | 0.918 | 0.911 | 1.33x10^-1^ | | 1.63x10^-1^ | 1.09  (0.97-1.23) |
| rs7597215 | *CACNB4* | utr-3 | 152,403,437 | T/C | 0.823 | 0.837 | | 1.17x10^-1^ | 5.80x10^-2^ | 0.79  (1.03-0.9) | 0.819 | 0.805 | **2.61x10^-2^** | | 3.35x10^-2^ | 1.1  (1.01-1.2) |
| rs3754533 | *CACNB4* | intron | 152,405,709 | C/A | 1 | 0.999 | | 1.53x10^-1^ | 1.95x10^-1^ | 0.49  (36.09-4.21) | 1.000 | 0.999 | 2.42x10^-1^ | | 2.62x10^-1^ | 2.24  (0.56-8.96) |
| rs6755393 | *CACNB4* | intron | 152,410,283 | C/A | 0.739 | 0.747 | | 4.19x10^-1^ | 1.36x10^-1^ | 0.86  (1.07-0.96) | 0.939 | 0.931 | 5.23x10^-2^ | | 5.21x10^-2^ | 1.14  (1-1.3) |
| rs12693152 | *CACNB4* | intron | 152,423,249 | C/A | 0.878 | 0.854 | | **4.33x10^-3^** | 1.43x10^-2^ | 1.07  (1.42-1.23) | 0.918 | 0.910 | 5.85x10^-2^ | | 7.42x10^-2^ | 1.12  (1-1.25) |
| rs3768653 | *CACNB4* | intron | 152,424,874 | G/A | 0.953 | 0.947 | | 2.32x10^-1^ | 1.29x10^-2^ | 0.92  (1.43-1.14) | 0.793 | 0.794 | 9.53x10^-1^ | | 9.95x10^-1^ | 1  (0.92-1.08) |
| rs7419564 | *CACNB4* | intron | 152,428,276 | A/G | 0.709 | 0.729 | | 7.27x10^-2^ | 8.76x10^-1^ | 0.81  (1.01-0.91) | 0.174 | 0.188 | **2.66x10^-2^** | | 3.37x10^-2^ | 0.91  (0.84-0.99) |
| rs6742375 | *CACNB4* | intron | 152,444,637 | G/A | 0.805 | 0.785 | | **3.51x10^-2^** | 2.31x10^-2^ | 1.01  (1.28-1.14) | 0.740 | 0.736 | 5.72x10^-1^ | | 5.77x10^-1^ | 1.02  (0.95-1.1) |
| rs1519707 | *CACNB4* | intron | 152,445,136 | C/A | 0.803 | 0.809 | | 5.70x10^-1^ | 2.16x10^-1^ | 0.85  (1.09-0.97) | 0.999 | 0.998 | 6.30x10^-1^ | | 6.33x10^-1^ | 1.22  (0.54-2.77) |
| rs35343813 | *CACNB4* | intron | 152,512,421 | C/T | 0.848 | 0.877 | | **4.51x10^-4^** | 3.22x10^-2^ | 0.68  (0.9-0.78) | 0.614 | 0.607 | 4.05x10^-1^ | | 4.17x10^-1^ | 1.03  (0.96-1.1) |
| rs10497090 | *CACNB4* | intron | 152,512,576 | G/A | 0.79 | 0.752 | | **2.73x10^-4^** | 1.97x10^-4^ | 1.1  (1.39-1.24) | 0.728 | 0.724 | 5.80x10^-1^ | | 5.71x10^-1^ | 1.02  (0.95-1.1) |
| rs10497091 | *CACNB4* | intron | 152,513,655 | A/G | 0.789 | 0.754 | | **5.84x10^-4^** | 4.27x10^-4^ | 1.09  (1.37-1.22) | 0.729 | 0.724 | 5.00x10^-1^ | | 4.90x10^-1^ | 1.03  (0.95-1.1) |
| rs1879140 | *CACNB4* | intron | 152,564,308 | G/A | 0.615 | 0.606 | | 4.59x10^-1^ | 8.51x10^-1^ | 0.94  (1.15-1.04) | 0.954 | 0.955 | 9.30x10^-1^ | | 9.03x10^-1^ | 0.99  (0.85-1.16) |
| rs12693199 | *CACNB4* | intron | 152,568,238 | A/G | 0.629 | 0.604 | | **3.48x10^-2^** | 5.74x10^-2^ | 1.01  (1.23-1.11) | 0.651 | 0.649 | 8.06x10^-1^ | | 8.09x10^-1^ | 1.01  (0.94-1.08) |
| rs12693200 | *CACNB4* | intron | 152,568,249 | A/G | 0.834 | 0.796 | | **9.07x10^-5^** | 7.33x10^-5^ | 1.13  (1.46-1.28) | 0.770 | 0.773 | 6.64x10^-1^ | | 7.16x10^-1^ | 0.98  (0.91-1.06) |
| rs16830583 | *CACNB4* | intron | 152,570,489 | A/G | 0.695 | 0.705 | | 3.47x10^-1^ | 7.97x10^-2^ | 0.86  (1.06-0.95) | 0.998 | 0.998 | 7.47x10^-1^ | | 7.56x10^-1^ | 1.12  (0.56-2.24) |
| rs11686080 | *CACNB4* | intron | 152,572,102 | G/A | 0.829 | 0.794 | | **2.61x10^-4^** | 1.57x10^-4^ | 1.11  (1.43-1.26) | 0.734 | 0.742 | 3.06x10^-1^ | | 3.39x10^-1^ | 0.96  (0.9-1.04) |
| rs6736449 | *CACNB4* | intron | 152,578,170 | C/G | 0.745 | 0.777 | | **3.01x10^-3^** | 4.42x10^-2^ | 0.75  (0.94-0.84) | 0.438 | 0.435 | 7.11x10^-1^ | | 7.04x10^-1^ | 1.01  (0.95-1.08) |
| rs4664519 | *CACNB4* | intron | 152,580,435 | G/A | 0.903 | 0.91 | | 3.05x10^-1^ | 3.34x10^-1^ | 0.78  (1.08-0.92) | 0.893 | 0.890 | 5.62x10^-1^ | | 5.98x10^-1^ | 1.03  (0.93-1.14) |
| rs6727569 | *CACNB4* | intron | 152,581,403 | C/T | 0.777 | 0.799 | | **2.80x10^-2^** | 2.49x10^-1^ | 0.78  (0.99-0.88) | 0.538 | 0.536 | 7.86x10^-1^ | | 8.03x10^-1^ | 1.01  (0.95-1.08) |
| rs10174354 | *CACNB4* | intron | 152,614,675 | C/A | 0.81 | 0.777 | | **6.93x10^-4^** | 6.75x10^-4^ | 1.09  (1.39-1.23) | 0.759 | 0.768 | 2.29x10^-1^ | | 2.64x10^-1^ | 0.95  (0.88-1.03) |
| rs10190546 | *CACNB4* | intron | 152,642,721 | G/A | 0.821 | 0.828 | | 4.45x10^-1^ | 5.22x10^-1^ | 0.84  (1.08-0.95) | 0.742 | 0.733 | 1.94x10^-1^ | | 2.09x10^-1^ | 1.05  (0.98-1.13) |
| rs6720552 | *CACNB4* | intron | 152,662,882 | G/C | 0.834 | 0.845 | | 2.25x10^-1^ | 2.25x10^-1^ | 0.81  (1.05-0.92) | 0.800 | 0.791 | 1.66x10^-1^ | | 1.79x10^-1^ | 1.06  (0.98-1.15) |
| rs13390828 | *ARL5A* | intron | 152,664,051 | T/C | 0.863 | 0.858 | | 5.40x10^-1^ | 8.77x10^-1^ | 0.91  (1.2-1.04) | 0.980 | 0.982 | 2.60x10^-1^ | | 2.89x10^-1^ | 0.87  (0.69-1.11) |
| rs13424583 |  |  | 152,676,825 | G/C | 0.905 | 0.911 | | 3.91x10^-1^ | 5.85x10^-1^ | 0.79  (1.1-0.93) | 0.797 | 0.789 | 2.30x10^-1^ | | 2.40x10^-1^ | 1.05  (0.97-1.14) |
| rs10497094 | *STAM2* | utr-3 | 152,684,248 | G/C | 0.195 | 0.208 | | 2.02x10^-1^ | 3.68x10^-1^ | 0.92  (0.82-1.04) | 0.076 | 0.075 | 8.58x10^-1^ | | 8.40x10^-1^ | 1.01  (0.9-1.14) |
| rs3820702 | *STAM2* | intron | 152,689,099 | T/A | 0.584 | 0.601 | | 1.54x10^-1^ | 5.58x10^-1^ | 0.84  (1.03-0.93) | 0.293 | 0.299 | 4.57x10^-1^ | | 4.78x10^-1^ | 0.97  (0.91-1.05) |
| rs3768651 | *STAM2* | intron | 152,698,967 | A/C | 0.891 | 0.902 | | 1.71x10^-1^ | 2.51x10^-1^ | 0.76  (1.05-0.9) | 0.802 | 0.793 | 2.19x10^-1^ | | 2.22x10^-1^ | 1.05  (0.97-1.14) |
| rs11883823 | *STAM2* | intron | 152,702,356 | T/G | 0.645 | 0.634 | | 3.28x10^-1^ | 6.50x10^-1^ | 0.95  (1.17-1.05) | 0.920 | 0.922 | 7.28x10^-1^ | | 7.05x10^-1^ | 0.98  (0.87-1.1) |
| rs3768648 | *STAM2* | intron | 152,711,658 | A/G | 0.901 | 0.908 | | 2.81x10^-1^ | 4.38x10^-1^ | 0.78  (1.08-0.91) | 0.799 | 0.791 | 2.39x10^-1^ | | 2.47x10^-1^ | 1.05  (0.97-1.14) |
| rs10930958 | *STAM2* | intron | 152,717,655 | T/G | 0.812 | 0.837 | | **9.26x10^-3^** | 1.08x10^-1^ | 0.74  (0.96-0.84) |  |  |  | |  |  |
| rs12614451 | *STAM2* | intron | 152,720,776 | A/G | 0.786 | 0.77 | | 1.21x10^-1^ | 2.61x10^-1^ | 0.98  (1.23-1.1) | 0.924 | 0.925 | 8.44x10^-1^ | | 8.24x10^-1^ | 0.99  (0.87-1.12) |
| rs4664084 | *STAM2* | intron | 152,730,317 | T/A | 0.851 | 0.851 | | 9.47x10^-1^ | 9.96x10^-1^ | 0.87  (1.14-1) | 0.790 | 0.783 | 3.13x10^-1^ | | 3.24x10^-1^ | 1.04  (0.96-1.13) |
| rs4611639 | *STAM2* | intron | 152,733,276 | G/C | 0.9 | 0.908 | | 2.47x10^-1^ | 4.11x10^-1^ | 0.77  (1.07-0.91) | 0.797 | 0.790 | 2.56x10^-1^ | | 2.58x10^-1^ | 1.05  (0.97-1.13) |
| rs2345639 | *STAM2* | intron | 152,739,359 | A/G | 0.866 | 0.886 | | **1.32x10^-2^** | 1.90x10^-1^ | 0.72  (0.96-0.83) | 0.527 | 0.519 | 3.92x10^-1^ | | 3.96x10^-1^ | 1.03  (0.96-1.1) |
| rs10803946 |  |  | 152,741,763 | A/C | 0.172 | 0.173 | | 8.75x10^-1^ | 8.59x10^-1^ | 0.99  (0.87-1.13) | 0.210 | 0.219 | 1.77x10^-1^ | | 1.85x10^-1^ | 0.95  (0.88-1.03) |
| rs3770303 | *RPRM* | near-gene-3 | 154,041,809 | C/A | 0.912 | 0.916 | | 5.62x10^-1^ | 9.21x10^-1^ | 0.8  (1.13-0.95) | 0.838 | 0.839 | 7.69x10^-1^ | | 7.33x10^-1^ | 0.99  (0.9-1.08) |
| rs1063728 | *RPRM* | utr-3 | 154,042,745 | G/C | 0.256 | 0.243 | | 2.45x10^-1^ | 5.36x10^-1^ | 1.07  (0.96-1.19) | 0.485 | 0.470 | 7.29x10^-2^ | | 7.69x10^-2^ | 1.06  (0.99-1.13) |
| rs1524916 | *GALNT13* | intron | 154,925,452 | A/T | 0.535 | 0.527 | | 5.42x10^-1^ | 7.88x10^-1^ | 0.93  (1.14-1.03) |  |  |  | |  |  |
| rs1524915 | *GALNT13* | intron | 154,927,754 | A/C | 0.549 | 0.547 | | 8.58x10^-1^ | 8.86x10^-1^ | 0.91  (1.11-1.01) | 0.681 | 0.689 | 2.78x10^-1^ | | 7.32x10^-1^ | 0.96  (0.9-1.03) |
| rs799820 | *GALNT13* | intron | 154,928,381 | A/G | 0.609 | 0.601 | | 5.21x10^-1^ | 6.61x10^-1^ | 0.94  (1.14-1.03) | 0.672 | 0.677 | 4.82x10^-1^ | | 7.62x10^-1^ | 0.98  (0.91-1.04) |
| rs10199315 | *GALNT13* | intron | 154,933,754 | A/G | 0.521 | 0.515 | | 6.54x10^-1^ | 9.31x10^-1^ | 0.93  (1.13-1.02) | 0.678 | 0.683 | 5.37x10^-1^ | | 8.37x10^-1^ | 0.98  (0.91-1.05) |
| rs707039 | *GALNT13* | intron | 154,934,890 | T/G | 0.594 | 0.595 | | 9.08x10^-1^ | 7.11x10^-1^ | 0.9  (1.1-0.99) |  |  |  | |  |  |
| rs707047 | *GALNT13* | intron | 154,954,507 | A/G | 0.483 | 0.482 | | 8.94x10^-1^ | 7.03x10^-1^ | 1.01  (0.91-1.11) | 0.689 | 0.693 | 5.76x10^-1^ | | 9.30x10^-1^ | 0.98  (0.91-1.05) |
| rs707069 | *GALNT13* | intron | 155,005,699 | T/G | 0.961 | 0.963 | | 7.66x10^-1^ | 8.83x10^-1^ | 0.75  (1.24-0.96) | 0.877 | 0.881 | 4.29x10^-1^ | | 4.85x10^-1^ | 0.96  (0.87-1.06) |
| rs16838016 | *KCNJ3* | reference | 155,263,652 | A/G | 0.998 | 0.998 | | 5.98x10^-1^ | 4.49x10^-1^ | 0.44  (4.13-1.35) | 0.992 | 0.991 | 4.26x10^-1^ | | 4.32x10^-1^ | 1.15  (0.81-1.64) |
| rs3111033 | *KCNJ3* | reference | 155,264,124 | G/A | 0.994 | 0.996 | | 3.97x10^-1^ | 6.46x10^-1^ | 0.38  (1.47-0.75) | 0.970 | 0.974 | 1.06x10^-1^ | | 9.71x10^-2^ | 0.85  (0.7-1.04) |
| rs3111032 | *KCNJ3* | intron | 155,264,690 | A/G | 0.994 | 0.996 | | 3.97x10^-1^ | 6.46x10^-1^ | 0.38  (1.47-0.75) | 0.970 | 0.974 | 1.25x10^-1^ | | 1.14x10^-1^ | 0.86  (0.71-1.04) |
| rs11690166 | *KCNJ3* | intron | 155,264,822 | A/G | 0.305 | 0.319 | | 2.11x10^-1^ | 9.07x10^-2^ | 0.94  (0.84-1.04) | 0.533 | 0.533 | 9.85x10^-1^ | | 9.48x10^-1^ | 1  (0.94-1.07) |
| rs6435329 | *KCNJ3* | intron | 155,265,893 | T/G | 0.664 | 0.646 | | 1.28x10^-1^ | 5.86x10^-2^ | 0.98  (1.2-1.08) | 0.529 | 0.530 | 9.89x10^-1^ | | 9.91x10^-1^ | 1  (0.94-1.07) |
| rs2350121 |  |  | 155,590,390 | C/A | 0.632 | 0.623 | | 4.71x10^-1^ | 3.41x10^-1^ | 0.94  (1.15-1.04) | 0.521 | 0.520 | 8.48x10^-1^ | | 8.29x10^-1^ | 1.01  (0.94-1.07) |
| rs7578557 |  |  | 156,626,081 | G/A | 0.644 | 0.632 | | 3.22x10^-1^ | 2.23x10^-1^ | 0.95  (1.17-1.05) | 0.539 | 0.539 | 9.98x10^-1^ | | 9.72x10^-1^ | 1  (0.94-1.07) |
| rs12471739 |  |  | 156,720,471 | C/A | 0.373 | 0.372 | | 9.27x10^-1^ | 7.35x10^-1^ | 1.01  (0.91-1.11) |  |  |  | |  |  |
| rs2592371 |  |  | 157,772,721 | A/G | 0.374 | 0.354 | | 1.02x10^-1^ | 6.25x10^-1^ | 1.09  (0.98-1.21) | 0.834 | 0.826 | 1.76x10^-1^ | | 1.75x10^-1^ | 1.06  (0.97-1.16) |
| rs6714869 |  |  | 158,086,131 | A/T | 0.461 | 0.449 | | 3.48x10^-1^ | 7.70x10^-1^ | 1.05  (0.95-1.15) | 0.752 | 0.738 | 5.26x10^-2^ | | 5.47x10^-2^ | 1.08  (1-1.16) |
| rs7568889 | *ACVR1C* | intron | 158,101,266 | T/G | 0.961 | 0.962 | | 8.00x10^-1^ | 8.63x10^-1^ | 0.75  (1.24-0.97) | 0.885 | 0.877 | 1.27x10^-1^ | | 1.14x10^-1^ | 1.08  (0.98-1.2) |
| rs6437105 | *ACVR1C* | intron | 158,103,059 | T/A | 0.903 | 0.903 | | 9.84x10^-1^ | 9.93x10^-1^ | 0.85  (1.17-1) | 0.889 | 0.882 | 1.65x10^-1^ | | 1.62x10^-1^ | 1.07  (0.97-1.19) |
| rs16826016 | *ACVR1C* | intron | 158,113,621 | A/C | 0.527 | 0.518 | | 4.45x10^-1^ | 8.82x10^-1^ | 0.94  (1.15-1.04) | 0.837 | 0.826 | 1.12x10^-1^ | | 1.25x10^-1^ | 1.08  (0.98-1.18) |
| rs4299296 | *ACVR1C* | intron | 158,117,377 | A/G | 0.596 | 0.611 | | 2.20x10^-1^ | 7.33x10^-1^ | 0.85  (1.04-0.94) | 0.228 | 0.233 | 4.70x10^-1^ | | 4.96x10^-1^ | 0.97  (0.9-1.05) |
| rs6437106 | *ACVR1C* | intron | 158,118,442 | T/C | 0.511 | 0.548 | | **3.29x10^-3^** | 2.17x10^-2^ | 0.78  (0.95-0.86) |  |  |  | |  |  |
| rs6748047 | *ACVR1C* | intron | 158,140,692 | A/C | 0.72 | 0.715 | | 6.24x10^-1^ | 5.09x10^-1^ | 0.92  (1.14-1.03) | 0.600 | 0.599 | 9.35x10^-1^ | | 9.53x10^-1^ | 1  (0.94-1.07) |
| rs4377290 | *ACVR1C* | intron | 158,145,929 | C/T | 0.54 | 0.554 | | 2.77x10^-1^ | 3.84x10^-1^ | 0.86  (1.04-0.95) | 0.511 | 0.510 | 8.27x10^-1^ | | 8.48x10^-1^ | 1.01  (0.94-1.07) |
| rs4664978 | *PKP4* | intron | 159,148,083 | A/G | 0.462 | 0.47 | | 4.98x10^-1^ | 9.28x10^-1^ | 0.97  (0.88-1.07) | 0.204 | 0.199 | 4.36x10^-1^ | | 4.97x10^-1^ | 1.03  (0.95-1.12) |
| rs2078507 | *CCDC148* | intron | 159,272,176 | T/G | 0.964 | 0.962 | | 7.02x10^-1^ | 3.50x10^-1^ | 0.81  (1.36-1.05) | 0.866 | 0.864 | 7.17x10^-1^ | | 7.51x10^-1^ | 1.02  (0.93-1.12) |
| rs6730860 |  |  | 159,331,770 | C/A | 0.765 | 0.771 | | 5.42x10^-1^ | 8.94x10^-1^ | 0.86  (1.08-0.97) | 0.655 | 0.644 | 1.44x10^-1^ | | 1.39x10^-1^ | 1.05  (0.98-1.13) |
| rs11684616 | *DAPL1* | intron | 159,369,564 | A/G | 0.996 | 0.997 | | 4.06x10^-1^ | 6.42x10^-1^ | 0.3  (1.63-0.7) | 0.975 | 0.972 | 1.75x10^-1^ | | 1.84x10^-1^ | 1.15  (0.94-1.4) |
| rs17810428 | *DAPL1* | intron | 159,369,697 | G/A | 0.946 | 0.948 | | 6.74x10^-1^ | 6.14x10^-1^ | 0.77  (1.19-0.95) |  |  |  | |  |  |
| rs11679019 | *DAPL1* | intron | 159,369,757 | C/A | 0.949 | 0.953 | | 4.32x10^-1^ | 8.19x10^-1^ | 0.73  (1.14-0.91) |  |  |  | |  |  |
| rs1878099 | *DAPL1* | intron | 159,371,151 | A/G | 0.711 | 0.706 | | 6.55x10^-1^ | 4.51x10^-1^ | 0.92  (1.14-1.02) | 0.567 | 0.561 | 5.07x10^-1^ | | 5.35x10^-1^ | 1.02  (0.96-1.09) |
| rs9869 | *DAPL1* | missense | 159,371,845 | A/G | 0.451 | 0.467 | | 2.07x10^-1^ | 1.46x10^-1^ | 0.94  (0.85-1.04) | 0.582 | 0.573 | 2.25x10^-1^ | | 2.21x10^-1^ | 1.04  (0.98-1.11) |
| rs10497199 | *DAPL1* | missense | 159,371,862 | G/A | 0.943 | 0.95 | | 2.25x10^-1^ | 8.74x10^-1^ | 0.71  (1.08-0.88) | 0.696 | 0.689 | 3.98x10^-1^ | | 4.15x10^-1^ | 1.03  (0.96-1.1) |
| rs1011770 | *BAZ2B* | intron | 159,946,964 | T/G | 0.995 | 0.994 | | 5.96x10^-1^ | 3.79x10^-1^ | 0.61  (2.39-1.2) | 0.980 | 0.982 | 3.72x10^-1^ | | 3.99x10^-1^ | 0.9  (0.71-1.14) |
| rs10195878 | *BAZ2B* | intron | 159,999,839 | C/T | 0.719 | 0.732 | | 2.50x10^-1^ | 6.35x10^-1^ | 0.84  (1.05-0.94) | 0.559 | 0.561 | 7.88x10^-1^ | | 7.79x10^-1^ | 0.99  (0.93-1.06) |
| rs7568166 |  |  | 161,598,238 | A/G | 0.933 | 0.949 | | **6.20x10^-3^** | 3.28x10^-2^ | 0.61  (0.92-0.75) | 0.817 | 0.817 | 9.36x10^-1^ | | 9.19x10^-1^ | 1  (0.92-1.08) |
| rs17713024 |  |  | 162,812,865 | C/A | 0.999 | 0.996 | | 6.58x10^-2^ | 6.13x10^-2^ | 0.89  (8.42-2.74) | 0.987 | 0.985 | 1.91x10^-1^ | | 1.82x10^-1^ | 1.2  (0.91-1.57) |
| **rs1990760** | ***IFIH1*** | **missense** | **162,832,297** | **G/A** | **0.799** | **0.826** | | **5.27x10^-3^** | 7.51x10^-2^ | **0.74**  **(0.95-0.84)** | **0.375** | **0.406** | **8.82x10^-5^** | | 1.09x10^-4^ | **0.88**  **(0.82-0.94)** |
| rs17764770 | *IFIH1* | Intron | 162,832,556 | T/G | 0.992 | 0.996 | | 9.53x10^-2^ | 1.84x10^-1^ | 0.31  (1.11-0.58) | 0.964 | 0.964 | 7.87x10^-1^ | | 7.81x10^-1^ | 1.02  (0.86-1.22) |
| rs35667974 | *IFIH1* | reference | 162,832,883 | A/G | 0.998 | 0.996 | | 1.57x10^-1^ | 9.09x10^-2^ | 0.76  (5.14-1.97) | 0.991 | 0.986 | **9.08x10^-3^** | | 8.14x10^-3^ | 1.5  (1.1-2.03) |
| rs12474565 | *IFIH1* | intron | 162,833,147 | G/A | 0.625 | 0.575 | | **3.52x10^-5^** | 7.35x10^-4^ | 1.12  (1.36-1.23) | 0.984 | 0.984 | 7.66x10^-1^ | | 7.58x10^-1^ | 0.96  (0.74-1.24) |
| rs6760785 | *IFIH1* | intron | 162,834,030 | G/A | 0.935 | 0.93 | | 4.36x10^-1^ | 6.46x10^-1^ | 0.89  (1.31-1.08) | 0.999 | 1.000 | 2.24x10^-1^ | | 2.56x10^-1^ | 0.28  (0.03-2.51) |
| rs3747517 | *IFIH1* | reference | 162,837,070 | G/A | 0.594 | 0.614 | | 9.67x10^-2^ | 5.52x10^-2^ | 0.83  (1.02-0.92) | 0.740 | 0.727 | 7.05x10^-2^ | | 7.10x10^-2^ | 1.07  (0.99-1.15) |
| rs13418718 | *IFIH1* | reference | 162,837,129 | G/A | 0.711 | 0.666 | | **8.92x10^-5^** | 1.42x10^-3^ | 1.11  (1.37-1.23) | 0.997 | 0.998 | 7.43x10^-1^ | | 7.51x10^-1^ | 0.9  (0.46-1.73) |
| rs13418892 | *IFIH1* | intron | 162,837,454 | C/A | 0.849 | 0.827 | | **1.65x10^-2^** | 5.75x10^-2^ | 1.03  (1.34-1.17) | 0.999 | 0.999 | 7.84x10^-1^ | | 7.76x10^-1^ | 0.87  (0.32-2.34) |
| rs35744605 | *IFIH1* | reference | 162,842,336 | G/A | 1 | 0.999 | | 1.12x10^-1^ | 9.99x10^-1^ |  | 0.996 | 0.995 | 1.82x10^-1^ | | 1.89x10^-1^ | 1.4  (0.85-2.31) |
| rs2287292 | *IFIH1* | intron | 162,844,508 | C/A | 0.647 | 0.607 | | **8.34x10^-4^** | 8.98x10^-3^ | 1.07  (1.31-1.19) | 0.984 | 0.985 | 5.74x10^-1^ | | 5.68x10^-1^ | 0.93  (0.72-1.2) |
| rs7603224 | *IFIH1* | intron | 162,845,295 | A/G | 0.884 | 0.865 | | **2.14x10^-2^** | 6.63x10^-2^ | 1.03  (1.37-1.19) | 0.999 | 0.999 | 5.77x10^-1^ | | 6.07x10^-1^ | 0.75  (0.27-2.1) |
| **rs10930046** | ***IFIH1*** | **reference** | **162,846,229** | **G/A** | **0.374** | **0.417** | | **3.75x10^-4^** | 4.27x10^-3^ | **0.83**  **(0.76-0.92)** | **0.016** | **0.016** | **8.38x10^-1^** | | 8.31x10^-1^ | **1.03**  **(0.79-1.33)** |
| rs7602311 | *IFIH1* | intron | 162,848,411 | T/G | 0.715 | 0.674 | | **2.93x10^-4^** | 3.08x10^-3^ | 1.09  (1.35-1.21) | 0.984 | 0.985 | 7.51x10^-1^ | | 7.41x10^-1^ | 0.96  (0.74-1.24) |
| rs16846565 | *IFIH1* | intron | 162,849,040 | A/G | 0.992 | 0.995 | | 1.82x10^-1^ | 3.38x10^-1^ | 0.36  (1.22-0.66) | 0.987 | 0.987 | 7.62x10^-1^ | | 7.50x10^-1^ | 0.96  (0.72-1.27) |
| rs4664460 | *IFIH1* | intron | 162,851,670 | G/A | 0.708 | 0.667 | | **4.16x10^-4^** | 4.09x10^-3^ | 1.09  (1.34-1.21) | 0.984 | 0.985 | 7.88x10^-1^ | | 7.78x10^-1^ | 0.97  (0.74-1.25) |
| rs12476567 | *IFIH1* | intron | 162,856,657 | A/C | 0.708 | 0.668 | | **5.52x10^-4^** | 5.01x10^-3^ | 1.08  (1.33-1.2) | 0.984 | 0.985 | 7.26x10^-1^ | | 7.17x10^-1^ | 0.95  (0.74-1.24) |
| rs16846585 | *IFIH1* | intron | 162,861,782 | G/C | 0.71 | 0.67 | | **4.01x10^-4^** | 3.86x10^-3^ | 1.09  (1.35-1.21) | 0.985 | 0.984 | 8.58x10^-1^ | | 8.81x10^-1^ | 1.02  (0.78-1.34) |
| **rs13023380** | ***IFIH1*** | **intron** | **162,862,609** | **G/A** | **0.885** | **0.895** | | **1.90x10^-1^** | 9.35x10^-1^ | **0.77**  **(1.05-0.9)** | **0.433** | **0.461** | **9.53x10^-4^** | | 7.37x10^-4^ | **0.89**  **(0.83-0.95)** |
| rs7563980 | *IFIH1* | intron | 162,864,892 | A/T | 0.232 | 0.261 | | **8.58x10^-3^** | 4.13x10^-2^ | 0.86  (0.77-0.96) | 0.002 | 0.002 | 8.71x10^-1^ | | 8.76x10^-1^ | 0.95  (0.49-1.84) |
| rs16846600 | *IFIH1* | intron | 162,867,580 | A/G | 0.683 | 0.656 | | **1.92x10^-2^** | 1.01x10^-1^ | 1.02  (1.25-1.13) | 0.984 | 0.985 | 8.60x10^-1^ | | 8.52x10^-1^ | 0.98  (0.75-1.27) |
| rs17715343 | *IFIH1* | intron | 162,875,992 | C/G | 0.986 | 0.987 | | 7.32x10^-1^ | 8.30x10^-1^ | 0.61  (1.42-0.93) | 0.913 | 0.897 | **1.37x10^-3^** | | 1.35x10^-3^ | 1.2  (1.07-1.34) |
| rs2060447 | *KCNH7* | intron | 163,308,799 | T/C | 0.822 | 0.847 | | **6.49x10^-3^** | 5.00x10^-1^ | 0.73  (0.95-0.83) | 0.786 | 0.777 | 1.73x10^-1^ | | 1.71x10^-1^ | 1.06  (0.98-1.14) |
| rs16847319 | *KCNH7* | intron | 163,346,495 | G/A | 0.532 | 0.522 | | 4.45x10^-1^ | 9.88x10^-1^ | 0.94  (1.14-1.04) | 0.877 | 0.883 | 2.62x10^-1^ | | 2.61x10^-1^ | 0.95  (0.86-1.04) |
