# Supplementary material for: Admixture Mapping in Lupus Identifies Multiple Functional Variants within IFIH1 Associated with Apoptosis, Inflammation, and Autoantibody Production
Source: PLoS Genet. 2013 Feb 18;9(2):e1003222. doi: 10.1371/journal.pgen.1003222 (PMC3575474; doi:10.1371/journal.pgen.1003222)
Supplement: Table S5 — Genetic models of association of IFIH1 with SLE. Genetic models at each independent SNP for African-Americans (AA) and European-Americans (EA) show that the best model for rs1990760 and rs130233890 are allelic, and dominant for rs10930046. The best model was chosen using the Akaike information criterion (AIC). (DOCX) [file pgen.1003222.s011.docx]

**Table S5.** **Genetic models of association of *IFIH1* with SLE.** Genetic models at each independent SNP for African-Americans (AA) and European-Americans (EA) show that the best model for rs1990760 and rs130233890 are allelic, and dominant for rs10930046. The best model was chosen using the Akaike information criterion (AIC).

| **SNP** | **A1** | **A2** | **TEST** | **AA** | | | | **EA** | | | |
| --- | --- | --- | --- | --- | --- | --- | --- | --- | --- | --- | --- |
|  |  |  |  | **Affected** | **Control** | **P-value** | **AIC** | **Affected** | **Control** | **P-value** | **AIC** |
| **rs1990760** | A | G | ALLELIC | 608/2416 | 1522/7374 | 2.02x10^-4^ | 6741.3 | 4805/2877 | 11569/7707 | 1.22x10^-4^ | 16098.8 |
|  |  |  | GENO | 66/476/970 | 117/1288/3043 | 2.58x10^-4^ | 6741.2 | 1520/1765/556 | 3479/4611/1548 | 4.42x10^-4^ | 16100.1 |
|  |  |  | TREND | 608/2416 | 1522/7374 | **1.90x10^-4^** | 6368.8 | 4805/2877 | 11569/7707 | **1.30x10^-4^** | 16099 |
|  |  |  | DOM | 542/970 | 1405/3043 | 2.28x10^-3^ | 6745.7 | 1520/2321 | 3479/6159 | 1.62x10^-4^ | 16099.4 |
|  |  |  | REC | 66/1446 | 117/4331 | 7.31x10^-4^ | 6744.4 | 3285/556 | 8090/1548 | 2.20x10^-2^ | 16108.2 |
| **rs10930046** | G | A | ALLELIC | 1130/1894 | 3760/5028 | 1.81x10^-7^ | 6695.6 | 125/7683 | 258/19142 | 8.61x10^-2^ | 16310.2 |
|  |  |  | GENO | 227/676/609 | 781/2198/1415 | 6.98x10^-8^ | 6692.7 | 2/121/3781 | 3/252/9445 | 2.30x10^-1^ | 16312.2 |
|  |  |  | TREND | 1130/1894 | 3760/5028 | 1.74x10^-7^ | 6337.7 | 125/7683 | 258/19142 | 8.80x10^-2^ | 16310.3 |
|  |  |  | DOM | 903/609 | 2979/1415 | **1.16x10^-8^** | 6691.1 | 123/3781 | 255/9445 | 9.37x10^-2^ | 16310.3 |
|  |  |  | REC | 227/1285 | 781/3613 | 1.38x10^-2^ | 6717 | 2/1/3902 | 3/1/9697 | 6.85x10^-1^ | 16312.7 |
| **rs13023380** | A | G | ALLELIC | 333/2563 | 770/7866 | 4.33x10^-5^ | 6486.9 | 4100/3134 | 9886/9048 | 9.52x10^-11^ | 15389.5 |
|  |  |  | GENO | 22/289/1137 | 28/714/3576 | 6.66x10^-5^ | 6487.1 | 1128/1844/645 | 2597/4692/2178 | 8.08x10^-11^ | 15386.2 |
|  |  |  | TREND | 333/2563 | 770/7866 | **4.12x10^-5^** | 6187.6 | 4100/3134 | 9886/9048 | **8.76x10^-11^** | 15393.1 |
|  |  |  | DOM | 311/1137 | 742/3576 | 2.52x10^-4^ | 6490.1 | 1128/2489 | 2597/6870 | 2.09x10^-5^ | 15413.8 |
|  |  |  | REC | 22/1426 | 28/4290 | 1.98x10^-3^ | 6494.6 | 2972/645 | 7289/2178 | 1.24x10^-10^ | 15389.1 |
