# Supplementary material for: Admixture Mapping in Lupus Identifies Multiple Functional Variants within IFIH1 Associated with Apoptosis, Inflammation, and Autoantibody Production
Source: PLoS Genet. 2013 Feb 18;9(2):e1003222. doi: 10.1371/journal.pgen.1003222 (PMC3575474; doi:10.1371/journal.pgen.1003222)
Supplement: Table S6 — Imputation based association analysis for African Americans (N = 1525 cases; 4485 controls). Pc denotes the local ancestry-corrected P-value. ∧Rsq denotes the quality measure of the squared correlation between imputed and true genotypes. * denotes the 11 SNPs that were genotyped in DHS controls. (DOCX) [file pgen.1003222.s012.docx]

**Table S6. Imputation based association analysis for African Americans** (N=1525 cases; 4485 controls). Pc denotes the local ancestry-corrected P-value. ^Rsq denotes the quality measure of the squared correlation between imputed and true genotypes. * denotes the 11 SNPs that were genotyped in DHS controls.

| **SNP** | **Position** | **Status** | **A1** | **A2** | **Affected** | **Control** | **OR** | **P-value** | **Pc** | **Rsq^** |
| --- | --- | --- | --- | --- | --- | --- | --- | --- | --- | --- |
| rs2160710 | 162817515 | imputed | G | A | 0.08 | 0.09 | 0.88 | 8.23x10^-2^ | 1.22x10^-1^ | 0.81 |
| rs16846479 | 162817921 | imputed | G | A | 0.07 | 0.08 | 0.90 | 2.12x10^-1^ | 3.34x10^-1^ | 0.93 |
| rs4664456 | 162818324 | imputed | A | T | 0.08 | 0.09 | 0.90 | 1.55x10^-1^ | 2.18x10^-1^ | 0.78 |
| rs4664458 | 162818361 | imputed | A | C | 0.08 | 0.09 | 0.90 | 1.60x10^-1^ | 2.25x10^-1^ | 0.80 |
| rs2111485 | 162818782 | imputed | G | A | 0.21 | 0.19 | 1.15 | 8.81x10^-3^ | 7.98x10^-2^ | 0.82 |
| rs12464666 | 162819001 | imputed | T | A | 0.08 | 0.09 | 0.89 | 1.36x10^-1^ | 1.91x10^-1^ | 0.64 |
| rs12476601 | 162819166 | imputed | A | G | 0.08 | 0.09 | 0.90 | 1.60x10^-1^ | 2.25x10^-1^ | 0.81 |
| seq-NOVEL-10317 | 162819323 | imputed | A | C | 0.08 | 0.09 | 0.90 | 1.60x10^-1^ | 2.25x10^-1^ | 0.81 |
| rs4664459 | 162819959 | imputed | A | T | 0.08 | 0.09 | 0.89 | 1.39x10^-1^ | 1.98x10^-1^ | 0.79 |
| rs10207052 | 162820686 | imputed | A | G | 0.03 | 0.03 | 0.86 | 2.12x10^-1^ | 2.67x10^-1^ | 0.52 |
| rs12477811 | 162820716 | imputed | A | C | 0.08 | 0.09 | 0.90 | 1.44x10^-1^ | 2.04x10^-1^ | 0.81 |
| rs16846492 | 162820758 | imputed | A | G | 0.08 | 0.09 | 0.89 | 1.34x10^-1^ | 1.91x10^-1^ | 0.77 |
| rs10210133 | 162821522 | imputed | A | G | 0.29 | 0.32 | 0.84 | 1.67x10^-4^ | 1.32x10^-3^ | 0.76 |
| rs10165813 | 162822162 | imputed | A | G | 0.24 | 0.24 | 0.98 | 6.92x10^-1^ | 9.12x10^-1^ | 0.78 |
| rs10189577 | 162822244 | imputed | T | A | 0.15 | 0.15 | 1.03 | 5.86x10^-1^ | 3.15x10^-1^ | 0.68 |
| rs16846499 | 162822781 | imputed | T | A | 0.37 | 0.42 | 0.81 | 6.95x10^-7^ | 1.10x10^-5^ | 0.90 |
| rs6752932 | 162823306 | imputed | G | A | 0.08 | 0.09 | 0.89 | 1.38x10^-1^ | 2.34x10^-1^ | 0.95 |
| rs6738036 | 162823485 | imputed | C | G | 0.01 | 0.01 | 0.87 | 4.75x10^-1^ | 5.29x10^-1^ | 0.97 |
| rs16846501 | 162824062 | imputed | A | C | 0.27 | 0.31 | 0.83 | 3.60x10^-5^ | 3.05x10^-4^ | 0.97 |
| rs13420023 | 162825379 | imputed | G | A | 0.27 | 0.31 | 0.84 | 1.33x10^-4^ | 1.04x10^-3^ | 0.91 |
| rs13394780 | 162825457 | imputed | G | C | 0.27 | 0.31 | 0.84 | 1.16x10^-4^ | 9.11x10^-4^ | 0.93 |
| rs13394899 | 162825579 | imputed | A | C | 0.27 | 0.31 | 0.84 | 1.21x10^-4^ | 9.54x10^-4^ | 0.93 |
| rs6721353 | 162826356 | imputed | G | A | 0.01 | 0.02 | 0.75 | 9.25x10^-2^ | 1.06x10^-1^ | 0.63 |
| rs10186055 | 162828161 | imputed | A | G | 0.38 | 0.43 | 0.80 | 2.40x10^-7^ | 3.92x10^-6^ | 1.00 |
| rs16846511 | 162828277 | imputed | A | G | 0.07 | 0.08 | 0.89 | 1.54x10^-1^ | 2.53x10^-1^ | 0.95 |
| rs16846512 | 162828440 | imputed | G | A | 0.36 | 0.41 | 0.81 | 1.19x10^-6^ | 2.02x10^-5^ | 0.98 |
| rs6729724 | 162828557 | imputed | G | A | 0.29 | 0.33 | 0.82 | 1.65x10^-5^ | 1.59x10^-4^ | 1.00 |
| rs6755566 | 162829063 | imputed | C | A | 0.29 | 0.33 | 0.82 | 1.65x10^-5^ | 1.59x10^-4^ | 1.00 |
| rs6755575 | 162829093 | imputed | G | A | 0.36 | 0.41 | 0.81 | 1.07x10^-6^ | 1.82x10^-5^ | 0.99 |
| rs10187350* | 162830127 | imputed | C | A | 0.36 | 0.41 | 0.81 | 1.07x10^-6^ | 1.82x10^-5^ | 0.99 |
| rs741439 | 162830669 | imputed | C | G | 0.29 | 0.33 | 0.82 | 1.57x10^-5^ | 1.52x10^-4^ | 0.99 |
| rs7558405 | 162831332 | imputed | G | A | 0.38 | 0.43 | 0.80 | 2.27x10^-7^ | 3.74x10^-6^ | 1.00 |
| rs11891191* | 162831950 | imputed | A | G | 0.29 | 0.33 | 0.82 | 1.57x10^-5^ | 1.52x10^-4^ | 1.00 |
| rs1990760* | 162832297 | typed | G | A | 0.80 | 0.83 | 0.81 | 1.04x10^-4^ | 1.86x10^-3^ | 1.00 |
| rs12474565* | 162833147 | typed | G | A | 0.38 | 0.43 | 0.80 | 2.27x10^-7^ | 3.77x10^-6^ | 1.00 |
| rs7578928 | 162833852 | imputed | G | A | 0.36 | 0.41 | 0.80 | 3.53x10^-7^ | 6.58x10^-6^ | 0.98 |
| rs6760785 | 162834030 | typed | A | G | 0.07 | 0.08 | 0.89 | 1.66x10^-1^ | 2.71x10^-1^ | 0.99 |
| rs13415800 | 162836784 | imputed | A | C | 0.29 | 0.33 | 0.82 | 1.49x10^-5^ | 1.46x10^-4^ | 1.00 |
| rs3747518 | 162836971 | imputed | G | A | 0.38 | 0.43 | 0.80 | 2.27x10^-7^ | 3.77x10^-6^ | 1.00 |
| rs3747517* | 162837070 | typed | A | G | 0.41 | 0.39 | 1.09 | 4.17x10^-2^ | 2.52x10^-2^ | 1.00 |
| rs13418718* | 162837129 | typed | A | G | 0.29 | 0.33 | 0.82 | 1.49x10^-5^ | 1.46x10^-4^ | 1.00 |
| rs41399348 | 162837150 | imputed | T | A | 0.07 | 0.08 | 0.89 | 1.66x10^-1^ | 2.71x10^-1^ | 0.99 |
| rs10200223 | 162839661 | imputed | G | A | 0.29 | 0.33 | 0.82 | 1.49x10^-5^ | 1.46x10^-4^ | 1.00 |
| rs10188109 | 162839750 | imputed | A | C | 0.37 | 0.41 | 0.81 | 1.74x10^-6^ | 2.42x10^-5^ | 1.00 |
| rs9287816 | 162840418 | imputed | G | A | 0.29 | 0.33 | 0.82 | 1.49x10^-5^ | 1.46x10^-4^ | 1.00 |
| rs10490426 | 162840933 | imputed | C | A | 0.07 | 0.08 | 0.89 | 1.66x10^-1^ | 2.71x10^-1^ | 0.99 |
| rs13388189 | 162840999 | imputed | G | A | 0.38 | 0.43 | 0.80 | 2.27x10^-7^ | 3.77x10^-6^ | 1.00 |
| rs888284 | 162842734 | imputed | T | A | 0.29 | 0.33 | 0.82 | 1.49x10^-5^ | 1.46x10^-4^ | 1.00 |
| rs2287292* | 162844508 | typed | C | A | 0.35 | 0.40 | 0.82 | 4.67x10^-6^ | 5.66x10^-5^ | 0.99 |
| rs2287293 | 162844973 | imputed | C | G | 0.29 | 0.33 | 0.83 | 4.74x10^-5^ | 3.28x10^-4^ | 0.98 |
| rs7590692 | 162845017 | imputed | G | A | 0.28 | 0.32 | 0.83 | 3.51x10^-5^ | 2.47x10^-4^ | 0.94 |
| rs7603101 | 162845181 | imputed | G | A | 0.38 | 0.43 | 0.80 | 3.01x10^-7^ | 4.82x10^-6^ | 0.99 |
| rs6432713 | 162845335 | imputed | A | G | 0.07 | 0.08 | 0.89 | 1.66x10^-1^ | 2.71x10^-1^ | 0.98 |
| rs7603431 | 162845427 | imputed | A | T | 0.28 | 0.32 | 0.82 | 2.48x10^-5^ | 2.22x10^-4^ | 0.98 |
| rs12479043 | 162846117 | imputed | G | C | 0.31 | 0.33 | 0.92 | 5.23x10^-2^ | 1.56x10^-1^ | 0.66 |
| rs10930046* | 162846229 | typed | G | A | 0.38 | 0.43 | 0.80 | 3.01x10^-7^ | 4.87x10^-6^ | 1.00 |
| rs6759894 | 162846729 | imputed | C | A | 0.12 | 0.12 | 1.04 | 5.00x10^-1^ | 2.77x10^-1^ | 0.80 |
| rs6734769 | 162847031 | imputed | A | G | 0.29 | 0.32 | 0.83 | 6.60x10^-5^ | 4.49x10^-4^ | 1.00 |
| rs12464391 | 162847812 | imputed | A | C | 0.29 | 0.32 | 0.83 | 6.60x10^-5^ | 4.49x10^-4^ | 1.00 |
| rs7602311* | 162848411 | typed | G | A | 0.29 | 0.32 | 0.83 | 6.60x10^-5^ | 4.49x10^-4^ | 1.00 |
| rs7588675 | 162848589 | imputed | A | G | 0.28 | 0.32 | 0.82 | 3.02x10^-5^ | 2.66x10^-4^ | 0.99 |
| rs11900837 | 162848952 | imputed | A | G | 0.01 | 0.01 | 0.85 | 3.85x10^-1^ | 4.34x10^-1^ | 0.34 |
| rs7567566 | 162849223 | imputed | G | A | 0.38 | 0.43 | 0.81 | 5.26x10^-7^ | 8.52x10^-6^ | 0.98 |
| rs7580040 | 162849260 | imputed | C | A | 0.07 | 0.08 | 0.89 | 1.66x10^-1^ | 2.71x10^-1^ | 0.98 |
| rs2389683 | 162849559 | imputed | A | C | 0.38 | 0.43 | 0.81 | 8.15x10^-7^ | 1.28x10^-5^ | 0.96 |
| rs10179671 | 162849574 | imputed | G | A | 0.36 | 0.40 | 0.83 | 1.80x10^-5^ | 1.95x10^-4^ | 0.98 |
| rs4664460 | 162851670 | typed | G | A | 0.29 | 0.33 | 0.85 | 3.02x10^-4^ | 1.80x10^-3^ | 0.99 |
| rs7565446 | 162852642 | imputed | A | C | 0.38 | 0.43 | 0.81 | 1.32x10^-6^ | 1.98x10^-5^ | 0.94 |
| rs7591660 | 162852693 | imputed | G | A | 0.36 | 0.40 | 0.83 | 2.90x10^-5^ | 2.99x10^-4^ | 0.98 |
| rs6718365 | 162853578 | imputed | A | G | 0.29 | 0.33 | 0.84 | 1.62x10^-4^ | 1.05x10^-3^ | 0.97 |
| rs6718470 | 162853608 | imputed | A | C | 0.29 | 0.33 | 0.85 | 2.77x10^-4^ | 1.67x10^-3^ | 0.99 |
| imm_2_162853712 | 162853712 | imputed | G | C | 0.29 | 0.33 | 0.85 | 3.02x10^-4^ | 1.80x10^-3^ | 0.99 |
| rs6733639 | 162853816 | imputed | C | A | 0.28 | 0.32 | 0.84 | 1.80x10^-4^ | 1.34x10^-3^ | 0.98 |
| rs12466054 | 162854077 | imputed | A | G | 0.36 | 0.40 | 0.84 | 3.66x10^-5^ | 3.66x10^-4^ | 0.95 |
| rs16846573 | 162854160 | imputed | G | A | 0.07 | 0.08 | 0.90 | 1.78x10^-1^ | 2.88x10^-1^ | 0.92 |
| rs4664461 | 162854648 | imputed | G | A | 0.29 | 0.33 | 0.85 | 3.02x10^-4^ | 1.80x10^-3^ | 0.99 |
| rs13313773 | 162854938 | imputed | A | T | 0.36 | 0.40 | 0.83 | 2.90x10^-5^ | 2.95x10^-4^ | 0.93 |
| rs6730036 | 162856318 | imputed | A | G | 0.28 | 0.32 | 0.84 | 1.88x10^-4^ | 1.39x10^-3^ | 0.98 |
| rs12476567 | 162856657 | typed | C | A | 0.29 | 0.33 | 0.85 | 3.15x10^-4^ | 1.87x10^-3^ | 0.98 |
| rs11897331 | 162856988 | imputed | G | A | 0.01 | 0.01 | 0.80 | 2.57x10^-1^ | 2.95x10^-1^ | 0.81 |
| rs4664462 | 162857710 | imputed | C | A | 0.29 | 0.33 | 0.85 | 3.15x10^-4^ | 1.87x10^-3^ | 0.98 |
| rs4664463 | 162858211 | imputed | G | A | 0.38 | 0.43 | 0.81 | 1.01x10^-6^ | 1.57x10^-5^ | 0.98 |
| imm_2_162859217 | 162859217 | imputed | A | G | 0.01 | 0.02 | 0.67 | 3.50x10^-2^ | 3.72x10^-2^ | 0.26 |
| rs16846582 | 162859255 | imputed | G | A | 0.07 | 0.08 | 0.89 | 1.72x10^-1^ | 2.79x10^-1^ | 0.98 |
| rs974551 | 162859615 | imputed | G | A | 0.28 | 0.31 | 0.87 | 3.10x10^-3^ | 1.38x10^-2^ | 0.90 |
| rs10439291 | 162860268 | imputed | A | G | 0.29 | 0.33 | 0.85 | 3.15x10^-4^ | 1.87x10^-3^ | 0.98 |
| rs10439256 | 162860597 | imputed | G | A | 0.29 | 0.33 | 0.85 | 2.89x10^-4^ | 1.73x10^-3^ | 0.98 |
| rs7578932 | 162861294 | imputed | A | G | 0.12 | 0.12 | 1.07 | 3.22x10^-1^ | 1.58x10^-1^ | 0.85 |
| rs13023380* | 162862609 | typed | G | A | 0.89 | 0.91 | 0.76 | 1.03x10^-4^ | 2.96x10^-3^ | 0.95 |
| rs2163215 | 162863326 | imputed | A | C | 0.30 | 0.33 | 0.85 | 4.47x10^-4^ | 2.55x10^-3^ | 0.97 |
| rs73971815 | 162864104 | imputed | G | A | 0.38 | 0.42 | 0.85 | 1.21x10^-4^ | 1.13x10^-3^ | 0.86 |
| rs7590274 | 162864578 | imputed | A | G | 0.12 | 0.12 | 1.04 | 5.86x10^-1^ | 3.40x10^-1^ | 0.75 |
| rs7563980 | 162864892 | typed | A | T | 0.23 | 0.27 | 0.82 | 6.84x10^-5^ | 4.20x10^-4^ | 0.91 |
| rs10195025 | 162866405 | imputed | A | G | 0.33 | 0.37 | 0.83 | 3.40x10^-5^ | 2.99x10^-4^ | 0.90 |
| rs7559103 | 162866902 | imputed | G | A | 0.12 | 0.12 | 1.03 | 6.39x10^-1^ | 3.80x10^-1^ | 0.82 |
| rs16846600 | 162867580 | typed | G | A | 0.32 | 0.36 | 0.82 | 1.58x10^-5^ | 1.47x10^-4^ | 0.93 |
| rs12478709 | 162872037 | imputed | G | A | 0.33 | 0.37 | 0.82 | 1.22x10^-5^ | 1.40x10^-4^ | 0.90 |
| rs10197553* | 162872459 | imputed | A | C | 0.32 | 0.36 | 0.82 | 5.77x10^-6^ | 6.83x10^-5^ | 0.89 |
| rs10203640 | 162873961 | imputed | A | G | 0.33 | 0.37 | 0.81 | 1.78x10^-6^ | 2.24x10^-5^ | 0.90 |
| rs11885573 | 162874217 | imputed | G | A | 0.01 | 0.02 | 0.87 | 4.44x10^-1^ | 4.99x10^-1^ | 0.82 |
| rs11904197 | 162875877 | imputed | A | G | 0.01 | 0.02 | 0.87 | 4.44x10^-1^ | 4.99x10^-1^ | 0.82 |
| rs1864430 | 162880550 | imputed | G | T | 0.33 | 0.37 | 0.81 | 1.78x10^-6^ | 2.24x10^-5^ | 0.90 |
| rs13394680 | 162885544 | imputed | A | C | 0.18 | 0.17 | 1.06 | 2.96x10^-1^ | 1.30x10^-1^ | 0.81 |
| rs12472542 | 162886024 | imputed | G | A | 0.19 | 0.18 | 1.09 | 1.27x10^-1^ | 5.37x10^-2^ | 0.80 |
| rs12468353 | 162886230 | imputed | A | G | 0.19 | 0.19 | 1.01 | 9.29x10^-1^ | 5.62x10^-1^ | 0.77 |
| seq-NOVEL-10682 | 162893217 | imputed | A | G | 0.18 | 0.17 | 1.06 | 2.90x10^-1^ | 1.26x10^-1^ | 0.79 |
| imm_2_162893861 | 162893861 | imputed | A | G | 0.02 | 0.02 | 0.92 | 5.51x10^-1^ | 4.78x10^-1^ | 0.43 |
| imm_2_162896086 | 162896086 | imputed | G | A | 0.20 | 0.20 | 1.05 | 3.79x10^-1^ | 2.01x10^-1^ | 0.79 |
| rs4664465 | 162896691 | imputed | C | G | 0.02 | 0.02 | 0.87 | 3.80x10^-1^ | 3.75x10^-1^ | 0.69 |
| seq-NOVEL-10699 | 162899235 | imputed | A | G | 0.10 | 0.08 | 1.24 | 2.89x10^-3^ | 1.07x10^-3^ | 0.58 |
| rs11894889 | 162900874 | imputed | C | T | 0.02 | 0.02 | 1.00 | 9.94x10^-1^ | 9.59x10^-1^ | 0.67 |
| seq-NOVEL-10705 | 162901398 | imputed | A | C | 0.17 | 0.16 | 1.08 | 1.63x10^-1^ | 6.34x10^-2^ | 0.73 |
