# Supplementary material for: Admixture Mapping in Lupus Identifies Multiple Functional Variants within IFIH1 Associated with Apoptosis, Inflammation, and Autoantibody Production
Source: PLoS Genet. 2013 Feb 18;9(2):e1003222. doi: 10.1371/journal.pgen.1003222 (PMC3575474; doi:10.1371/journal.pgen.1003222)
Supplement: Table S7 — Imputation based association analysis for European Americans (N = 3968 cases; 9750 controls). ∧Rsq denotes the quality measure of the squared correlation between imputed and true genotypes. Pc stands for the P-value corrected for local ancestry. (DOCX) [file pgen.1003222.s013.docx]

**Table S7. Imputation based association analysis for European Americans** (N=3968 cases; 9750 controls). ^Rsq denotes the quality measure of the squared correlation between imputed and true genotypes. Pc stands for the P-value corrected for local ancestry.

| **SNP** | **Position** | **Status** | **A1** | **A2** | **Affected** | **Control** | **OR** | **P-value** | **Pc** | **Rsq^** |
| --- | --- | --- | --- | --- | --- | --- | --- | --- | --- | --- |
| rs2160710 | 162817515 | imputed | G | A | 0.01 | 0.01 | 1.11 | 3.89x10^-1^ | 3.83x10^-1^ | 0.78 |
| rs4664456 | 162818324 | imputed | A | T | 0.01 | 0.01 | 1.15 | 2.70x10^-1^ | 2.50x10^-1^ | 0.70 |
| rs2111485 | 162818782 | imputed | A | G | 0.37 | 0.40 | 0.90 | 1.83x10^-4^ | 1.43x10^-4^ | 0.94 |
| rs12464666 | 162819001 | imputed | T | A | 0.01 | 0.01 | 1.18 | 1.44x10^-1^ | 1.32x10^-1^ | 0.64 |
| rs12476601 | 162819166 | imputed | A | G | 0.01 | 0.01 | 1.12 | 3.56x10^-1^ | 3.53x10^-1^ | 0.76 |
| rs16846492 | 162820758 | imputed | A | G | 0.01 | 0.01 | 1.18 | 1.44x10^-1^ | 1.49x10^-1^ | 0.80 |
| rs10165813 | 162822162 | imputed | A | G | 0.01 | 0.01 | 1.19 | 1.23x10^-1^ | 1.27x10^-1^ | 0.80 |
| imm_2_162826473 | 162826473 | imputed | G | A | 0.01 | 0.01 | 0.94 | 6.39x10^-1^ | 6.37x10^-1^ | 0.82 |
| rs12467077 | 162830765 | imputed | A | G | 0.01 | 0.01 | 0.94 | 6.27x10^-1^ | 6.25x10^-1^ | 0.87 |
| rs1990760 | 162832297 | typed | G | A | 0.38 | 0.40 | 0.91 | 4.03x10^-4^ | 3.46x10^-4^ | 1.00 |
| rs3761652 | 162832400 | imputed | A | C | 0.01 | 0.01 | 1.18 | 1.47x10^-1^ | 1.47x10^-1^ | 0.92 |
| rs12474565 | 162833147 | typed | G | A | 0.02 | 0.01 | 1.21 | 7.54x10^-2^ | 7.86x10^-2^ | 1.00 |
| rs2111486 | 162836006 | imputed | A | G | 0.01 | 0.01 | 1.19 | 1.35x10^-1^ | 1.34x10^-1^ | 0.92 |
| rs3747518 | 162836971 | imputed | G | A | 0.02 | 0.01 | 1.21 | 7.54x10^-2^ | 7.86x10^-2^ | 1.00 |
| rs3747517 | 162837070 | typed | A | G | 0.26 | 0.28 | 0.93 | 1.27x10^-2^ | 1.15x10^-2^ | 1.00 |
| rs918161 | 162837914 | imputed | A | C | 0.01 | 0.01 | 1.16 | 2.04x10^-1^ | 1.91x10^-1^ | 0.85 |
| rs41463049 | 162838276 | imputed | A | C | 0.01 | 0.01 | 1.13 | 3.09x10^-1^ | 3.14x10^-1^ | 0.98 |
| rs10188109 | 162839750 | imputed | A | C | 0.02 | 0.01 | 1.22 | 6.98x10^-2^ | 7.30x10^-2^ | 1.00 |
| rs13388189 | 162840999 | imputed | G | A | 0.02 | 0.01 | 1.22 | 6.98x10^-2^ | 7.30x10^-2^ | 1.00 |
| rs4664053 | 162842928 | imputed | A | T | 0.01 | 0.01 | 1.23 | 6.45x10^-2^ | 6.36x10^-2^ | 0.94 |
| rs2287292 | 162844508 | typed | C | A | 0.02 | 0.01 | 1.20 | 1.09x10^-1^ | 1.07x10^-1^ | 0.99 |
| rs2287293 | 162844973 | imputed | C | G | 0.02 | 0.01 | 1.18 | 1.34x10^-1^ | 1.32x10^-1^ | 0.97 |
| rs7590692 | 162845017 | imputed | G | A | 0.02 | 0.01 | 1.14 | 2.25x10^-1^ | 2.08x10^-1^ | 0.70 |
| rs7603101 | 162845181 | imputed | G | A | 0.02 | 0.01 | 1.20 | 1.09x10^-1^ | 1.07x10^-1^ | 0.99 |
| rs16846553 | 162845705 | imputed | A | C | 0.01 | 0.01 | 1.09 | 4.35x10^-1^ | 4.17x10^-1^ | 0.74 |
| rs13431841 | 162845929 | imputed | A | G | 0.01 | 0.01 | 1.11 | 3.63x10^-1^ | 3.69x10^-1^ | 0.76 |
| rs16846555 | 162845994 | imputed | A | G | 0.01 | 0.01 | 1.23 | 6.45x10^-2^ | 6.36x10^-2^ | 0.95 |
| rs12479043 | 162846117 | imputed | G | c | 0.02 | 0.02 | 1.42 | 2.86x10^-4^ | 5.45x10^-4^ | 0.31 |
| rs10930046 | 162846229 | typed | G | A | 0.02 | 0.01 | 1.22 | 6.98x10^-2^ | 7.30x10^-2^ | 1.00 |
| rs6734769 | 162847031 | imputed | A | G | 0.02 | 0.02 | 1.06 | 5.81x10^-1^ | 5.83x10^-1^ | 0.87 |
| rs12464349 | 162847701 | imputed | A | G | 0.01 | 0.01 | 1.12 | 3.20x10^-1^ | 3.23x10^-1^ | 0.98 |
| rs12464391 | 162847812 | imputed | A | C | 0.02 | 0.01 | 1.19 | 1.09x10^-1^ | 1.12x10^-1^ | 1.00 |
| rs7602311 | 162848411 | typed | G | A | 0.02 | 0.01 | 1.19 | 1.09x10^-1^ | 1.12x10^-1^ | 1.00 |
| rs16846565 | 162849040 | typed | G | A | 0.01 | 0.01 | 1.10 | 4.10x10^-1^ | 4.11x10^-1^ | 1.00 |
| rs7567566 | 162849223 | imputed | G | A | 0.02 | 0.01 | 1.19 | 1.09x10^-1^ | 1.11x10^-1^ | 0.98 |
| rs2389683 | 162849559 | imputed | A | C | 0.02 | 0.01 | 1.18 | 1.28x10^-1^ | 1.30x10^-1^ | 0.78 |
| rs10179671 | 162849574 | imputed | G | A | 0.02 | 0.01 | 1.19 | 1.09x10^-1^ | 1.11x10^-1^ | 0.98 |
| rs4664460 | 162851670 | typed | G | A | 0.02 | 0.01 | 1.21 | 8.88x10^-2^ | 8.85x10^-2^ | 1.00 |
| rs7565446 | 162852642 | imputed | A | C | 0.02 | 0.01 | 1.17 | 1.40x10^-1^ | 1.29x10^-1^ | 0.84 |
| rs7591660 | 162852693 | imputed | G | A | 0.02 | 0.01 | 1.19 | 1.15x10^-1^ | 1.16x10^-1^ | 0.68 |
| rs6718470 | 162853608 | imputed | A | C | 0.02 | 0.01 | 1.17 | 1.58x10^-1^ | 1.60x10^-1^ | 0.88 |
| imm_2_162853712 | 162853712 | imputed | G | C | 0.01 | 0.01 | 1.04 | 7.23x10^-1^ | 7.23x10^-1^ | 0.87 |
| rs12466054 | 162854077 | imputed | A | G | 0.02 | 0.01 | 1.21 | 7.95x10^-2^ | 7.88x10^-2^ | 0.83 |
| rs4664461 | 162854648 | imputed | G | A | 0.02 | 0.01 | 1.21 | 8.88x10^-2^ | 8.85x10^-2^ | 0.99 |
| rs13313773 | 162854938 | imputed | A | T | 0.02 | 0.01 | 1.22 | 6.13x10^-2^ | 6.07x10^-2^ | 0.76 |
| rs12475321 | 162855424 | imputed | G | A | 0.02 | 0.01 | 1.24 | 5.54x10^-2^ | 5.73x10^-2^ | 0.95 |
| rs6758639 | 162856474 | imputed | G | A | 0.01 | 0.01 | 1.12 | 3.57x10^-1^ | 3.53x10^-1^ | 0.97 |
| rs12476567 | 162856657 | typed | C | A | 0.02 | 0.01 | 1.20 | 1.01x10^-1^ | 1.04x10^-1^ | 1.00 |
| rs4664462 | 162857710 | imputed | C | A | 0.01 | 0.01 | 1.15 | 2.20x10^-1^ | 2.17x10^-1^ | 0.85 |
| rs4664463 | 162858211 | imputed | G | A | 0.01 | 0.01 | 1.16 | 1.95x10^-1^ | 1.92x10^-1^ | 0.96 |
| rs974551 | 162859615 | imputed | G | A | 0.01 | 0.01 | 1.16 | 1.95x10^-1^ | 1.92x10^-1^ | 0.96 |
| rs12471127 | 162860058 | imputed | C | A | 0.01 | 0.01 | 1.11 | 3.96x10^-1^ | 3.92x10^-1^ | 0.85 |
| rs10439291 | 162860268 | imputed | A | G | 0.01 | 0.01 | 1.13 | 2.95x10^-1^ | 2.76x10^-1^ | 0.90 |
| rs10439256 | 162860597 | imputed | G | A | 0.01 | 0.01 | 1.16 | 1.95x10^-1^ | 1.92x10^-1^ | 0.95 |
| rs13023380 | 162862609 | typed | G | A | 0.43 | 0.48 | 0.84 | 9.52x10^-11^ | 5.97x10^-11^ | 1.00 |
| rs2163215 | 162863326 | imputed | A | C | 0.02 | 0.01 | 1.17 | 1.63x10^-1^ | 1.66x10^-1^ | 0.82 |
| rs73971815 | 162864104 | imputed | G | A | 0.02 | 0.01 | 1.19 | 1.11x10^-1^ | 1.14x10^-1^ | 0.99 |
| rs10195025 | 162866405 | imputed | A | G | 0.02 | 0.01 | 1.21 | 8.57x10^-2^ | 8.85x10^-2^ | 0.99 |
| rs12473955 | 162866771 | imputed | A | G | 0.02 | 0.01 | 1.22 | 7.18x10^-2^ | 7.43x10^-2^ | 0.94 |
| rs12479125 | 162867323 | imputed | G | A | 0.01 | 0.01 | 1.10 | 4.17x10^-1^ | 4.13x10^-1^ | 0.80 |
| rs16846600 | 162867580 | typed | G | A | 0.02 | 0.01 | 1.20 | 9.23x10^-2^ | 9.53x10^-2^ | 0.99 |
| rs12478636 | 162871862 | imputed | G | A | 0.01 | 0.01 | 1.08 | 5.33x10^-1^ | 5.29x10^-1^ | 0.83 |
| seq-NOVEL-10609 | 162873900 | imputed | G | A | 0.01 | 0.01 | 1.08 | 5.33x10^-1^ | 5.29x10^-1^ | 0.83 |
| rs17715343 | 162875992 | typed | G | C | 0.09 | 0.10 | 0.85 | 3.69x10^-4^ | 3.50x10^-4^ | 0.83 |
| rs73971820 | 162878207 | imputed | A | G | 0.01 | 0.01 | 1.12 | 3.22x10^-1^ | 3.24x10^-1^ | 0.72 |
| seq-NOVEL-10626 | 162878402 | imputed | G | A | 0.01 | 0.01 | 1.06 | 6.33x10^-1^ | 6.31x10^-1^ | 0.70 |
| rs62188193 | 162880074 | imputed | A | G | 0.02 | 0.01 | 1.78 | 8.67x10^-8^ | 1.47x10^-7^ | 0.21 |
| rs1864430 | 162880550 | imputed | C | A | 0.01 | 0.01 | 1.12 | 3.11x10^-1^ | 3.14x10^-1^ | 0.81 |
| imm_2_162881875 | 162881875 | imputed | T | A | 0.01 | 0.01 | 1.07 | 6.07x10^-1^ | 6.05x10^-1^ | 0.72 |
| rs11897697 | 162883902 | imputed | C | A | 0.02 | 0.01 | 1.22 | 7.90x10^-2^ | 8.01x10^-2^ | 0.78 |
| rs12472542 | 162886024 | imputed | G | A | 0.01 | 0.01 | 1.15 | 2.23x10^-1^ | 2.20x10^-1^ | 0.78 |
| rs12468353 | 162886230 | imputed | A | G | 0.01 | 0.01 | 1.15 | 2.23x10^-1^ | 2.20x10^-1^ | 0.77 |
| rs6730189 | 162886558 | imputed | A | G | 0.01 | 0.01 | 0.96 | 7.64x10^-1^ | 7.59x10^-1^ | 0.77 |
| rs11888158 | 162889948 | imputed | G | C | 0.01 | 0.01 | 1.18 | 1.42x10^-1^ | 1.43x10^-1^ | 0.79 |
| imm_2_162893861 | 162893861 | imputed | A | G | 0.01 | 0.01 | 1.13 | 3.02x10^-1^ | 3.04x10^-1^ | 0.68 |
| imm_2_162896086 | 162896086 | imputed | G | A | 0.01 | 0.01 | 1.16 | 1.89x10^-1^ | 1.94x10^-1^ | 0.70 |
| rs4664464 | 162896679 | imputed | G | A | 0.01 | 0.01 | 1.05 | 6.70x10^-1^ | 6.75x10^-1^ | 0.76 |
| rs4664465 | 162896691 | imputed | C | G | 0.01 | 0.01 | 0.98 | 8.64x10^-1^ | 8.54x10^-1^ | 0.71 |
| rs35544136 | 162897283 | imputed | G | C | 0.01 | 0.01 | 0.97 | 8.35x10^-1^ | 8.28x10^-1^ | 0.67 |
| rs11894889 | 162900874 | imputed | G | A | 0.01 | 0.01 | 0.98 | 8.93x10^-1^ | 8.85x10^-1^ | 0.70 |
| rs12468951 | 162902507 | imputed | A | G | 0.01 | 0.01 | 1.01 | 9.51x10^-1^ | 9.57x10^-1^ | 0.64 |
