# Supplementary material for: Admixture Mapping in Lupus Identifies Multiple Functional Variants within IFIH1 Associated with Apoptosis, Inflammation, and Autoantibody Production
Source: PLoS Genet. 2013 Feb 18;9(2):e1003222. doi: 10.1371/journal.pgen.1003222 (PMC3575474; doi:10.1371/journal.pgen.1003222)
Supplement: Table S8 — Risk allele frequencies and Fst values between populations for three IFIH1 SNPs. Local ancestry at three SNPs in IFIH1 was estimated for AA. Individuals whose ancestral state was European (N = 129) and African (N = 2124) were selected, and their risk allele (“A”) frequency compared with allele frequencies in CEPH and YRI. FST was calculated between these groups. (DOCX) [file pgen.1003222.s014.docx]

**Table S8. Risk allele frequencies and Fst values between populations for three *IFIH1* SNPs.** Local ancestry at three SNPs in *IFIH1* was estimated for AA. Individuals whose ancestral state was European (N=129) and African (N=2124) were selected, and their risk allele (“A”) frequency compared with allele frequencies in CEPH and YRI. F_ST_ was calculated between these groups.

| SNP | Group | **Risk Allele (A) Frequency** | Fst | | | |
| --- | --- | --- | --- | --- | --- | --- |
|  |  |  | **African Haplotypes** | **European Haplotypes** | **CEPH** | **YRI** |
| rs1990760 | **African Haplotypes** | 0.066 | 0 | -- | -- | -- |
|  | **European Haplotypes** | 0.628 | 0.689 | 0 | -- | -- |
|  | **CEPH** | 0.621 | 0.662 | 0 | 0 | -- |
|  | **YRI** | 0.068 | 0 | 0.538 | 0.494 | 0 |
| rs10930046 | **African Haplotypes** | 0.5 | 0 | -- | -- | -- |
|  | **European Haplotypes** | 0.991 | 0.336 | 0 | -- | -- |
|  | **CEPH** | 0.987 | 0.332 | 0 | 0 | -- |
|  | **YRI** | 0.442 | 0.005 | 0.512 | 0.526 | 0 |
| rs13023380 | **African Haplotypes** | 0.007 | 0 | -- | -- | -- |
|  | **European Haplotypes** | 0.495 | 0.856 | 0 | -- | -- |
|  | **CEPH** | 0.504 | 0.822 | 0 | 0 | -- |
|  | **YRI** | 0.003 | 0 | 0.542 | 0.488 | 0 |
