# Supplementary material for: Admixture Mapping in Lupus Identifies Multiple Functional Variants within IFIH1 Associated with Apoptosis, Inflammation, and Autoantibody Production
Source: PLoS Genet. 2013 Feb 18;9(2):e1003222. doi: 10.1371/journal.pgen.1003222 (PMC3575474; doi:10.1371/journal.pgen.1003222)
Supplement: Table S9 — Alignment of genomic region surrounding rs1990760 for available mammal genomes. The base corresponding to rs1990760 is nearly universally conserved as “G”, with a resulting alanine codon. Sequence is shown reverse complement, in the direction of the reading frame, showing five codons in either direction. Squirrel may have a threonine (Thr) side-chain; interestingly, tenrec may have a 2-amino acid deletion. Alignment of Chr2: 163124034–163124066, 33 bps (reverse complement). (DOCX) [file pgen.1003222.s015.docx]

**Table S9. Alignment of genomic region surrounding rs1990760 for available mammal genomes.** The base corresponding to rs1990760 is nearly universally conserved as “G”, with a resulting alanine codon. Sequence is shown reverse complement, in the direction of the reading frame, showing five codons in either direction. Squirrel may have a threonine (Thr) side-chain; interestingly, tenrec may have a 2-amino acid deletion. Alignment of Chr2: 163124034 - 163124066, 33 bps (reverse complement).

| **Species** | Scientific name | Version | 5’ sequence | codon | 3’ sequence |
| --- | --- | --- | --- | --- | --- |
| **Human** | *Homo sapiens* | hg19/GRCh37 | GTAAGAGAAAACAAA | **G**CA | CTGCAAAAGAAGTGT |
| **Chimp** | *Pan troglodytes* | panTro2 | GTAAGAGAAAACAAA | **G**CA | CTGCAAAAGAAGTGT |
| **Gorilla** | *Gorilla gorilla gorilla* | gorGor1 | GTAAGAGAGAACAAA | **G**CA | CTGCAAAAGAAGTGT |
| **Orangutan** | *Pongo pygmaeus abelii* | ponAbe2 | GTAAGAGAAAACAAA | **G**CA | CTGCAAAAGAAGTGT |
| **Rhesus** | *Macaca mulatta* | rheMac2 | GTAAGAGAAAATAAA | **G**CC | CTGCAAAAGAAGTGT |
| **Baboon** | *Papio hamadryas* | papHam1 | GTAAGAGAAAACAAA | **G**CC | CTGCAAAAGAAGTGT |
| **Gibbon** | *Nomascus leucogenys* | NCBI trace archive | GTAAGAGAAAACAAA | **G**CA | CTGCAAAAGAAGTGT |
| **Marmoset** | *Callithrix jacchus* | calJac1 | GTGAGAGAAAACAAA | **G**CA | CTGCAAAAGAAGTTT |
| **Tarsier** | *Tarsier syrichta* | tarSyr1 | GTGAGAGAAAACAAA | **G**CA | TTGCAAAAAAAGTTT |
| **Bushbaby** | *Otolemur garnettii* | otoGar1 | ATGAGAGAAAACAAA | **G**CT | CTGAAAGAGAAGTTT |
| **Tree shrew** | *Tupaia belangeri* | tupBel1 | GTGAGAGAAAACAAA | **G**CA | CTACAAGAGAAGTTT |
| **Mouse** | *Mus musculus* | mm9 | GTAAGAGAAAACAAA | **G**CA | CTGCAAAAGAAATTT |
| **Rat** | *Rattus norvegicus* | rn4 | GTGAGAGAGAACAAA | **G**CA | CTACAAATGAAATTT |
| **Naked mole rat** | *Heterocephalus glaber* | NCBI trace archive | GTGAGAGAAAACAAA | **G**CA | CTGCAAAATAAGTTT |
| **Guinea pig** | *Cavia porcellus* | cavPor3 | ACAAGAGAAAACAAA | **G**CA | CTGCAAAGTAAGTTC |
| **Squirrel** | *Spermophilus tridecemlineatus* | speTri1 | GTGACAGAAAACAAA | **A**CT | CTGCAGAAGAAGTTT |
| **Rabbit** | *Oryctolagus cuniculus* | oryCun2 | GTGAGAGAAAGCAAA | **G**CA | CAAAAGAAGAACTTT |
| **Alpaca** | *Vicugna pacos* | vicPac1 | GTGAGAGGAAACAGA | **G**CA | CTGCAAAACAGGTTT |
| **Dolphin** | *Tursiops truncatus* | turTru1 | GTGAGAGGAAACAAA | **G**CA | CTGCAAACAAAGTTT |
| **Cow** | *Bos taurus* | bosTau4 | GTGAGAGGAAACAAA | **G**CA | CTGCAAACAATGTGT |
| **Pig** | *Sus scrufa* | NCBI trace archive | GTGAGAGGAAACAAG | **G**CA | CTGCAGACGAAGTTT |
| **Horse** | *Equus caballus* | equCab2 | GTGAGAGAAAACAAA | **G**CA | CTGAAAAAGAAGTTT |
| **Cat** | *Felis catus* | felCat3 | GTGAGAGAAAACAAA | **G**CC | CTACGAAAGAAGTTT |
| **Dog** | *Canis lupus familiarus* | canFam2 | GTGAGAGAAAACAAA | **G**CA | CTACGAAAGAAGTTT |
| **Microbat** | *Myotis lucifugus* | myoLuc1 | GTGAGAGAAAACAAA | **G**CA | CTGCAAAAGAAGTTT |
| **Megabat** | *Pteropus vampyrus* | pteVam1 | ATGAGAGAAAACAAA | **G**CA | CTGAAAAAGAAGTTT |
| **Hedgehog** | *Erinaceus europaeus* | eriEur1 | GTGATAGAAAACAAA | **G**CA | CTGAAAAAGAAATTT |
| **Shrew** | *Sorex araneus* | sorAra1 | GTCATAGAAAACAAA | **G**CA | CTAAAAGAAAAGTTT |
| **Elephant** | *Loxodonta africana* | loxAfr3 | GTGAGAGAAAACAAA | **G**CA | TTGCAAAAAAAGTTT |
| **Panda** | *Ailuropoda melanoleuca* | NCBI | GTGAGAGAAAACAAA | **G**CA | CTACGGAAGAAATTT |
| **Tenrec** | *Echinops telfairi* | echTel1 | GTGAGAGAAGGCACG | --- | ---AAAAGCAAGTTT |
| **Armadillo** | *Dasypus novemcinctus* | dasNov2 | GTGAGAGAAAACAAA | **G**CA | TTGCAAGAGAAGTTT |
| **Sloth** | *Choloepus hoffmanni* | choHof1 | GTGAGAGAAAACAAA | **G**CA | CTACAAGAGAAGTTT |
| **Wallaby** | *Macropus eugenii* | macEug1 | GTAAGAGAAAATAAA | **G**CA | TTGCAAAAGAAATTC |
| **Opossum** | *Monodelphis domestica* | monDom5 | GTAAGAGAAAATAAA | **G**CA | CTACAAAAGAAATTT |
