# Supplementary material for: Admixture Mapping in Lupus Identifies Multiple Functional Variants within IFIH1 Associated with Apoptosis, Inflammation, and Autoantibody Production
Source: PLoS Genet. 2013 Feb 18;9(2):e1003222. doi: 10.1371/journal.pgen.1003222 (PMC3575474; doi:10.1371/journal.pgen.1003222)
Supplement: Table S10 — Alignment of genomic region surrounding rs10930046 for available vertebrate genomes. The base corresponding to rs10930046 is universally conserved as “G”, with a resulting arginine codon. Sequence is shown reverse complement, in the direction of the reading frame, showing five codons in either direction. Alignment of Chr2: 163137967–163137999, 33 bps (reverse complement). (DOCX) [file pgen.1003222.s016.docx]

**Table S10**. **Alignment of genomic region surrounding rs10930046 for available vertebrate genomes.** The base corresponding to rs10930046 is universally conserved as “G”, with a resulting arginine codon. Sequence is shown reverse complement, in the direction of the reading frame, showing five codons in either direction. Alignment of Chr2: 163137967 - 163137999, 33 bps (reverse complement).

| **Species** | Scientific name | Version | 5’ sequence | codon | 3’ sequence |
| --- | --- | --- | --- | --- | --- |
| **Human** | *Homo sapiens* | hg19/GRCh37 | AATAACATCATGAGG | C**A**T | TATTTGATGCAGAAG |
| **Chimp** | *Pan troglodytes* | panTro2 | AATAACATCATGAGG | C**G**T | TATTTGATGCAGAAG |
| **Gorilla** | *Gorilla gorilla gorilla* | gorGor1 | AATAACATCATGAGG | C**G**T | TATTTGATGCAGAAG |
| **Orangutan** | *Pongo pygmaeus abelii* | ponAbe2 | AATAACATCATGAGG | C**G**T | CATTTGATGCAGAAG |
| **Rhesus** | *Macaca mulatta* | rheMac2 | AATAACATCATGAGG | C**G**T | TATTTGATGCAGAAG |
| **Baboon** | *Papio hamadryas* | papHam1 | AATAACATTATGAGG | C**G**T | TATTTGATGCAGAAG |
| **Gibbon** | *Nomascus leucogenys* | NCBI trace archive | AATAACATCATGAGG | C**G**T | TATTTGATGCAGAAG |
| **Marmoset** | *Callithrix jacchus* | calJac1 | AATAACATCATGAGG | C**G**T | TATTTGAAGCAGAAG |
| **Tarsier** | *Tarsier syrichta* | tarSyr1 | AATAACATCATGAGG | C**G**T | TATTTGAAACAGAAG |
| **Mouse lemur** | *Microcebus murinus* | NCBI trace archive | AACAACATTATGAGG | C**G**T | TACTTGAAACAGAAG |
| **Bushbaby** | *Otolemur garnettii* | otoGar1 | AACAACATTATGAGG | C**G**T | TATGTGAAACAGAAG |
| **Tree shrew** | *Tupaia belangeri* | tupBel1 | AATAACATCATGAGG | C**G**T | TACTTGAAACAGAAG |
| **Mouse** | *Mus musculus* | mm9 | AACAACATCATGAGA | C**G**A | TATTTGAAGCAGAAG |
| **Rat** | *Rattus norvegicus* | rn4 | AACAACATCATGAGA | C**G**G | TATTTGAAGCAGAAG |
| **Naked mole rat** | *Heterocephalus glaber* | NCBI trace archive | AACAACATCATGAGG | C**G**T | TACATGAAACAGAAG |
| **Kangaroo rat** | *Dipodomys ordii* | NCBI trace archive | AACAACATCATGAGG | C**G**T | TATTTGAAACAGAAG |
| **Guinea pig** | *Cavia porcellus* | cavPor3 | AACAACATCATGAGG | C**G**T | TATCTGAAACAGAAG |
| **Rabbit** | *Oryctolagus cuniculus* | oryCun2 | AATAACATTATGAGG | C**G**T | TATTTGAAACAGAAG |
| **Pika** | *Ochotona princeps* | ochPri2 | AATAACATTATGAGG | C**G**T | TATCTGAAGCAGAAG |
| **Alpaca** | *Vicugna pacos* | vicPac1 | AATAACATCATGAGG | C**G**T | TATTTGAAACAGAAA |
| **Dolphin** | *Tursiops truncatus* | turTru1 | AATAACATCATGAGG | C**G**T | TATTTGAACCAGAAG |
| **Cow** | *Bos taurus* | bosTau4 | AACAACATCATGAGG | C**G**T | TTTTTGAAACAGAAG |
| **Pig** | *Sus scrufa* | NCBI trace archive | AATAACATCATGAGG | C**G**T | TATTTGAAACAGAAA |
| **Sheep** | *Ovis aries* | NCBI | AACAACATCATGAGG | C**G**T | TATTTGAAACAGAAA |
| **Horse** | *Equus caballus* | equCab2 | AATAACATCATGAGG | C**G**T | TATTTGAAACAGAAG |
| **Cat** | *Felis catus* | felCat3 | AATAACATCATGAGG | C**G**T | TACTTGAAACAGAAG |
| **Dog** | *Canis lupus familiarus* | canFam2 | AATAACATCATGAGA | C**G**T | TACTTGAAACAGAAG |
| **Ferret** | *Mustela furo* | NCBI | AATAACATCATGAGG | C**G**T | TACTTGAAACAGAAG |
| **Microbat** | *Myotis lucifugus* | myoLuc1 | AATAACATCATGAGG | C**G**T | TATTTGAAACAGAAG |
| **Megabat** | *Pteropus vampyrus* | pteVam1 | AATAACATCATGAGG | C**G**T | TATTTGAAACAGAAG |
| **Hedgehog** | *Sorex araneus* | sorAra1 | AACAACATCATGAGG | C**G**T | TACTTGACACAGAAA |
| **Shrew** | *Loxodonta africana* | loxAfr3 | AATAACATCATGAGA | C**G**T | TATATAGTGCAGAAG |
| **Elephant** | *Myotis lucifugus* | myoLuc1 | AATAACATCATGAGG | C**G**C | TATTTGGAACAGAAG |
| **Rock hyrax** | *Procavia capensis* | NCBI trace archive | AATAACATCATGAGG | C**G**T | TATTTGAAACAGAAG |
| **Tenrec** | *Echinops telfairi* | echTel1 | AATACCATCATGCGG | C**G**C | TATTTGAAACAGAAG |
| **Sloth** | *Choloepus hoffmanni* | choHof1 | AATAACATCATGAGG | C**G**T | TACCTGAAACAGAAG |
| **Wallaby** | *Macropus eugenii* | macEug1 | AATAATATAATGACA | C**G**T | TATTTGAAACAAAAA |
| **Opossum** | *Monodelphis domestica* | monDom5 | AATACTATAATGAGA | C**G**T | TACGTGAAGCAAAAG |
| **Platypus** | *Ornithorhynchus anatinus* | ornAna1 | AACAATATAATGACC | C**G**C | TATTTGACACAAAAG |
| **Chicken** | *Gallus gallus* | galGal3 | AACAATATCATGCGA | C**G**T | TACTTAAAAGAAAAG |
| **Turkey** | *Meleagris gallopavo* | NCBI trace archive | AACAATATCATGCGA | C**G**T | TACTTAAAAGATAAG |
| **Zebra finch** | *Taeniopygia guttata* | taeGut1 | AATAATATAATGAGA | C**G**T | TACTTAAAAGAAAAG |
| **Lizard** | *Anolis carolinensis* | anoCar1 | AACAACATCATGCGC | C**G**G | TATTTGAAACAAAAG |
| **X. tropicalis** | *Xenopus tropicalis* | xenTro2 | AATAATATCATGATC | C**G**T | TATATAAAAAAGAAA |
| **Tetraodon** | *Tetraodon nigroviridis* | tetNig2 | AACCAGATCATGGTG | C**G**A | TACCTTATGCAGAAG |
| **Fugu** | *Takifugu rubripes* | fr2 | AACGAGATCATGGTG | C**G**C | TACCTGAAGCAGAAG |
| **Stickleback** | *Gasterosteus aculeatus* | gasAcu1 | AACCACATAATGATG | C**G**C | TATCTGGCGCAGAAG |
| **Medaka** | *Oryzias latipes* | oryLat2 | AACCACATAATGATG | C**G**A | TATCTGAAGCAGAAA |
| **Zebrafish** | *Danio rerio* | danRer6 | AACCACATCATGATT | C**G**C | TACCTGAAACAGAAG |
| **Lamprey** | *Petromyzon marinus* | petMar1 | AACAAGATTATGTAT | C**G**C | TACTTGGAGGATAAA |
