# Supplementary material for: Admixture Mapping in Lupus Identifies Multiple Functional Variants within IFIH1 Associated with Apoptosis, Inflammation, and Autoantibody Production
Source: PLoS Genet. 2013 Feb 18;9(2):e1003222. doi: 10.1371/journal.pgen.1003222 (PMC3575474; doi:10.1371/journal.pgen.1003222)
Supplement: Table S11 — Mass Spectrometry sequencing and MASCOT database searches of 2D-gel and streptavidin agarose beads of EMSA. (DOCX) [file pgen.1003222.s017.docx]

**Table S11. Mass Spectrometry sequencing and MASCOT database searches of 2D-gel and streptavidin agarose beads of EMSA.**

| **Method** | **Gene name** | **Gene description** | **Organism** | **Symbol** | **Mass** | **Score** | **Queries matched** |
| --- | --- | --- | --- | --- | --- | --- | --- |
| 2-D gel | XRCC5_HUMAN | X-ray repair cross-complementing protein 5 (lupus Ku70 autoantigen) | *Homo sapiens* | XRCC5 | 83222 | 2310 | 46 |
|  | XRCC6_HUMAN | X-ray repair cross-complementing protein 6 (lupus Ku80 autoantigen) | *Homo sapiens* | XRCC6 | 70084 | 2266 | 42 |
|  | HS90A_BOVIN | Heat shock protein HSP 90-alpha | *Bos taurus* | HSP90AA1 | 85077 | 1334 | 23 |
|  | ACTB_BOVIN | Actin, cytoplasmic 1 | *Bos taurus* | ACTB | 42052 | 1042 | 18 |
|  | HS90B_HORSE | Heat shock protein HSP 90-beta | *Eqqus caballus* | HSP90AB1 | 83527 | 906 | 18 |
|  | ACPH_HUMAN | Acylamino-acid-releasing enzyme | *Homo sapiens* | APEH | 82142 | 664 | 15 |
|  | NUCL_HUMAN | Nucleolin | *Homo sapiens* | NCL | 76625 | 528 | 11 |
|  | A1AT_BOVIN | Alpha-1-antiproteinase | *Bos taurus* | SERPINA1 | 46417 | 256 | 4 |
|  | ACT17_DICDI | Actin-17 | *Dictyostelium discoideum* | ACT17 | 41773 | 230 | 7 |
|  | EF1D_HUMAN | Elongation factor 1-delta | *Homo sapiens* | EEF1D | 31217 | 176 | 3 |
|  | TSN_BOVIN | Translin | *Bos taurus* | TSN | 33206 | 115 | 5 |
|  | HNRPK_BOVIN | Heterogeneous nuclear ribonucleoprotein K | *Bos taurus* | HNRNPK | 51272 | 81 | 2 |
| Streptavidin | NUCL_HUMAN (1st band) | Nucleolin | *Homo sapiens* | NCL | 76625 | 1104 | 36 |
|  | HS90B_HUMAN (2nd band) | Heat shock protein HSP 90-beta | *Homo sapiens* | HSP90AB1 | 83554 | 1226 | 28 |
|  | MCM6_BOVIN | DNA replication licensing factor MCM6 | *Bos taurus* | MCM6 | 93842 | 76 | 3 |
|  | YKC2_CAEEL | Uncharacterized protein B0280.2 | *Caenorhabditis elegans* | B0280.2 | 70677 | 59 | 1 |
|  | EF2_BOVIN | Elongation factor 2 | *Bos taurus* | EEF2 | 96276 | 51 | 1 |
