# Supplementary material for: Admixture Mapping in Lupus Identifies Multiple Functional Variants within IFIH1 Associated with Apoptosis, Inflammation, and Autoantibody Production
Source: PLoS Genet. 2013 Feb 18;9(2):e1003222. doi: 10.1371/journal.pgen.1003222 (PMC3575474; doi:10.1371/journal.pgen.1003222)
Supplement: Text S1 — Additional list of investigators. (DOCX) [file pgen.1003222.s018.docx]

**Admixture Mapping in Lupus Identifies Multiple Functional Variants within IFIH1 Associated with Apoptosis, Inflammation and Autoantibody Production**

**Short Title: Admixture Mapping, *IFIH1* and SLE susceptibility**

Julio E. Molineros^1,24^, Amit K. Maiti^1,24^, Celi Sun^1,24^, Loren L. Looger^2^, Shizhong Han^1,3^, Xana Kim-Howard^1^, Stuart Glenn^1^, Adam Adler^1^, Jennifer A. Kelly^1^, Timothy B. Niewold^4^, Gary S. Gilkeson^5^, Elizabeth E. Brown^6^, Graciela S. Alarcón^6^, Jeffrey C. Edberg^6^, Michelle Petri^7^, Rosalind Ramsey-Goldman^8^, John D. Reveille^9^, Luis M. Vilá^10^, Barry I. Freedman^11^, Betty P. Tsao^12^, Lindsey A. Criswell^13^, Chaim O. Jacob^14^, Jason H. Moore^15^, Timothy J. Vyse^16,17^, Carl L. Langefeld^18^, Joel M. Guthridge^1^, Patrick M. Gaffney^1^, Kathy L. Moser^1,19^, R. Hal Scofield^1^, Marta E. Alarcón-Riquelme^1,20^ on behalf of the BIOLUPUS Network, Scott M. Williams^21^, Joan T. Merrill^22^, Judith A. James^1,19^, Kenneth M. Kaufman^1^, Robert P. Kimberly^6^, John B. Harley^23^, Swapan K. Nath^1^

**Affiliations**

^1^Arthritis and Clinical Immunology Research Program, Oklahoma Medical Research Foundation, Oklahoma City, Oklahoma, USA. ^2^Howard Hughes Medical Institute, Janelia Farm Research Campus, Ashburn, Virginia, USA. ^3^Department of Psychiatry, Yale School of Medicine, CT, USA. ^4^Section of Rheumatology and Gwen Knapp Center for Lupus and Immunology Research, University of Chicago, Chicago, Illinois, USA. ^5^Division of Rheumatology, Medical University of South Carolina, Charleston, South Carolina, USA. ^6^Department of Medicine, University of Alabama at Birmingham, Birmingham, Alabama, USA. ^7^Department of Medicine, Johns Hopkins University School of Medicine, Baltimore, Maryland, USA. ^8^Division of Rheumatology, Northwestern University Feinberg School of Medicine, Chicago, Illinois, USA. ^9^Rheumatology and Clinical Immunogenetics, University of Texas Health Science Center at Houston, Houston, Texas, USA. ^10^Department of Medicine, Division of Rheumatology, University of Puerto Rico Medical Sciences Campus, San Juan, Puerto Rico. ^11^Department of Internal Medicine, Wake Forest School of Medicine, Winston-Salem, North Carolina, USA. ^12^Division of Rheumatology, Department of Medicine, University of California Los Angeles, Los Angeles, California, USA. ^13^Rosalind Russell Medical Research Center for Arthritis, University of California San Francisco, San Francisco, California, USA. ^14^Department of Medicine, University of Southern California, Los Angeles, California, USA. ^15^Department of Genetics, Dartmouth Medical School, Lebanon, New Hampshire, USA. ^16^Division of Genetics and Molecular Medicine, King's College London, London, UK. ^17^Division of Immunology, Infection and Inflammatory Diseases, Kings College London, London, UK. ^18^Department of Biostatistical Sciences, Wake Forest University Health Sciences, Wake Forest, North Carolina, USA. ^19^College of Medicine, University of Oklahoma Health Sciences Center, Oklahoma City, Oklahoma, USA. ^20^Centro de Genómica e Investigación Oncológica (GENyO) - Pfizer/Universidad de Granada/Junta de Andalucía, Granada, Spain. ^21^Department of Genetics,Geisel School of Medicine Dartmouth College, Hanover, New Hampshire, USA^.^ ^22^Clinical Pharmacology Research Program, Oklahoma Medical Research Foundation, Oklahoma City, Oklahoma, USA. ^23^Cincinnati Children's Hospital Medical Center and the US Department of Veterans Affairs Medical Center, Cincinnati, Ohio, USA. ^24^These authors contributed equally to this work.

**Address for Correspondence**

Swapan K. Nath, Ph.D.

Arthritis and Clinical Immunology Research Program

Oklahoma Medical Research Foundation

1025 N.E. 13^th^ Street

Oklahoma City, OK 73104

Phone: 405-271-7765

Fax: 405-271-4110

Email: Swapan-Nath@omrf.org

**TEXT S1. AdditionAL List of INVESTIGATORS**

The BIOLUPUS network, which provided samples of European ancestry, includes the following individuals and their affiliations:

Johan Frostegård^1^, MD, PhD, Lennart Truedsson^2^, MD, PhD, Enrique de Ramón^3^, PhD, José M. Sabio^4^, MD, PhD, María F. González-Escribano^5^, PhD, Bernardo A. Pons-Estel^6^, MD, Sandra D’Alfonso^7^, PhD, Torsten Witte^8^, MD, PhD, Bernard R. Lauwerys^9^, MD, PhD, Emoke Endreffy^10^, PhD, László Kovács^11^, MD, PhD, Carlos Vasconcelos^12^, MD, PhD, Berta Martins da Silva^13^, PhD, Javier Martin^14^, MD, PhD

^1^Department of Medicine, Karolinska University Hospital, Huddinge, 14183, Stockholm, Sweden; ^2^Department of Laboratory Medicine, section of M.I.G., Lund University, 221 00, Lund, Sweden; ^3^Hospital Carlos Haya, 29009, Málaga, Spain; ^4^Hospital Virgen de las Nieves, 18001, Granada, Spain; ^5^Hospital Virgen del Rocío, 41013, Sevilla, Spain; ^6^Department of Rheumatology, Sanatorio Parque, Rosario, 2000, Argentina; ^7^Department of Medical Sciences and IRCAD, University of Eastern Piedmont, 28100, Novara, Italy;^8^Hannover Medical School, 30625, Hannover, Germany; ^9^Cliniques Universitaires Saint-Luc, Université catholique de Louvain, B-1200, Bruxells, Belgium; ^10^Department of Pediatrics and Health Center,University of Szeged, H-6721, Szeged, Hungary; ^11^Department of Rheumatology, Albert Szent-Györgyi Clinical Centre, University of Szeged, H-6725, Szeged, Hungary; ^12^Hospital Santo Antonio and ICBAS, 4099, Porto, Portugal; ^13^UMIB/ICBAS, Immunogenetics laboratory, Universidado do Porto, 4099-003, Porto, Portugal; ^14^Instituto de Biomedicina y Parasitología López-Neyra, CSIC, Granada, Spain.
